# Multifactor exploration and multi-objective optimization of trapezoidal threads

**DOI:** 10.1038/s41598-025-94144-5

**Published:** 2025-04-08

**Authors:** Yongchao Zhou, Zhihua Yan, Peng Zhou, Yanping Zheng

**Affiliations:** 1 School of Mechanical and Power Engineering, Zhengzhou University, Zhengzhou, 450066 Henan China; 2 Hunan University of Science and Technology, Xiangtan, 411201 Hunan China

**Keywords:** Trapezoidal threads, Dynamics, Orthogonal experiments, Modal frequencies, Engineering, Mechanical engineering

## Abstract

Trapezoidal thread, as a key component in mechanical transmission, is widely used in many industries, and its parameter optimization is crucial to enhance the performance of threaded transmission mechanism. Taking the screw nut mechanism of a company’s screw elevator as an entry point, this paper thoroughly researches the specific effects of the four main parameters of trapezoidal threads—pitch, number of thread heads, tooth angle and tooth height—on the structural performance. Dynamic simulation using ANSYS software, combined with orthogonal experimental design, systematically analyzed the role of these parameters on the dynamic performance of structural components. Through the polar analysis, the order of importance of each parameter was determined, and the modal frequency and transport efficiency were introduced as the optimization objectives, and the optimal parameter combination scheme was finally derived. The results show that the optimized structural equivalent stress is reduced by 29.1%, the first-order modal frequency is increased by 24.5%, and the transport rate is increased by 11.1%. This study enriches the results in the field of threading and provides theoretical support for the future development and safe application of trapezoidal threads in screw drives.

## Introduction

Trapezoidal thread is a thread shape consisting of upper and lower trapezoidal surfaces with isosceles trapezoidal teeth, which has the advantages of high tightening force and low damaging properties, and is used in the automotive industry in important parts such as steering mechanism, suspension system, braking system, transmission, etc., as well as in the connection of various medical devices and in the aviation industry in the key parts such as the hydraulic system and the connection of the mechanisml^1^. Trapezoidal threads provide reliable connection and transmission solutions in critical parts such as the power transmission part of home elevators and the propulsion system and rudder system part of ships^2–5^. At present, the research on trapezoidal threads by scholars at home and abroad mainly focuses on the following aspects: the degree of robustness and durability of trapezoidal threads, the manufacturing method^6–8^, and how to make it more reasonably designed, etc. These researches provide important theoretical foundations and practical experience for the in-depth investigation and optimization of trapezoidal threads.

Although trapezoidal threads provide stable fastening force and torque transmission in helical drives with their unique tooth design, they face difficult machining, self-locking limitations^9–11^ and lubrication wear problems, vibration and noise challenges^12,13^ The problems and challenges include difficult selection of materials and limitations in transmission efficiency.

**Fig. 1:**
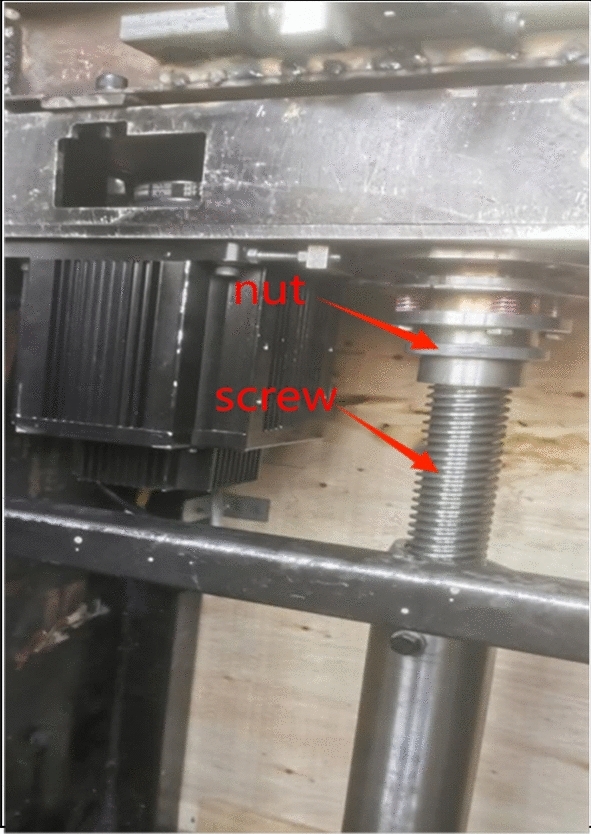
Physical drawing of trapezoidal thread structure.

Liu et al.^14^ conducted a parametric kinetic study on the pitch of trapezoidal threads; Acharyaet al.^15^ have analyzed the forces on threaded structures in planetary wheels with threaded structures compared to roller structures in machine tools to generate medium linear motion; Jia et al.^16^ conducted an optimization study on the mesh convergence analysis and load distribution of threaded teeth for the static simulation of common threaded structures;

The author conducted a theoretical analysis of the trapezoidal thread structure for self-locking, equivalent friction angle and force in the bearing process in the helical drive, and analyzed and studied its common influencing factors of pitch, number of thread heads, trapezoidal thread tooth angle and tooth height under different level parameters. The trapezoidal thread parts of a company’s lifting equipment and their working conditions serve as the research focus (as illustrated in Fig. 1). The analysis of these trapezoidal thread components within structural assemblies is conducted through orthogonal experiments^17^, utilizing a designated design methodology^18^. The objective is to achieve a multi-objective optimization design of trapezoidal threads in structural components, focusing on the key influencing factor dimensions.The design of the trapezoidal threads in structural parts is optimized by orthogonal experimental design. The 1st order modal frequency^19^ parameter to extend the service life of the support system and the transportation efficiency parameter to increase the working efficiency of the system, reduce the economic burden of customers and increase the competitiveness of products. The structure, data and related process parameters of the orthogonal experimental combination program obtained in the course of the research can provide a theoretical basis for the actual production and application of products with trapezoidal thread structure, improve the level of equipment manufacturing design and equipment safety, and reduce the working cost of the screw elevator.

## Theoretical research analysis

### Trapezoidal thread self-locking condition analysis

Self-locking is one of the main characteristics of threaded mechanisms, a characteristic that makes them often used in equipment with high safety requirements. The self-locking characteristic of screw drives is related to the equivalent friction angle, rather than a general friction factor. Starting from the definition of torque^20^, the input torque of the screw is equal to the product of the tangential force P acting on the middle diameter d_2_ of the screw and half of the middle diameter d2 of the screw:

1 $$M = P * \frac{{d_{2} }}{2}$$

where d_2_ is the screw center diameter,P is the tangential force acting on the screw center diameter d_2_.

**Fig. 2:**
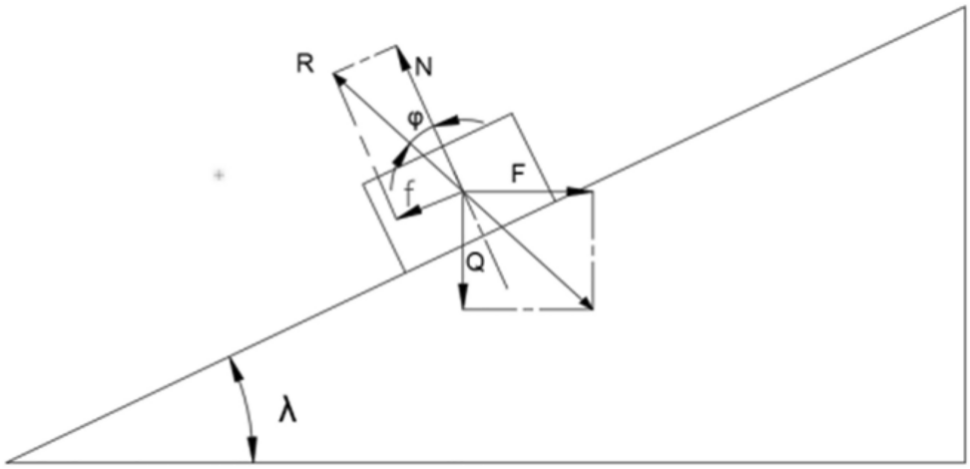
Simplified schematic diagram of the force.

The nut movement is simplified as shown in the following Fig. 2: the movement of the trapezoidal thread structure nut and screw can be viewed as a nut on the inclined plane at an angle λ, being pulled upward by F. λ is the helical angle of lift, Q is the pressure on the nut, N is the support force on the nut from the screw teeth. f is the friction force on the nut, and R is the joint force of N and f.

The force analysis can be obtained:

2 $$P = Q * \tan (\lambda + \phi )$$

where λ is the helical rise angle, Q is the pressure on the nut, N is the support force on the nut by the screw threads. f is the friction force on the nut, and R is the combined force of N and f, $\phi$ is the equivalent friction angle and the equivalent friction coefficient; ${\text{tan}}\lambda = \frac{s}{{\pi * d_{2} }}$, $s = k * t$, s is the thread lead, k is the number of threads, and t is the thread pitch. In this way the inverse trigonometric function is utilized to find λ. Equivalent coefficient of friction:

3 $$\mu = \tan \phi = \frac{f}{N}$$

where $\phi$ is the equivalent friction angle and the equivalent friction coefficient is equal to the ratio of friction force $f$ to pressure N.

**Fig. 3:**
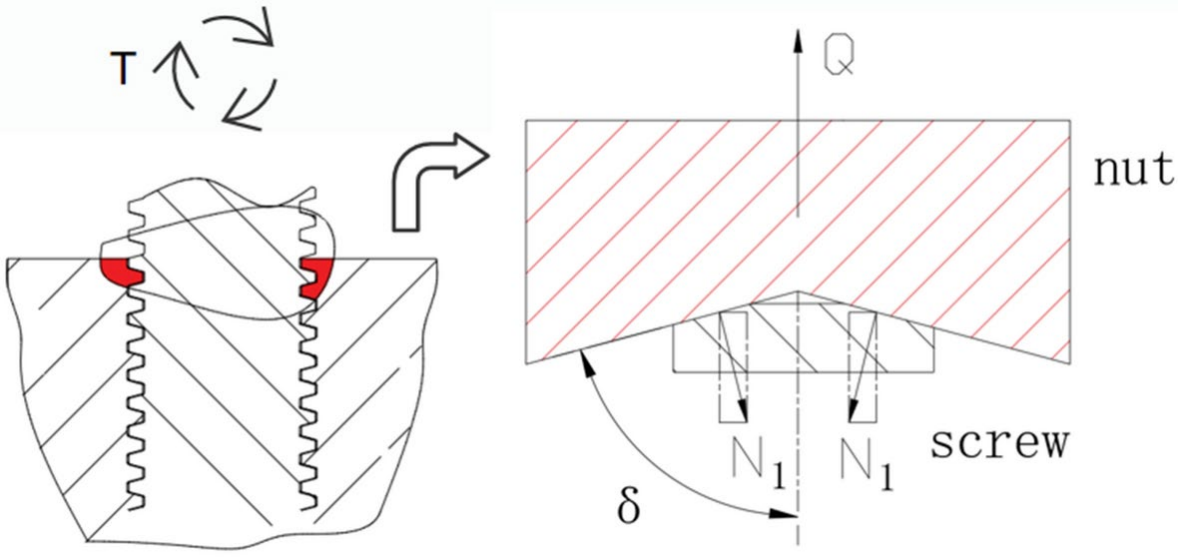
Spiral motion force cross-section.

Obviously derived, it can be used to use the inverse trigonometric function to find $\mu$. In order to facilitate the equivalent coefficient of friction, this nut-screw helical motion forces can be simplified into the form of the Fig. 3: the nut is supported by the screw vertically upward axial force Q, the screw is subjected to pressure on both sides of the beveled surfaces, by the torque of the force of the T pulling in the wedge groove of the rotary motion:

The analysis of the balance of vertical force and frictional force is as follows:

4 $$P = 2 * N_{1} * \mu$$

where μ is the coefficient of friction.

5 $$Q = 2 * N_{1} * \sin \delta$$

It can be derived from Eqs. 4 and 5:

6 $$P = Q * \frac{{\mu_{{}} }}{\sin \delta }$$

The equivalent coefficient of friction can then be obtained:

7 $$\mu_{1} = \frac{\mu }{\sin \delta }$$

The self-locking condition can be further deduced from the above condition:$\mu_{1} \ge \tan \lambda$ evolves as:

8 $$\mu \ge \frac{kt*\sin \delta }{{\pi d_{2} }}$$

### Axial force analysis of trapezoidal threads

**Fig. 4:**
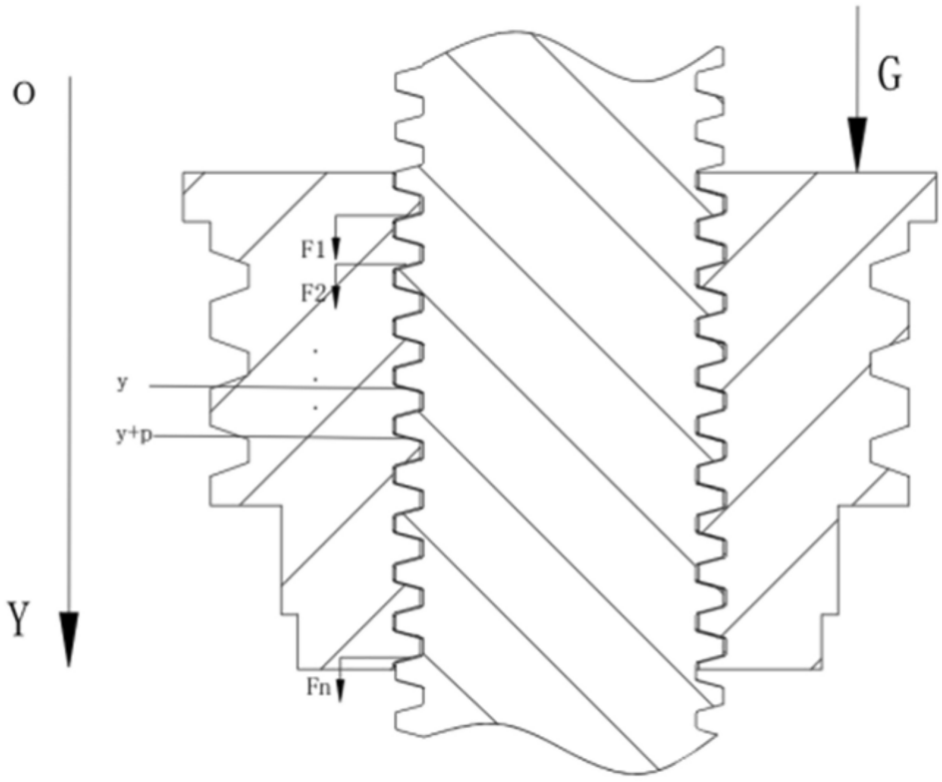
Axial force diagram of trapezoidal thread teeth.

Under the load force G, axial force^21^ F is generated on the contact shoulder surfaces of the inner and outer threads of the structure, and the force is shown in Fig. 4.

Taking the axis of the small end of the female threaded cone as the origin, establish the one-dimensional coordinates $OY$, the axial force is a function of the coordinates $y$. Let the axial load on the cross-section of $x$ within the screwing length of the thread be $F(y)$, and the distribution of the load between the turns of the thread $f(y)$ is the derivative with respect to $y$, then:

9 $$f\left( y \right) = \frac{dF\left( y \right)}{{dy}}$$

10 $$F\left( y \right) = \int\limits_{0}^{y} {f(y)} dy$$

Take $i$ as an integer to indicate the numbering of the entire turn of the thread starting from the small end of the thread:

11 $$f_{1} = F\left[ {n - (i - 1)P} \right] - F(n - iP) = \int\limits_{n - iP}^{n - (i - 1)P} {f(y)dy} \;\;(i = 1,2,3, \ldots ,n)$$

where:$n$ is the number of complete pitches in the threaded length.

The load on any threaded tooth can be calculated by finding F(x) or f(x).

### Theory of orthogonal experiments

Orthogonal experiment is an experimental design method^22^. Its core idea is to find the optimal experimental program by selecting representative test points, utilizing the principle of orthogonality, and comprehensively studying the effects of multiple influencing factors on the experimental indexes under a smaller number of tests. The basic principle is: the variables in the experiment are split into several independent parts, and then determine the values of each part, so as to obtain a set of parameter combinations that are orthogonal to each parameter variable in the model. This design makes the influence of each factor on the experimental results can be clearly reflected, the effective control of the experiment, so as to control the experimental results. Orthogonal experimental design is widely used in drug research and development, product design, process optimization, market research, chemical field^23^ and other fields. Overall, orthogonal experimental theory is an efficient, fast and economical experimental design method, which can comprehensively study the influence of multiple levels on experimental indexes, and provide strong support for research in various fields with fewer trials.

## Standard modeling and meshing

**Fig. 5:**
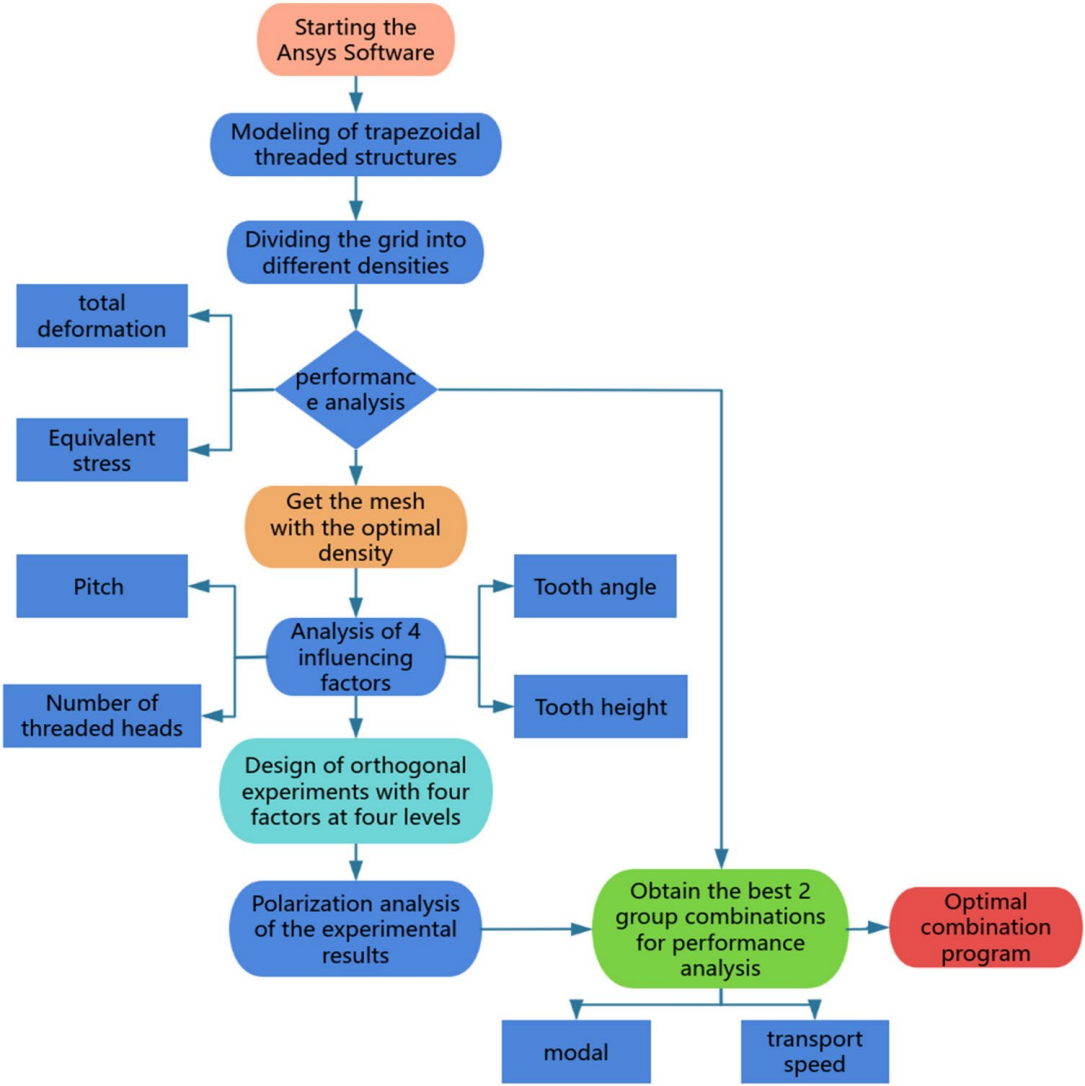
Analyze the flowchart.

During the optimization design process, the trapezoidal thread structure is not only affected by the common target parameters of equivalent force and total deformation, but also by the target factors of transmission efficiency and transmission frequency when doing helical transmission. These target parameters are affected by the factors tooth angle A, tooth height H, pitch P and the number of thread heads N. The simulation and analysis flowchart of this paper is shown in Fig. 5.

### Screw nut modeling

**Fig. 6:**
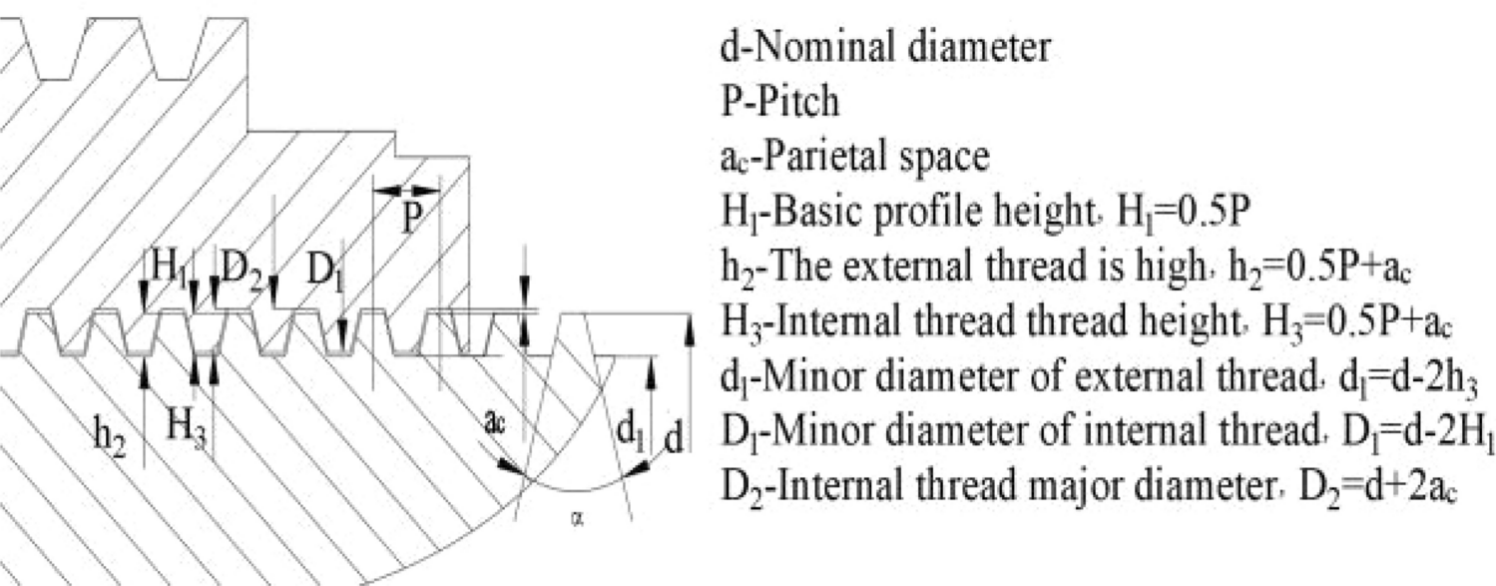
The main dimensions and relationship diagram of trapezoidal threads.

Digital structural modeling and simulation is a very important technical tool in today’s engineering field. Through digital modeling, engineers can accurately describe and predict the various behaviors of structures for better design and optimization. Referring to the structural dimensions of trapezoidal threads in a company’s lifting equipment, it is based on mechanical design manuals and design standards for the main dimensions of the trapezoidal thread cross-section geometry^24–26^, and the relative constraints of its various parameters are shown in Fig. 6.

3D modeling of trapezoidal threaded structures can be easily achieved with Solidworks software^27^.Solidworks software can be used to model the trapezoidal thread structure easily. After assembling the three-dimensional drawing of the screw and nut structure, the Solidworks software and Ansys Workbench software are associated. The associated Solidworks software can directly open the Ansys Workbench software, which makes the modeling process efficient and accurate, and provides a solid foundation for the subsequent simulation and analysis.

### Initial boundary condition setting

The LS-Dyna module of Ansys Workbench software is used to analyze the dynamics of the trapezoidal thread structure of the screw nut. According to the standard trapezoidal thread in the sixth edition of the mechanical design manual as the initial size, set the number of thread heads as the conventional single-head thread, and its geometric parameters are shown in Table 1.

**Table 1:** Main parameters of trapezoidal thread.

Nominal diameter d/(mm)	Pitch P/mm	Threaded head N/pcs	Tooth angle A/°	Tooth height H/mm
60	9	1	30	5

### Simulation analysis of screw nut mesh convergence

Open Ansys Workbench software in the associated Solidworks software toolbar and open the LS-Dyna Analysis Module to share the geometry. The material of the screw nut for the analysis module is commonly used structural steel, and its main parameters are shown in Table 2.

**Table 2:** Main parameters of materials.

Material	Density/(g/cm^3^)	Poisson’s ratio	Elastic modulus/GPa	Allowable stress/MPa
Structural steel	7.85	0.3	200	300

After the material parameters are set in ANSYS data, the next meshing step is carried out. Trapezoidal threads, as a special geometric shape of thread type, have a complex structure, and the general delineation method can not be well adapted to its shape characteristics while guaranteeing the quality of mesh delineation. However, tetrahedral cells can be flexibly adapted to complex geometries, improve efficiency through optimization algorithms, accurately simulate the stress distribution, capture subtle changes, and facilitate the processing of boundary conditions and the simulation of the actual stress state. Therefore, it is more appropriate to use tetrahedral cells for meshing to import the screw nut model.

The choice of mesh size needs to be weighed according to specific engineering applications and computational requirements. Generally speaking, too small a mesh will lead to a sharp increase in computation, thus requiring more computational resources and time to complete the simulation. ANSYS LS-Dyna is well known for its nonlinear analysis, efficient solver, multiphysics field coupling, flexible modeling, and highly efficient computational capability in dynamics simulation, which is able to accurately simulate the dynamic response of complex structures, and assist in the optimization of engineering. With 6000N nut load and remote displacement applied to the trapezoidal threaded structure, the total deformation and equivalent stresses under eight mesh sizes were counted, and the results are shown in Table 3.

**Table 3:** The main parameters of different mesh densities.

Model scenarios	S1	S2	S3	S4	S5	S6	S7	S8
Unit size	21	18	16	14	12	10	8	6
Node	8382	10,193	11,384	13,930	17,636	24,026	36,332	67,301
Number of units	29,361	35,787	39,875	51,410	68,498	98,484	161,993	309,748
Total deformation/um	7.407	8.070	4.655	2.285	2.080	1.424	0.749	1.666
Equivalent stress	19.175	24.107	17.939	4.457	4.609	3.915	2.394	5.054

**Fig. 7:**
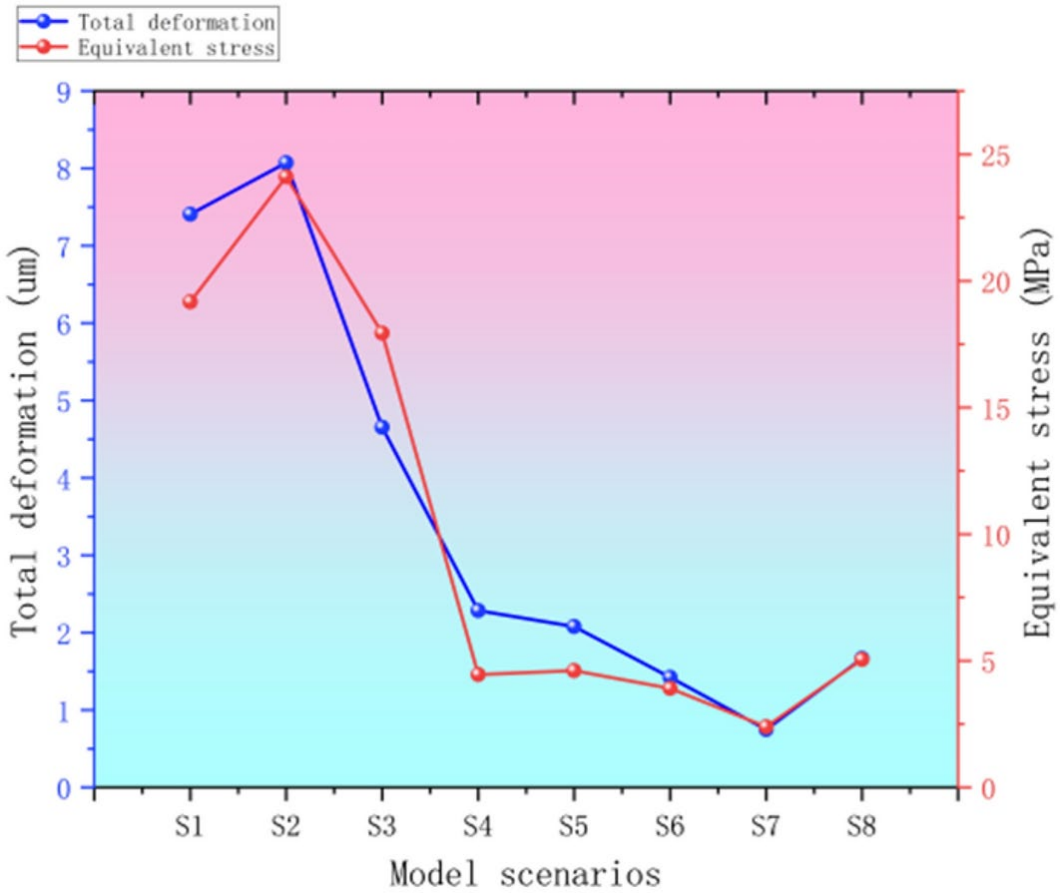
Parameter charts for different mesh densities.

The total deformation and equivalent stress parameter data of the simulation results in Table 3 are counted as easy-to-view graphs to determine the optimal meshing scheme, as shown in Fig. 7.

From observing the figure, it is evident that as the grid cell size decreases, the simulation data transitions from divergence to gradual convergence. However, as the grid cell size continues to diminish, it begins to revert back to a divergent state.

## Optimized design based on orthogonal experiments

During the optimization design process, the trapezoidal thread structure is not only affected by the common target parameters of equivalent force and total deformation, but also by the target factors of transmission efficiency and transmission frequency when doing helical transmission^28^. These target parameters are all affected by the factor pitch. These target parameters are all affected by the factors, tooth angle A, tooth height H,pitch P and number of thread heads N.

### Trapezoidal thread pitch P-factor analysis

The pitch P of trapezoidal thread, as a core parameter in thread design, has an important influence on the multifaceted characteristics of the thread mechanism^29^. It is not only related to the movement performance and speed of the thread, but also directly determines the load carrying capacity and strength of the thread. In mechanical design and manufacturing, the selection of pitch P is particularly critical and requires comprehensive consideration of multiple factors such as application scenarios, load conditions and performance requirements. Pitch P is particularly critical in mechanical design and manufacturing^30^.

**Fig. 8:**
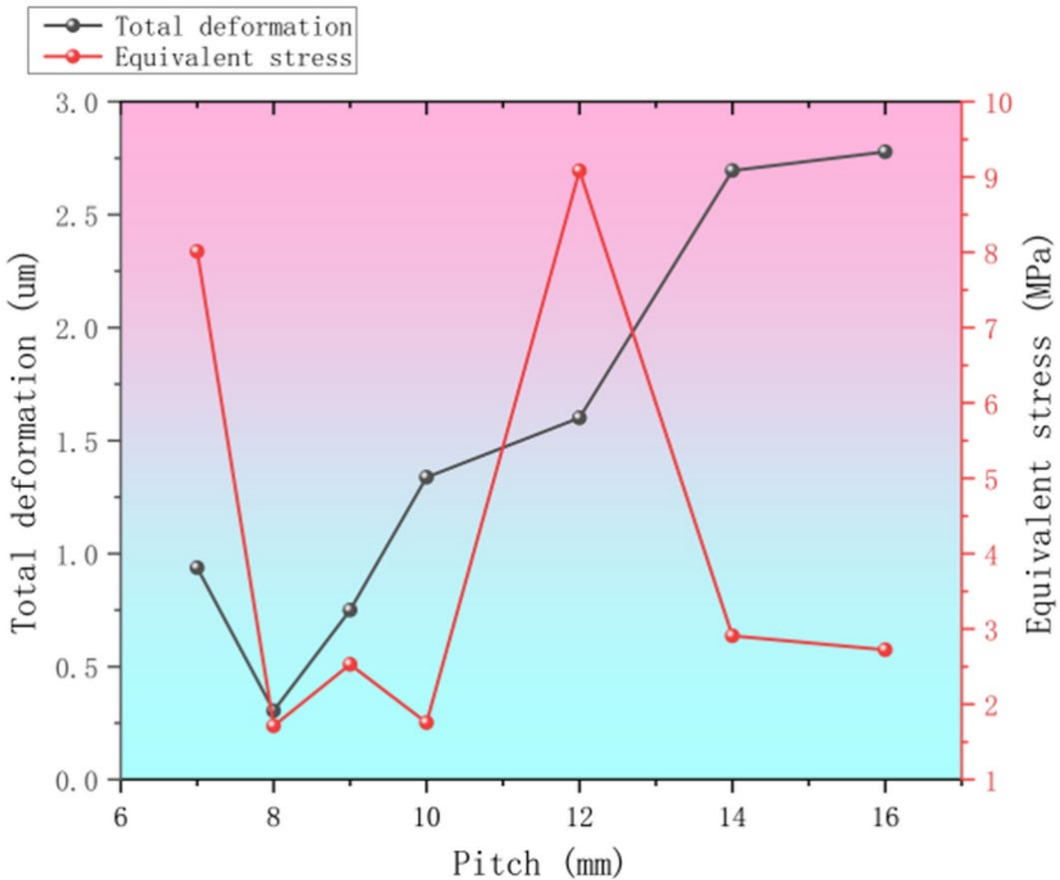
Statistical chart of target parameters at different pitches.

By changing the size of the pitch P, other parameters qualify the relationship unchanged can be obtained from different models, these models were simulated under the same conditions and mesh density, the total deformation, equivalent force parameters obtained after finishing statistics as shown in Fig. 8.

### Trapezoidal thread head number factor analysis

Multi-thread and single-thread threads are widely used in mechanical engineering, but the differences between the two in terms of key parameters and application characteristics are obvious. Multi-threaded due to a number of grooves, so the lead is larger, the screwing speed is correspondingly faster, which is extremely favorable for the need for rapid interface occasions, or the need to change the speed of the power to be transmitted out. At the same time, the force distribution of the multi-head thread is more uniform, and can withstand larger loads. However, due to the increased lead, the self-locking ability of the multi-head thread is relatively weak, and it needs to be carefully selected when strong self-locking ability is required^30^. The threads can be selected carefully when strong self-locking ability is required.

In contrast, single-ended threads have only one helical groove with a lead equal to the pitch, which makes them simpler to manufacture and more flexible to fit. In addition, the single head thread has a small helical rise angle and therefore has a strong self-locking capability, which makes it particularly suitable for applications where locking is required. However, its relatively slow screwing speed may not be suitable for scenarios with high speed requirements.

**Fig. 9:**
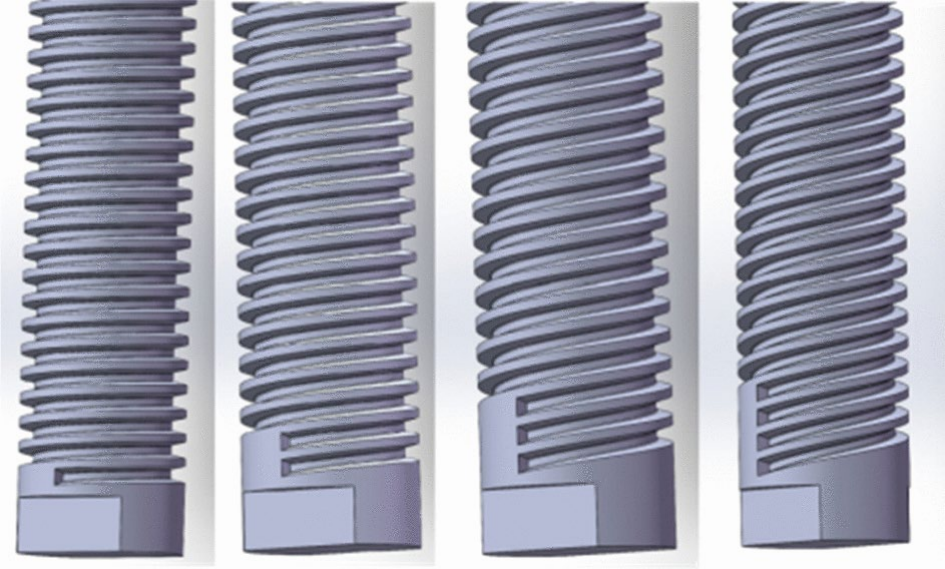
Comparison of different thread head numbers.

In this paper, the pitch P is 9 boundary condition and the number of thread heads are 1, 2, 3 and 4 as variables for dynamic simulation analysis. Different thread head numbers have corresponding thread line numbers, respectively, and the three-dimensional visualization is shown in Fig. 9.

**Fig. 10:**
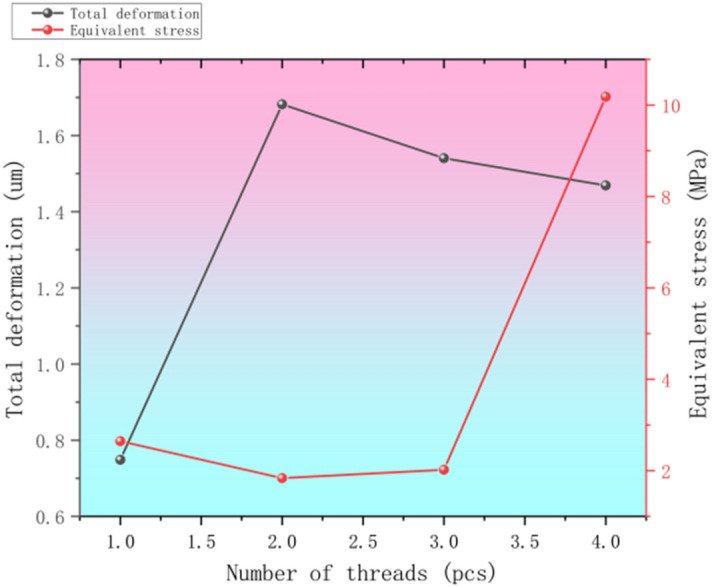
Statistics of target parameters with different number of thread heads.

After the software set the same working conditions and grid density cell size, the total deformation and equivalent stress results can be obtained after simulation respectively as shown in Fig. 10.

### Trapezoidal thread cross-section angle factor effects

The cross-section angle of trapezoidal thread has a significant effect on its helical drive, total deformation and equivalent force under the condition that the pitch remains unchanged, because the change of this angle is directly related to the contact area of the thread, the distribution of force and the angle of force. When the section angle increases, the contact area of the thread side also increases, and this increased contact area can effectively improve the contact stiffness, thus reducing the deformation when bearing the same load. At the same time, a larger section angle can make the force more evenly distributed between the bolt and the nut, which helps to reduce the stress concentration and reduce the total deformation. In addition, the increased angle also makes the transmitted force tend to be perpendicular to the contact surface of the threads, reducing the shear stresses caused by the inclined direction of the force, which mainly generates compressive stresses. In terms of equivalent stresses, a larger section angle helps to reduce the level of equivalent stresses in the thread when subjected to force by increasing the contact area to disperse the stresses and reduce stress concentrations^31^. The same applies to the stresses in the threads. On the contrary, smaller section angles may lead to higher stress concentrations and shear stresses due to the small contact area, increasing the deformation of the thread and the potential risk of fatigue damage. Therefore, the selection of a suitable cross-section angle is essential to optimize the mechanical properties of threads, especially when designing threaded connection systems that require high load carrying capacity, excellent fatigue resistance and good fitment.

**Fig. 11:**
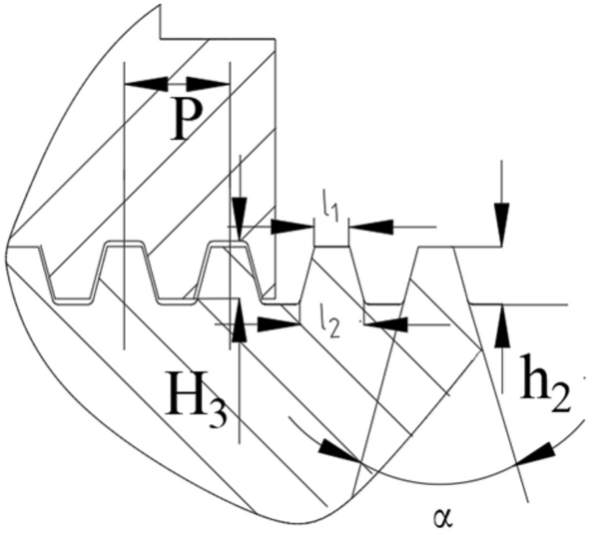
Schematic diagram of trapezoidal thread dimensions.

Therefore, the design needs to make reasonable trade-offs and choices based on the consideration of various factors to ensure that the drive system can work stably and reliably in the process of use. Advanced tools such as finite element analysis^32^ need to be used to assess the specific impact of different angles on the performance of the thread to ensure the optimization of the design and the reliability of the actual application. Ensure that the angle is changed under the condition that the pitch and other factors remain unchanged, as shown in Fig. 11.

Taking the pitch P = 9 as a boundary condition, the dimensional relationship associated with the trapezoidal thread section is obtained as:

12 $$\left\{ \begin{gathered} l_{1} + l_{2} = 9 \hfill \\ H_{3} = h_{2} = 5 \hfill \\ \alpha = [20^\circ ,25^\circ ,30^\circ ,35^\circ ,40^\circ ] \hfill \\ \end{gathered} \right.$$

where $l_{1}$ is the apex width,$l_{2}$ is the root width, and the rest of the parameters are defined with reference to Fig. 6.

**Fig. 12:**
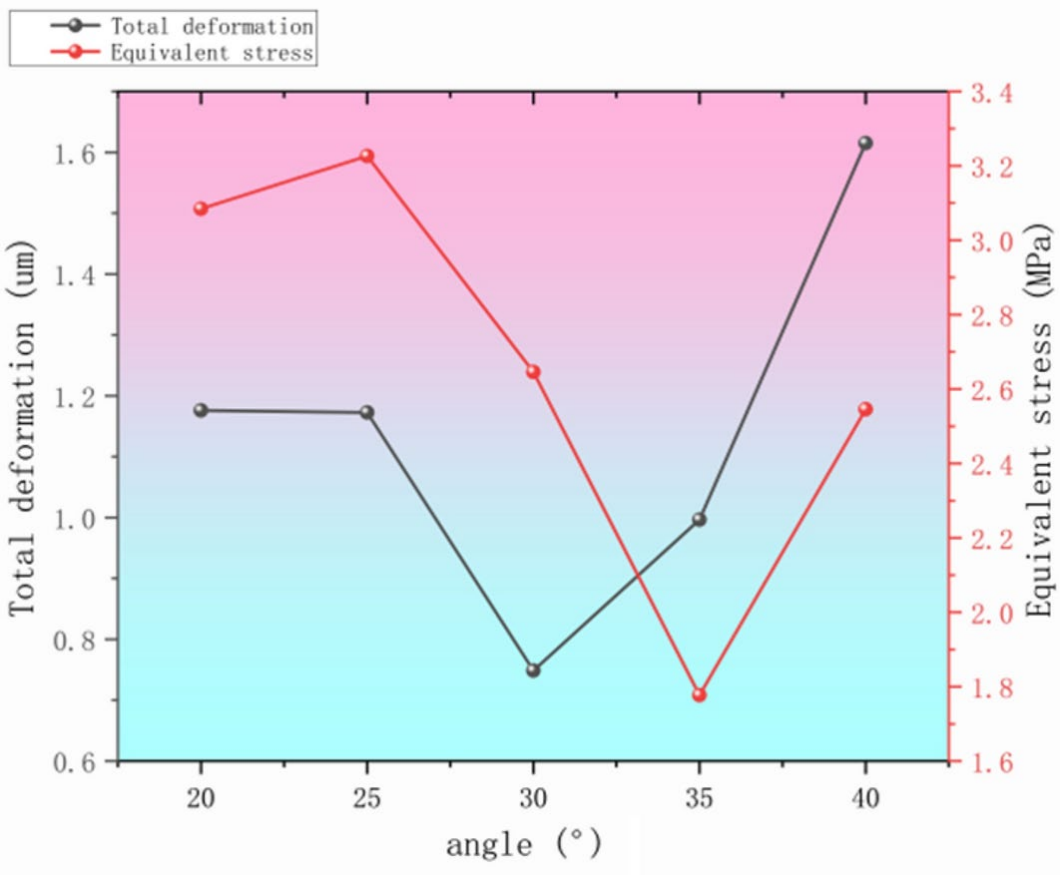
Statistics of target parameters at different thread angles.

Through the size relationship using SOLIDWORKS software to draw five different angle size model, respectively, through the ANSYS Workbench in the LS-Dyna module for the same mesh method density and working conditions set conditions for the following simulation, can be obtained in the display of the total deformation under the dynamics of the equivalent force data, collated statistics as shown in Fig. 12.

### Trapezoidal thread section tooth height factor influences

**Fig. 13:**
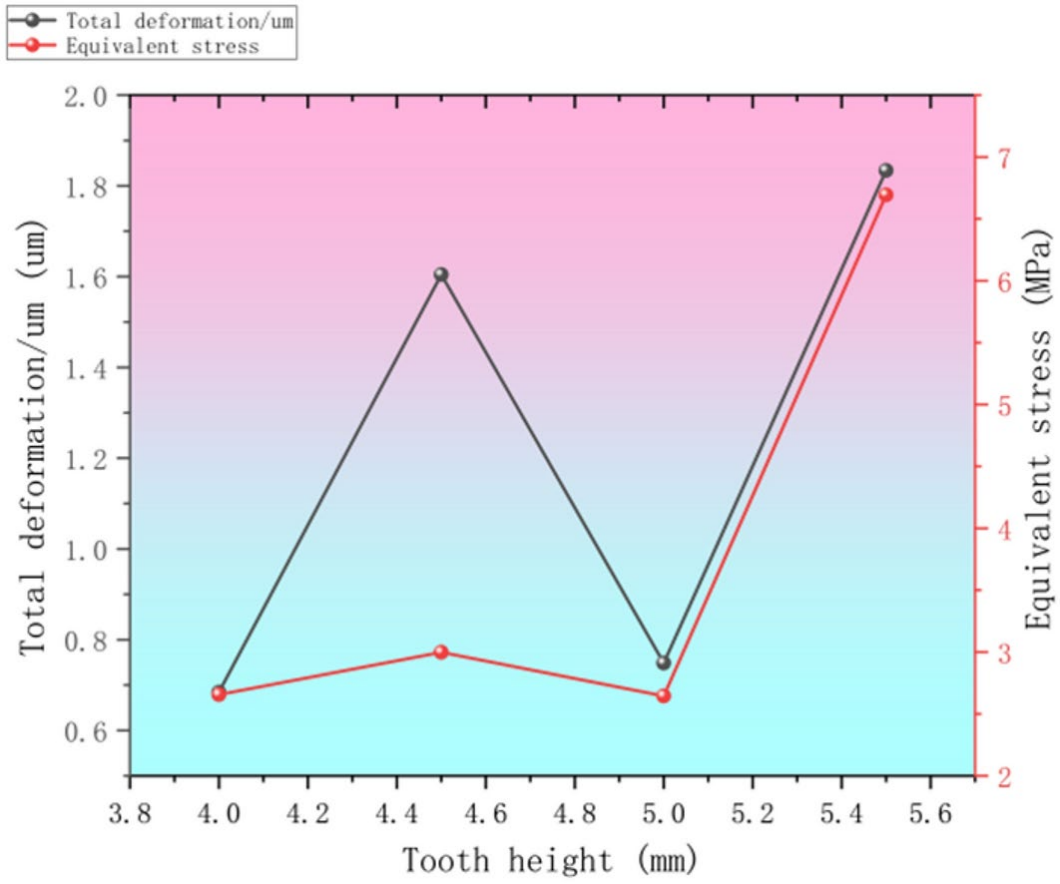
Statistics of target parameters at different thread heights.

The tooth height of trapezoidal threads has an important influence on their total deformation, equivalent stress and overall dynamics^33^. A higher tooth height means a larger contact area in the thread contact area, which helps to distribute the load between the bolt and the nut more evenly, thus reducing local deformation and stress concentration, and increasing the overall stiffness of the joint^34^. The increased contact area not only reduces the load between the bolt and nut, but also increases the overall stiffness of the connection. This increased contact area not only reduces the equivalent stresses, which extends the fatigue life of the threads, but also improves self-locking properties, as the increased frictional resistance helps to prevent the threads from loosening automatically under vibration or repeated loading conditions. However, a higher tooth height also means that more torque is required during assembly and disassembly, which can increase operational complexity and tooling requirements. In terms of wear and longevity, greater tooth height reduces the wear rate of individual teeth by spreading the load, especially in high load and high wear applications. In addition, higher tooth heights provide better damping, helping to reduce noise and potential damage from vibration and improve dynamic response. Thread design optimization was carried out using finite element analysis by setting all other parameters constant, designing different tooth height models, simulating them and then analyzing their effect on the thread performance, the total deformation and equivalent force parameter statistics are shown in Fig. 13.

## Comprehensive analysis of the results of orthogonal experiments

### Orthogonal design of experiments

From the above study and analysis, it is known that the total deformation (ΔL) and the equivalent force S in the re-screwing process of trapezoidal threads are related to the pitch P, the number of thread heads N, the angle of trapezoidal teeth A, and the height of threads H. The trapezoidal thread model can be further optimized to achieve a better working condition. In order to achieve a better working condition of the trapezoidal thread structure, the thread model needs to be further optimized. By arranging and analyzing multi-factor tests through orthogonal experimental design and analyzing the results of some representative tests, we can understand the situation of all factor tests and then find out the optimal combination of each factor level^35^. The best combination of factor levels can be found. Here, orthogonal tests with good target parameters for each of the four factors mentioned above are used to study the effects of the above factors on the total deformation and equivalent stress of the trapezoidal thread structure, and the designed orthogonal tests and results are shown in Table 4.

**Table 4:** Orthogonal experimental design point calculation results.

Weave number	Factor				Impact results	
	P/mm	N/pcs	A/°	H/mm	ΔL/μm	S/MPa
1	8	1	25	4	0.44331	2.5543
2	8	2	30	4.5	1.7727	4.671
3	8	3	35	5	1.8856	2.7495
4	8	4	40	5.5	5.0697	14.974
5	9	1	30	5	0.74856	2.3918
6	9	2	25	5.5	2.3375	2.936
7	9	3	40	4	0.39034	3.9517
8	9	4	35	4.5	4.2667	15.645
9	10	1	35	5.5	2.3356	2.9363
10	10	2	40	5	2.14	2.8028
11	10	3	25	4.5	1.1652	3.7367
12	10	4	30	4	3.5523	4.0296
13	12	1	40	4.5	4.5186	2.0413
14	12	2	35	4	4.396	15.344
15	12	3	30	5.5	3.323	4.0417
16	12	4	25	5	3.9691	5.915

### Analysis of the total deformation and equivalent stress range of trapezoidal threads

**Fig. 14:**
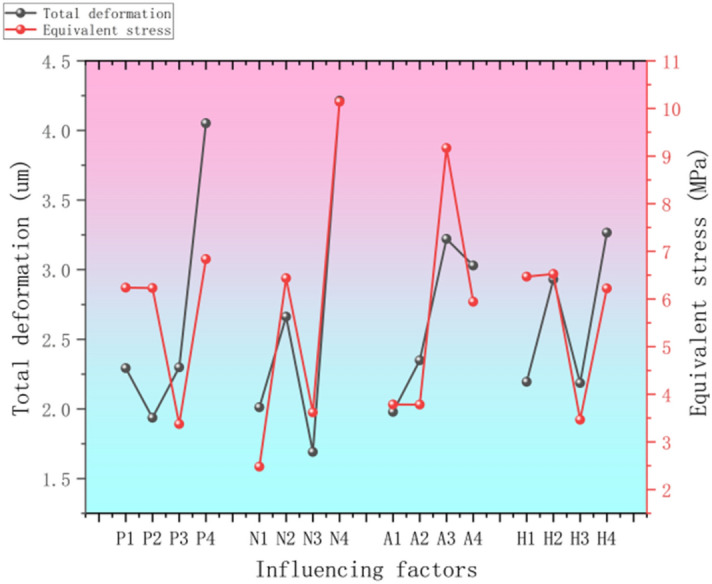
Statistical plot of mean values of target parameters under different factors.

In the orthogonal test, in order to evaluate the influence of each factor on the total deformation or equivalent force of trapezoidal threads, and to obtain the parameter combinations that minimize the total deformation and the most equivalent force in the helical motion of the trapezoidal thread structure, the results of the orthogonal test were analyzed by using the polar analysis method in Table 4. After the calculation of polar analysis, the average values of total deformation and equivalent force of trapezoidal threads were obtained under the levels of different factors, and the data curves were plotted as shown in Fig. 14 after categorization and organization, and the larger the corresponding polar range of the test factors, the larger the influence of the factors on the total deformation or equivalent force of trapezoidal thread structure^36^.

From the average value of the black curve in Fig. 13, it can be obtained that the range of the influence of factors P, N, A, and H on the total deformation of the trapezoidal thread structure is 2.116, 2.523, 1.051, and 1.08, respectively, so the importance of these four factors to the total deformation of the trapezoidal thread structure is ranked as N > P > H > A. For the proposed trapezoidal thread structure, the minimum parameter combination of the total deformation of the trapezoidal thread structure is P_2_N_3_A_1_H_3_. The range of the influence of the average data factors P, N, A, and H on the total deformation of the trapezoidal thread structure in Fig. 14 is 3.46, 7.66, 5.385, and 3.059, respectively, so the importance of these four factors to the total deformation and equivalent stress of the trapezoidal thread structure is ranked N > A > P > H. Therefore, for the proposed trapezoidal thread structure, the minimum equivalent stress of the trapezoidal thread structure is P_3_N_1_A_3_H_3_.

### Analysis of orthogonal optimization results

**Fig. 15:**
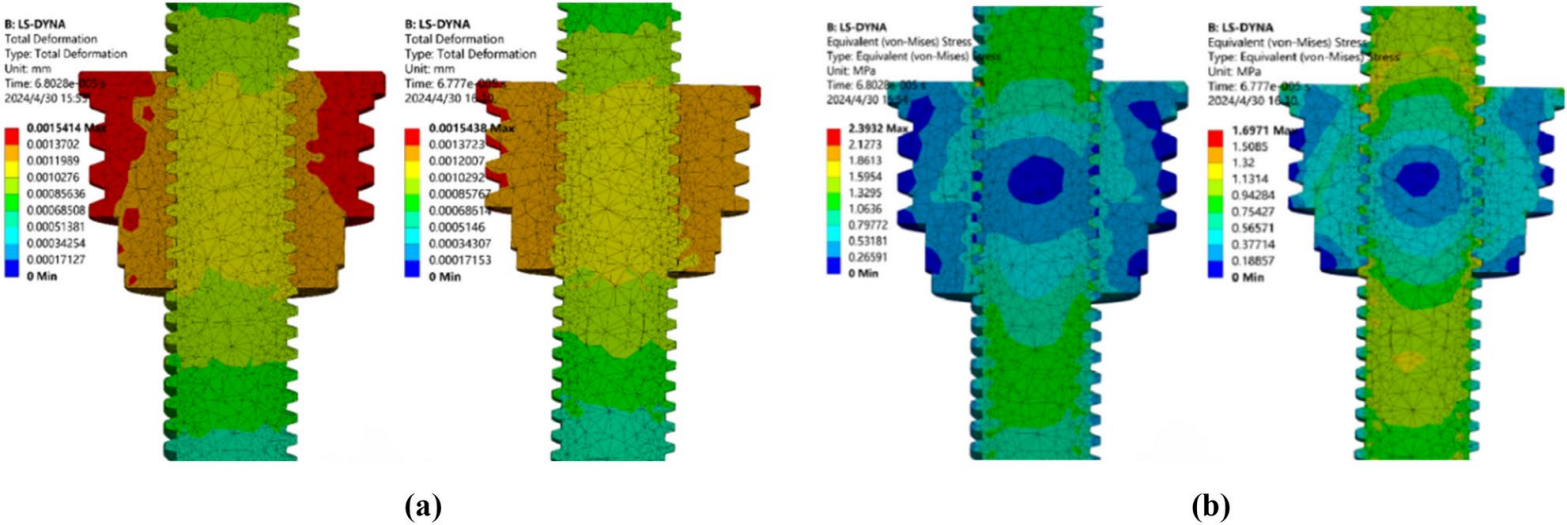
Cloud of target parameters of the optimization scheme. (**a**) Optimize the total deformation contour of the scheme (**b**) Equivalent force contours of the optimization scheme.

Optimization design process, in the pursuit of reducing the total deformation at the same time, but also the pursuit of the minimum equivalent force is a multi-objective optimization problem^37^. The optimization results often conflict with each other and cannot reach the optimal solution at the same time, so it is necessary to consider them in a balanced way and finally get an equilibrium solution.The numerical modeling of the combination of orthogonal optimization scheme P2N3A1H3 and P3N1A3H3 under the same working condition setting and mesh density division is shown in Fig. 15: when the parameter combination is P_2_N_3_A_1_H_3_, the total deformation is 1.5414 μm, and the equivalent stress is 2.3932 MPa, and when the parameter combination is P_3_N_1_A_3_H_3_, the total deformation is 1.5438 μm, and the equivalent stress is 1.6971 MPa. Compared with the parameters of the S7 grid in the initial standard scheme as shown in Fig. 9, the total deformation of the two schemes is increased, but the total deformation is very small, which meets the requirements under working conditions. For the equivalent stress, scheme P2N3A1H3 increased by 0.0014 MPa and scheme P3N1A3H3 decreased by 0.6974 MPa.

### Frequency comparison analysis of optimization results

Screw drive system in actual operation will be subject to a variety of frequencies of external excitation, when these excitation frequencies and the system’s intrinsic modal frequency is close to or consistent, it will trigger the resonance phenomenon. Resonance will not only lead to a sharp increase in the amplitude of the transmission system, affecting the stability and accuracy of the transmission, but also may cause serious fatigue damage to the internal components of the system, shortening the service life of the equipment. Therefore, an in-depth study of the modal frequency resonance phenomenon of the trapezoidal thread screw drive system is of great significance to ensure the smooth operation of the drive system, improve the transmission efficiency and extend the life of the equipment^38,39^. By optimizing the design to avoid resonance phenomenon, vibration and noise can be reduced to ensure the efficient and stable operation of the screw drive system and prolong its service life, and at the same time, improve the working environment to protect the health and safety of the operators.

**Fig. 16:**
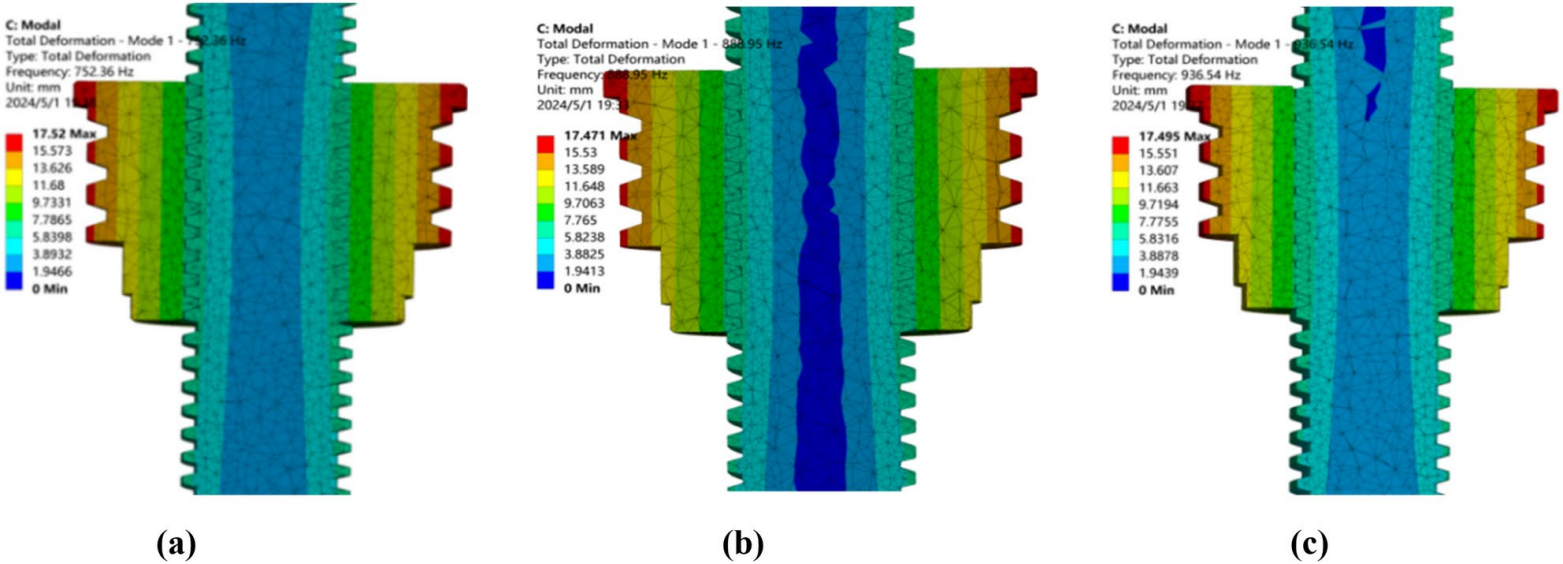
Cloud plot of 1st order modal frequency comparison. (**a**) Pre-optimization modal cloud (**b**) Optimization scheme P_2_N_3_A_1_H_3_ modal cloud (**c**) Optimization scheme P_3_N_1_A_3_H_3_ modal cloud

Through the modal simulation of the three combined schemes before and after the optimization of the 1st order frequency cloud results are shown in Fig. 16, through the comparison of cloud diagrams can be seen that the 1st order frequency of orthogonal optimization have been improved to a certain extent, and the operating frequency range has been further expanded.

### Comparative analysis of load rates of optimization results

Trapezoidal thread construction in screw drives is affected by a number of factors, including the rotational speed of the screw, the pitch of the screw, the lead of the thread, and the efficiency of the drive. Among them, the pitch and lead of the screw determine the distance the nut can move along the axis at a given rotational speed, which in turn affects the rate of transportation. Since the scheme P2N3A1H3 is a multi-head thread, the lead is the largest, and the carrying rate is the largest under the same speed, followed by the scheme P3N1A3H3, and the original scheme is the smallest.

### Optimal program selectionoptimal program selection

After the orthogonal analysis is completed, the obtained two sets of schemes can be further optimized and analyzed in combination with the optimization objectives of modal frequency^40–42^ and carrying rate. The design candidates for the three groups of trapezoidal threads of the original standard parts scheme combination, the P_2_N_3_A_1_H_3_ scheme and the P_3_N_1_A_3_H_3_ scheme are recorded as schemes 1, 2 and 3, as shown in Table 5.

**Table 5:** Comparison table of trapezoidal thread combination schemes.

Parameter name	Option 1	Option 2	Option 3
P/mm	9	9	10
N/mm	1	3	1
A/°	30	25	35
H/mm	5	5	5
Total deformation/μm	0.749	1.541	1.544
Equivalent stress/MPa	2.392	2.393	1.697
Frequency/Hz	752.36	888.95	936.54
Lead/mm	9	27	10

Considering the very small deformation of the three groups of schemes, the conditions of normal transportation rate and the design consideration of the difficulty of multi-head thread processing of scheme 2, scheme 3 is selected as the best design scheme. Through the comparison of parameters before and after optimization, it can be known that only the deformation amount is not optimized in the optimization results of multi-objective, and the rest of the parameters are improved to some extent. Among them, the equivalent force is reduced by 29.1%, the 1st order modal frequency is increased by 24.5%, and the transportation rate is increased by 11.1%.

## Conclusion

Based on the thread mechanism in a certain model of lifting equipment product of an elevator company as the research object, multi-factor analysis and multi-objective optimization are carried out on the structural parameters of the trapezoidal thread structure using standard trapezoidal threads.

1. ANSYS Workbench software was used to analyze the forces and theoretically explore the self-locking conditions of the trapezoidal thread structure in a helical drive. The dynamics simulation was executed through the LS-Dyna module integrated in the software, and the effects of different mesh densities on the results were evaluated. After comparative analysis, the target parameters (total deformation and equivalent force) show the best convergence when the cell size is set to 8 mm.
2. Based on the optimal meshing density determined in conclusion (1), we kept other working conditions consistent and conducted an in-depth multifactorial analytical study on the key factors affecting the performance of helical transmission of trapezoidal threaded structures—pitch P, number of threaded heads N, trapezoidal tooth angle A and tooth height H. The results are summarized in the following table. To this end, an orthogonal experiment was designed to select the best four level parameter combinations for each of these four factors, from which two new sets of design solutions were derived. Subsequently, vibration frequency and transport efficiency were further introduced as evaluation indexes, and a multi-objective optimization comparison was conducted. Ultimately, an optimal design scheme was identified, which resulted in a 29.1% reduction in the equivalent stresses in the target parameters, a 24.5% increase in the first-order modal frequency, and a concomitant 11.1% increase in the transport rate. This result is not only expected to significantly improve the product performance and safety standards of an elevator company, but also provides a useful reference for the performance optimization of screw drive mechanisms in other industries and fields.

Our study emphasizes that the multifactorial analysis of pitch P, number of thread heads N, trapezoidal tooth angle A and tooth height H for the study is based on relatively independent conditions and results in a relatively ideal working condition. Future work needs to investigate the interactions between the factors and validate them with experimental studies in combination with different screw diameter base conditions. Evaluating the mechanical properties in the actual design will further contribute to the design practicality.

## Supplementary Information

Supplementary Information.

## Data Availability

Data is provided within the manuscript or supplementary information files.
