# Supplementary figures and images for: Multifactor exploration and multi-objective optimization of trapezoidal threads
Source: Sci Rep. 2025 Apr 8;15:12020. doi: 10.1038/s41598-025-94144-5 (PMC11978922; doi:10.1038/s41598-025-94144-5)

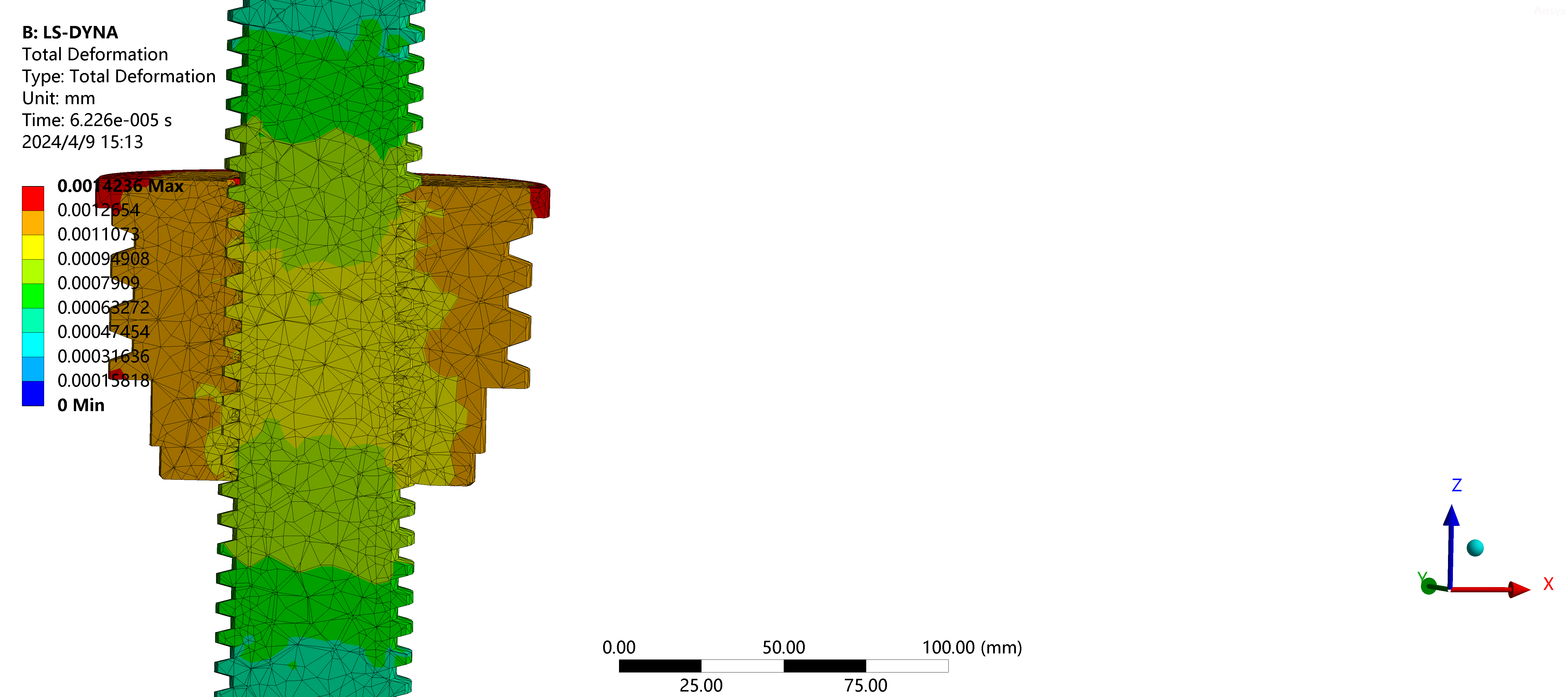

Supplement: Supplementary file 1 — Supplementary Information. [file 41598_2025_94144_MOESM1_ESM.zip › Simulation experiment result graph/Grid division result diagram/变形10.png]

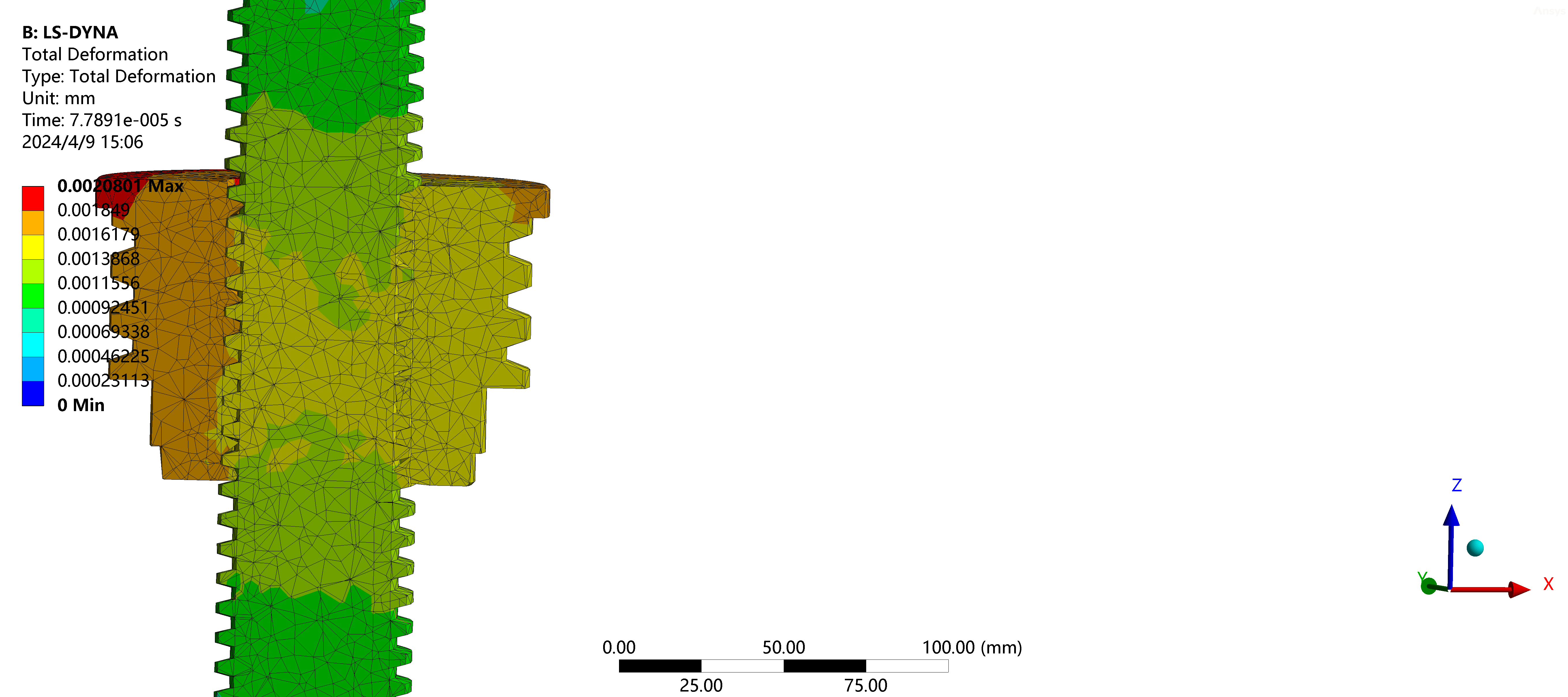

Supplement: Supplementary file 1 — Supplementary Information. [file 41598_2025_94144_MOESM1_ESM.zip › Simulation experiment result graph/Grid division result diagram/变形12.png]

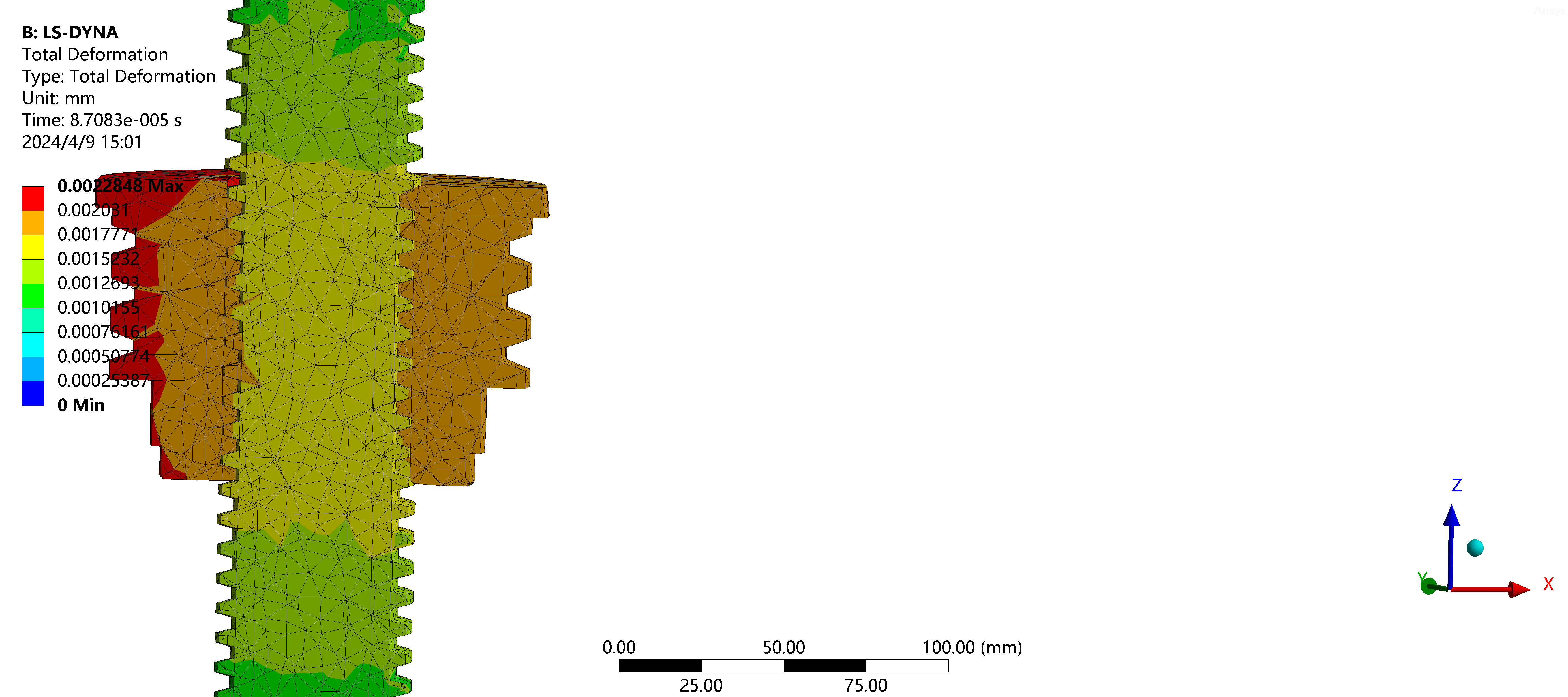

Supplement: Supplementary file 1 — Supplementary Information. [file 41598_2025_94144_MOESM1_ESM.zip › Simulation experiment result graph/Grid division result diagram/变形14.png]

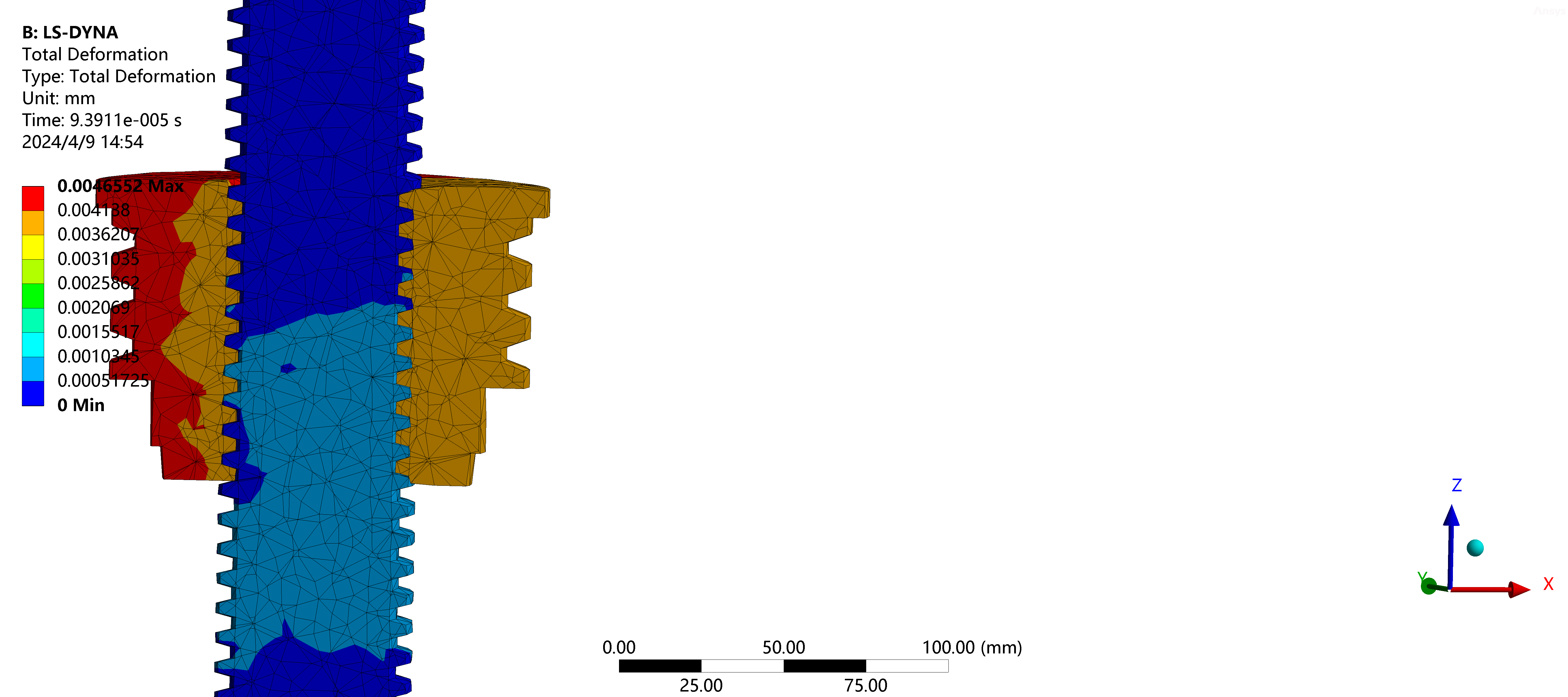

Supplement: Supplementary file 1 — Supplementary Information. [file 41598_2025_94144_MOESM1_ESM.zip › Simulation experiment result graph/Grid division result diagram/变形16.png]

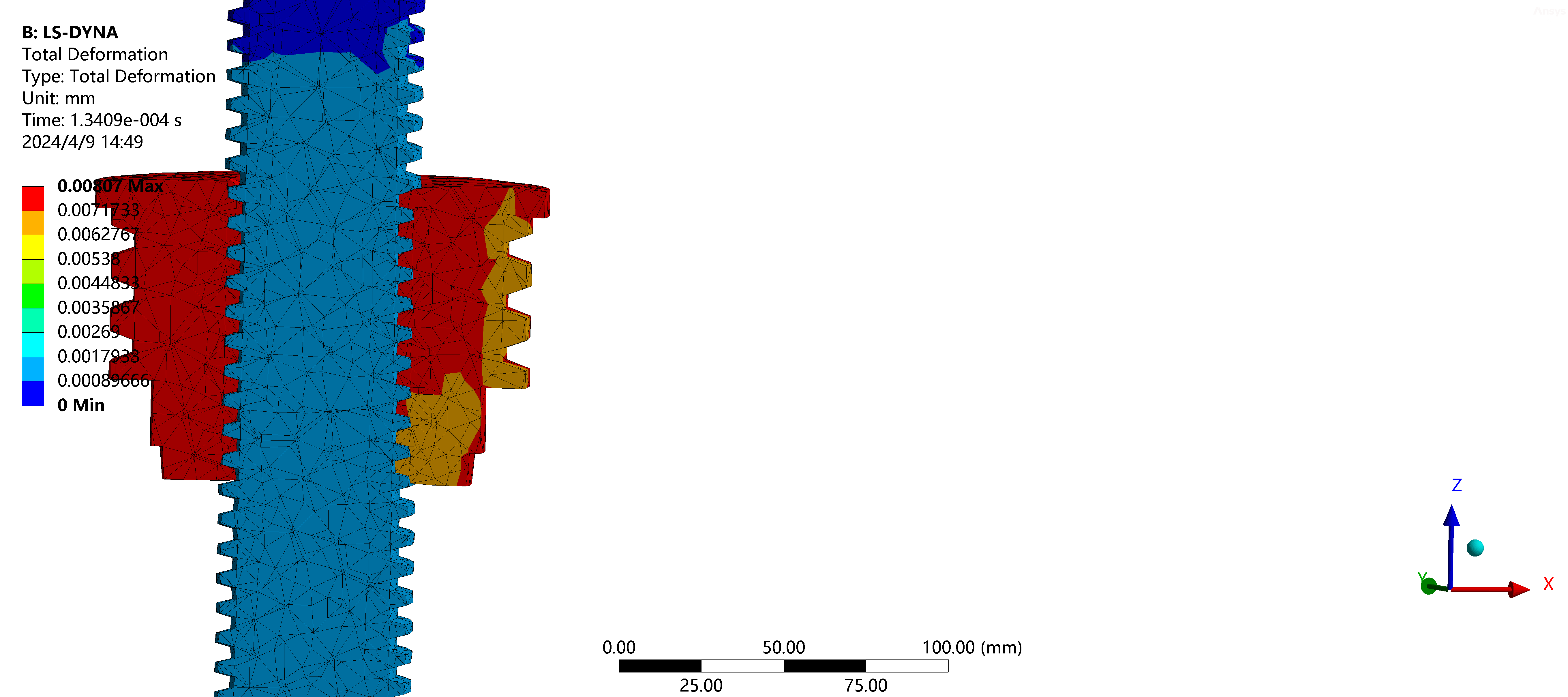

Supplement: Supplementary file 1 — Supplementary Information. [file 41598_2025_94144_MOESM1_ESM.zip › Simulation experiment result graph/Grid division result diagram/变形18.png]

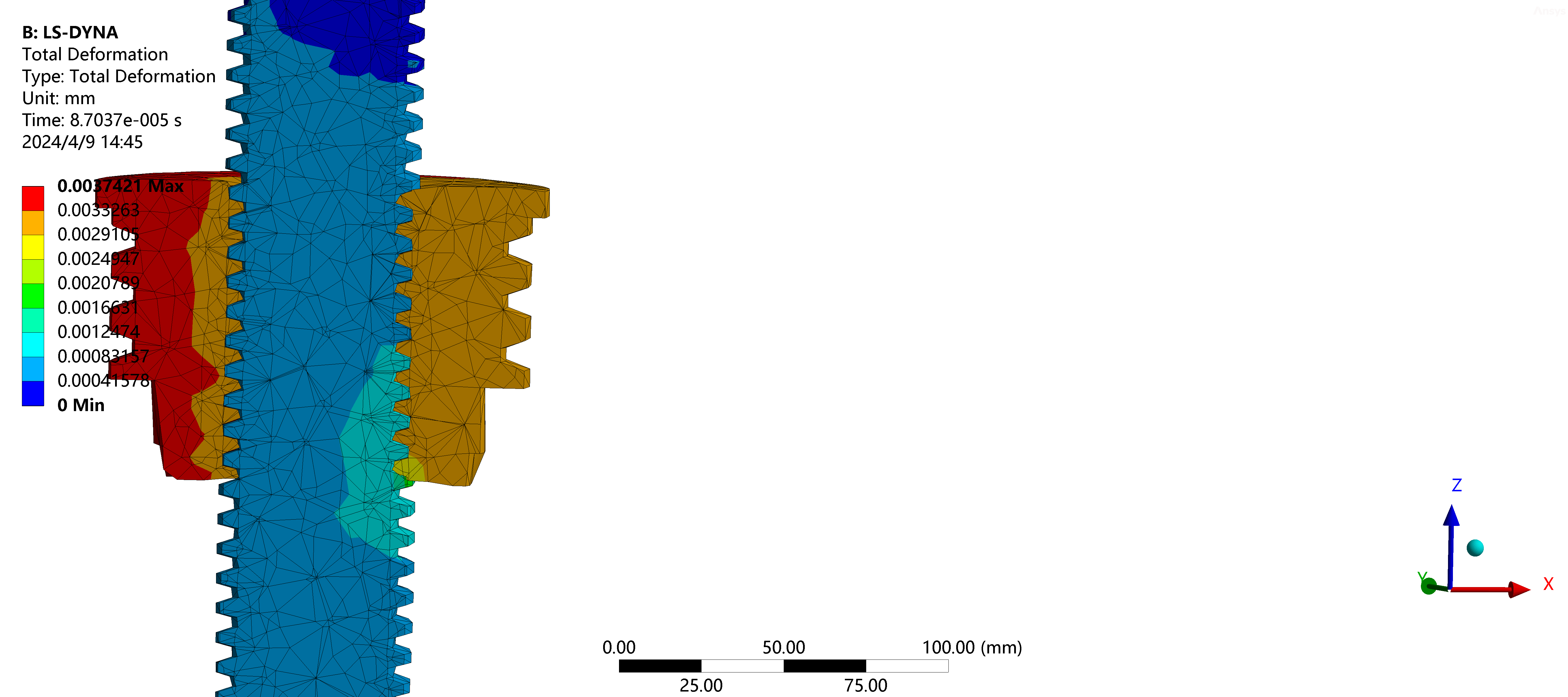

Supplement: Supplementary file 1 — Supplementary Information. [file 41598_2025_94144_MOESM1_ESM.zip › Simulation experiment result graph/Grid division result diagram/变形20.png]

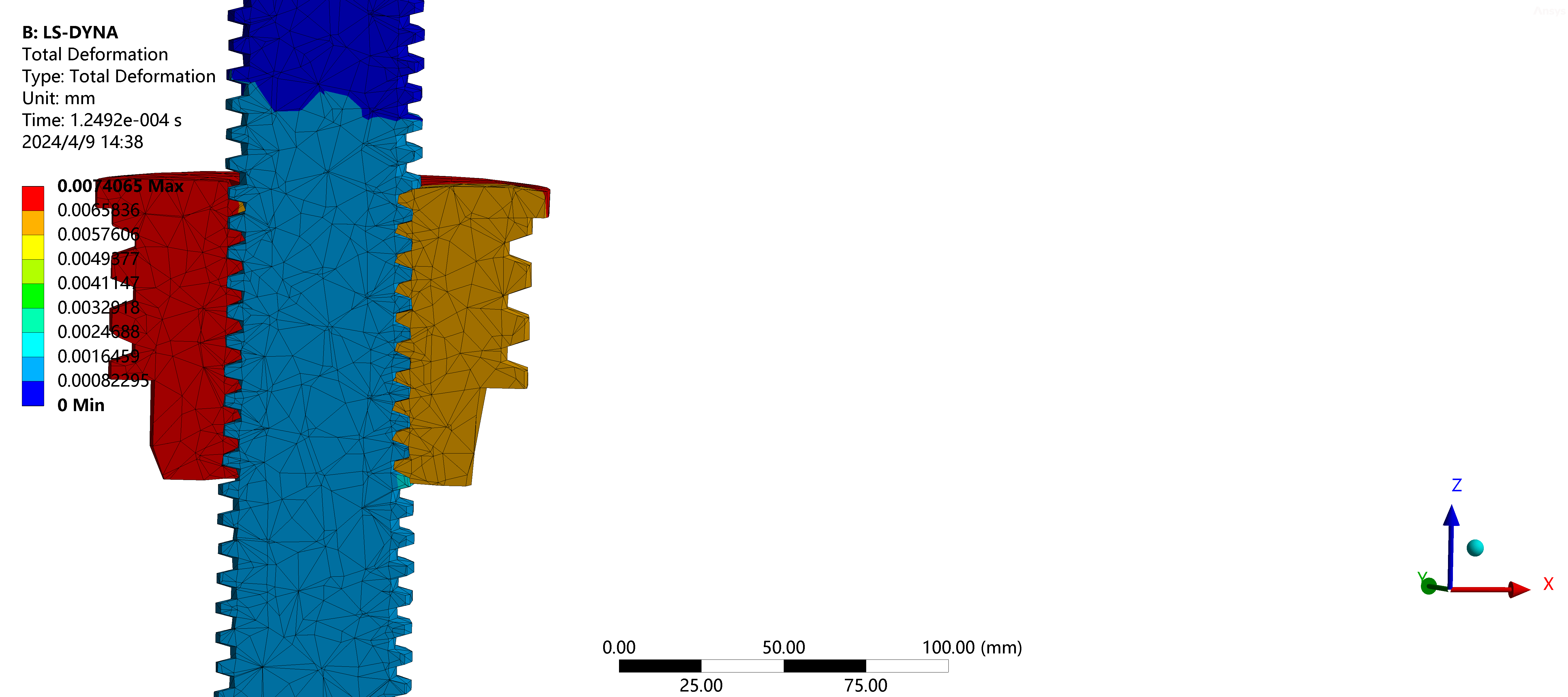

Supplement: Supplementary file 1 — Supplementary Information. [file 41598_2025_94144_MOESM1_ESM.zip › Simulation experiment result graph/Grid division result diagram/变形21.png]

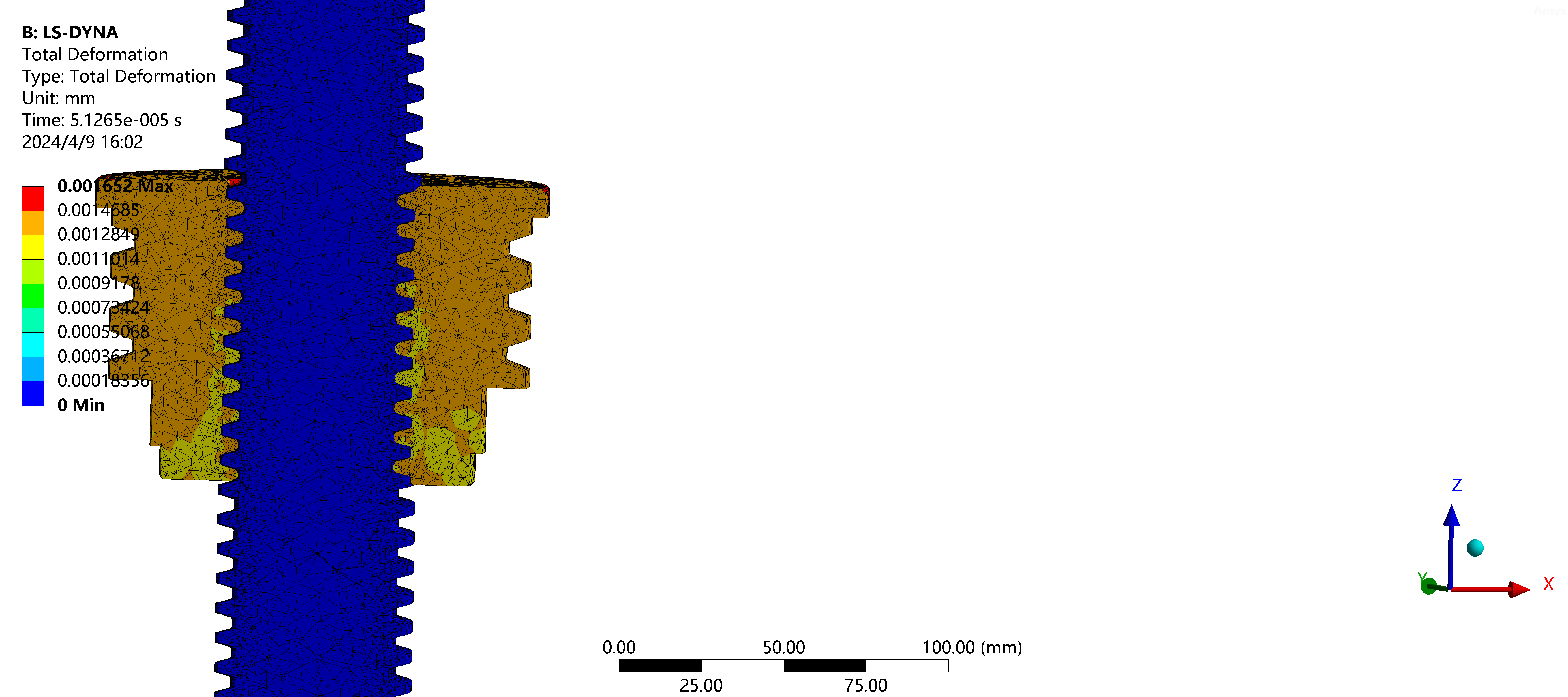

Supplement: Supplementary file 1 — Supplementary Information. [file 41598_2025_94144_MOESM1_ESM.zip › Simulation experiment result graph/Grid division result diagram/变形4.5.png]

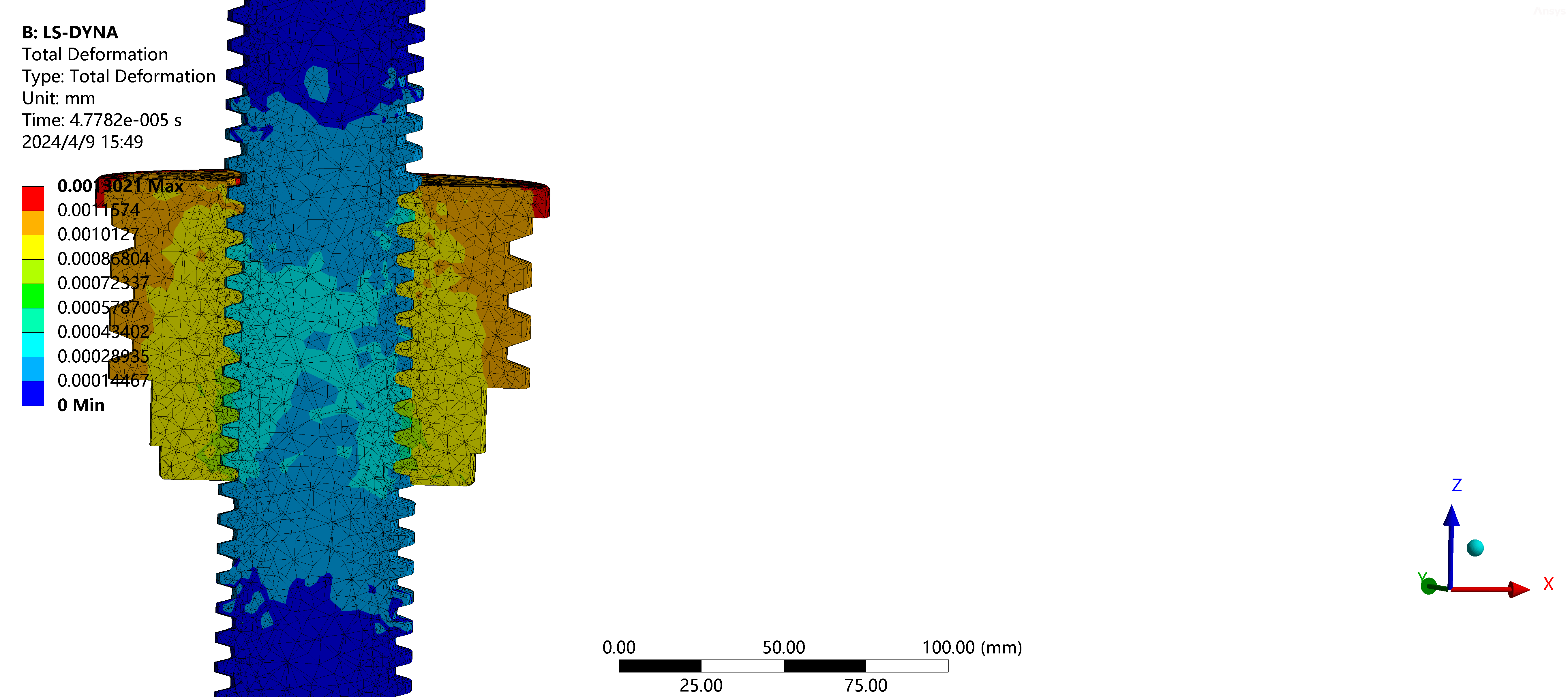

Supplement: Supplementary file 1 — Supplementary Information. [file 41598_2025_94144_MOESM1_ESM.zip › Simulation experiment result graph/Grid division result diagram/变形5.5.png]

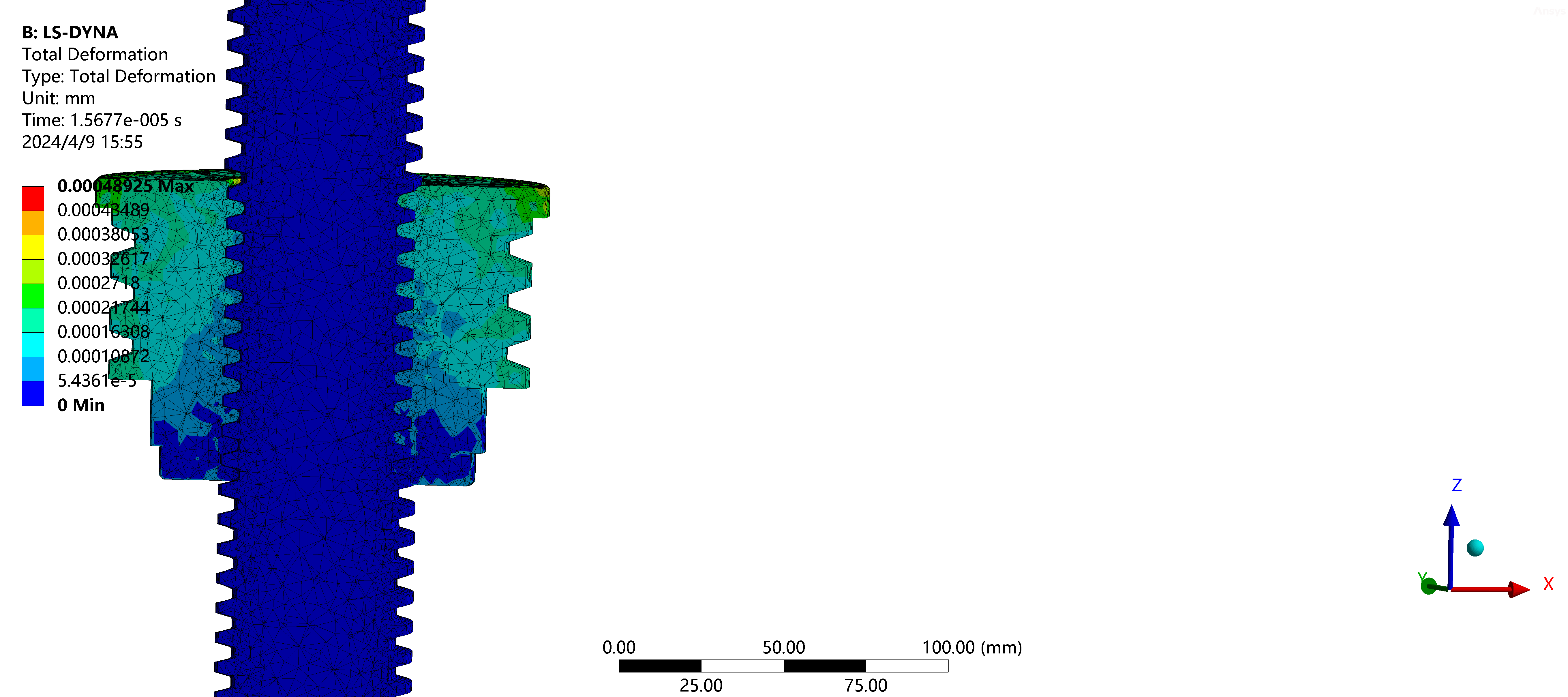

Supplement: Supplementary file 1 — Supplementary Information. [file 41598_2025_94144_MOESM1_ESM.zip › Simulation experiment result graph/Grid division result diagram/变形5.png]

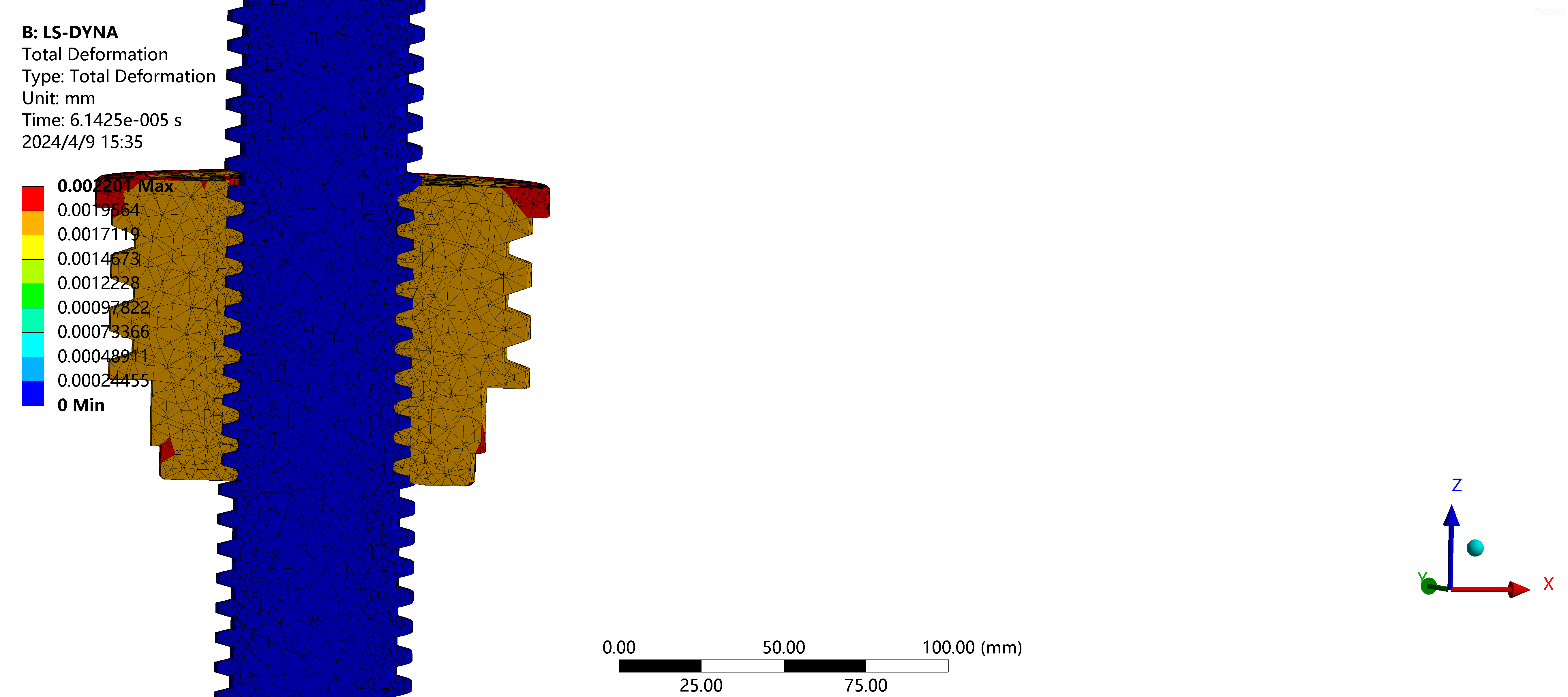

Supplement: Supplementary file 1 — Supplementary Information. [file 41598_2025_94144_MOESM1_ESM.zip › Simulation experiment result graph/Grid division result diagram/变形6.5.png]

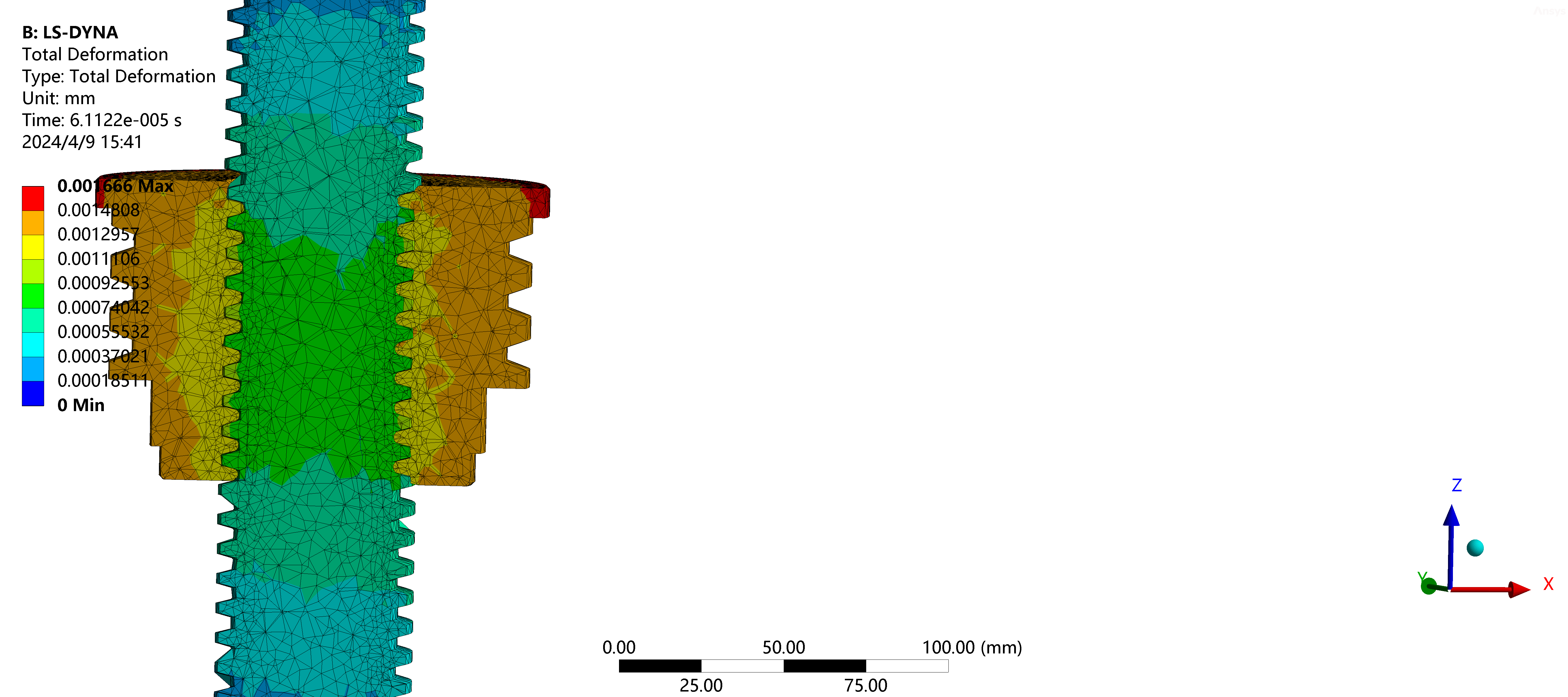

Supplement: Supplementary file 1 — Supplementary Information. [file 41598_2025_94144_MOESM1_ESM.zip › Simulation experiment result graph/Grid division result diagram/变形6.png]

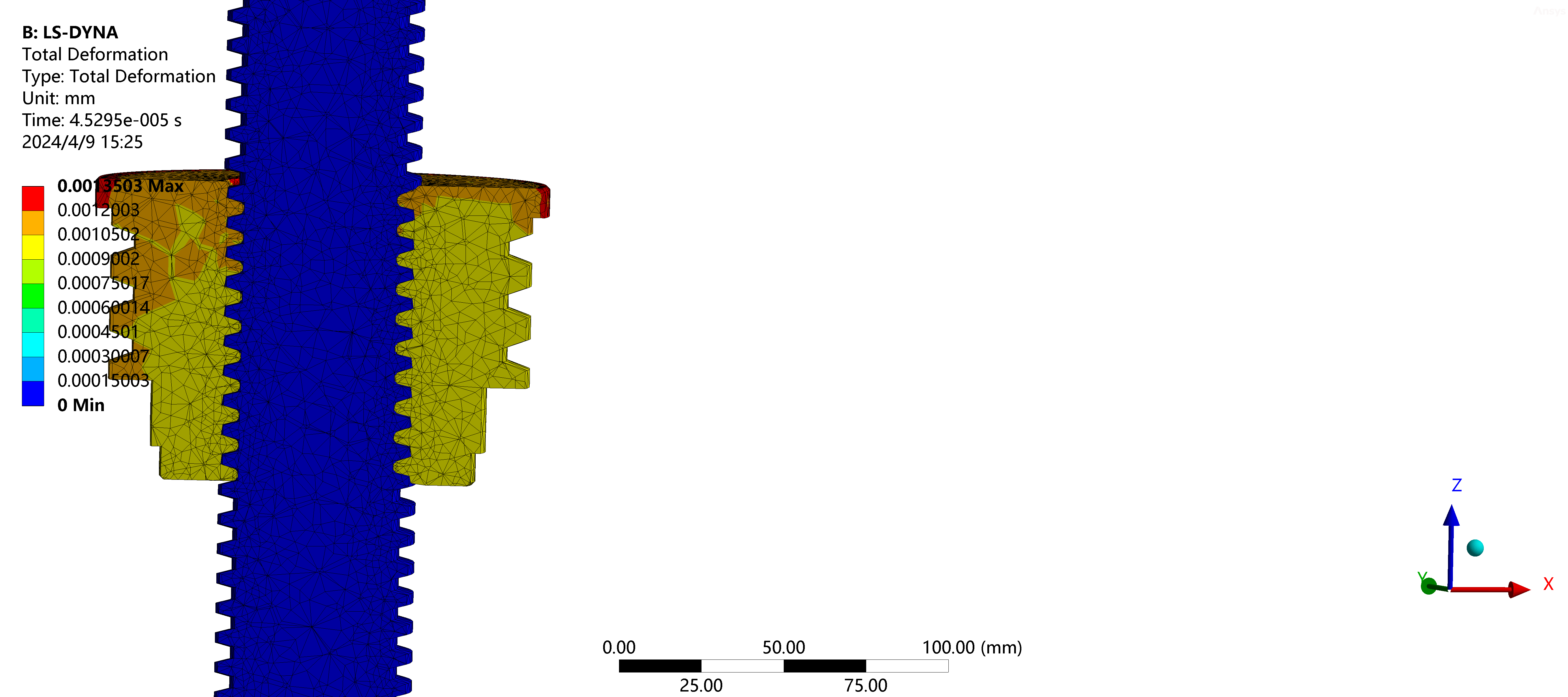

Supplement: Supplementary file 1 — Supplementary Information. [file 41598_2025_94144_MOESM1_ESM.zip › Simulation experiment result graph/Grid division result diagram/变形7.png]

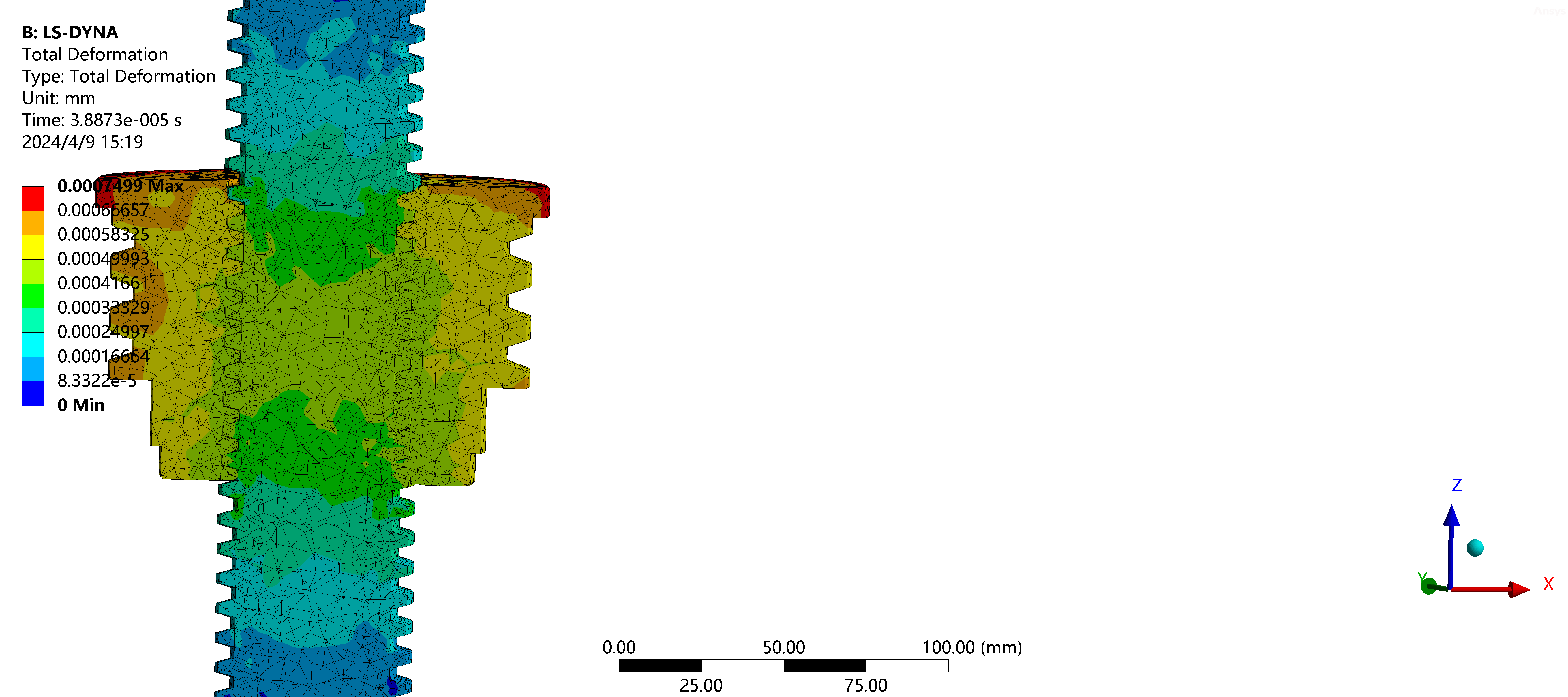

Supplement: Supplementary file 1 — Supplementary Information. [file 41598_2025_94144_MOESM1_ESM.zip › Simulation experiment result graph/Grid division result diagram/变形8.png]

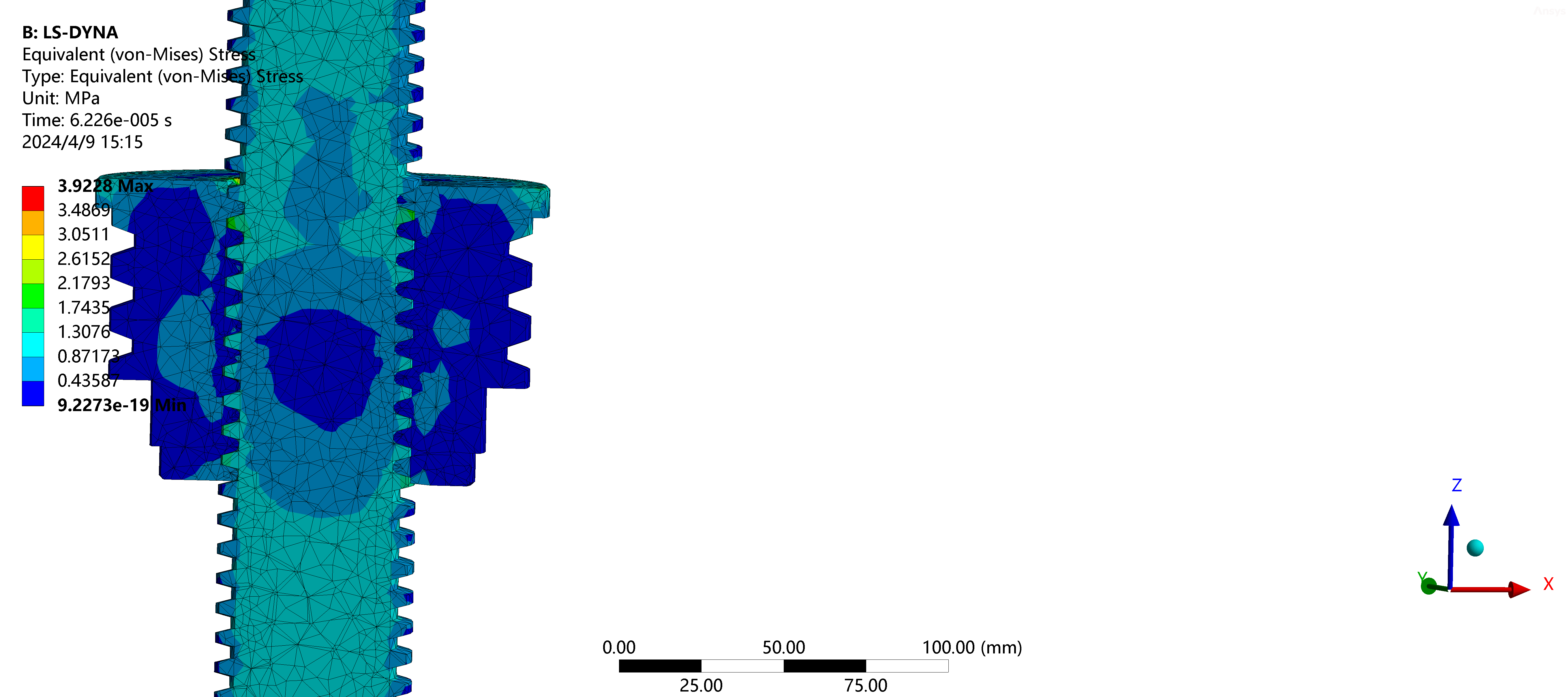

Supplement: Supplementary file 1 — Supplementary Information. [file 41598_2025_94144_MOESM1_ESM.zip › Simulation experiment result graph/Grid division result diagram/应力10.png]

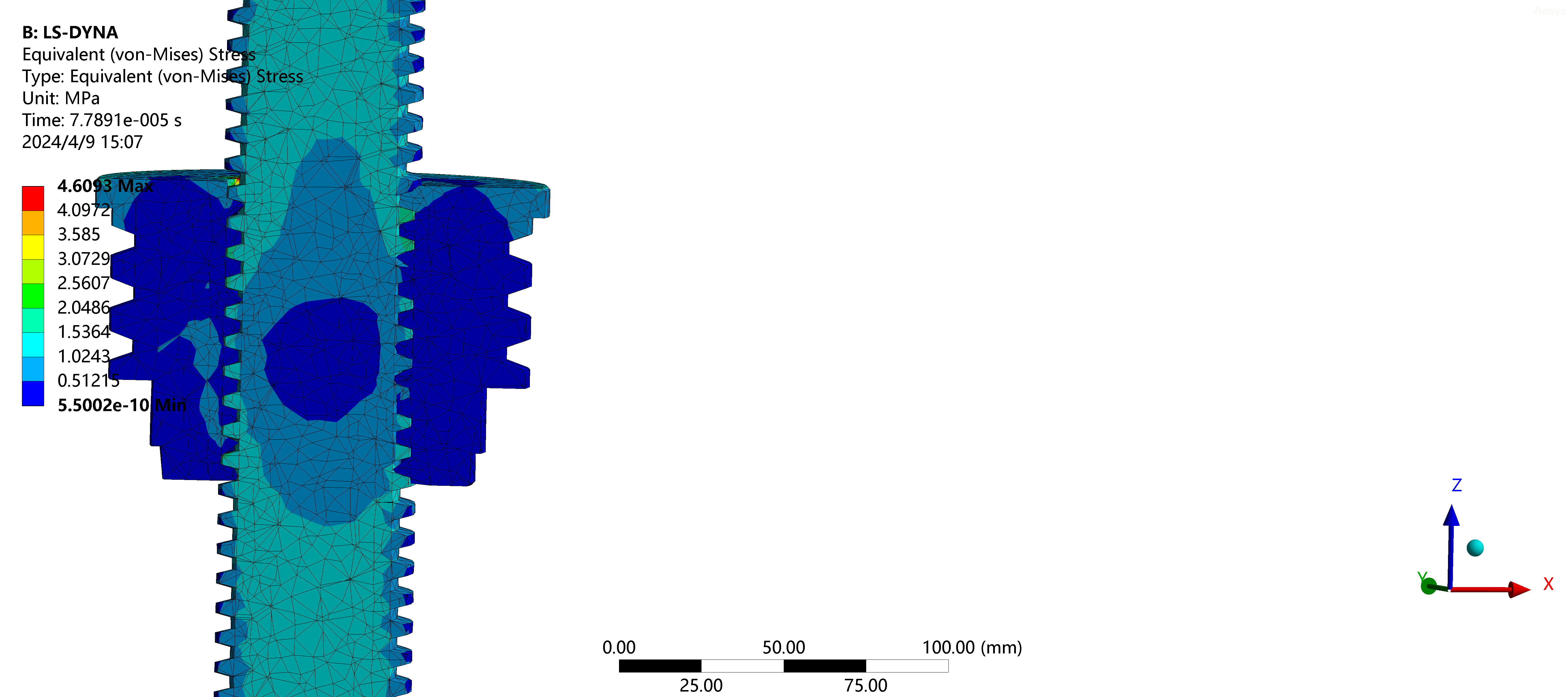

Supplement: Supplementary file 1 — Supplementary Information. [file 41598_2025_94144_MOESM1_ESM.zip › Simulation experiment result graph/Grid division result diagram/应力12.png]

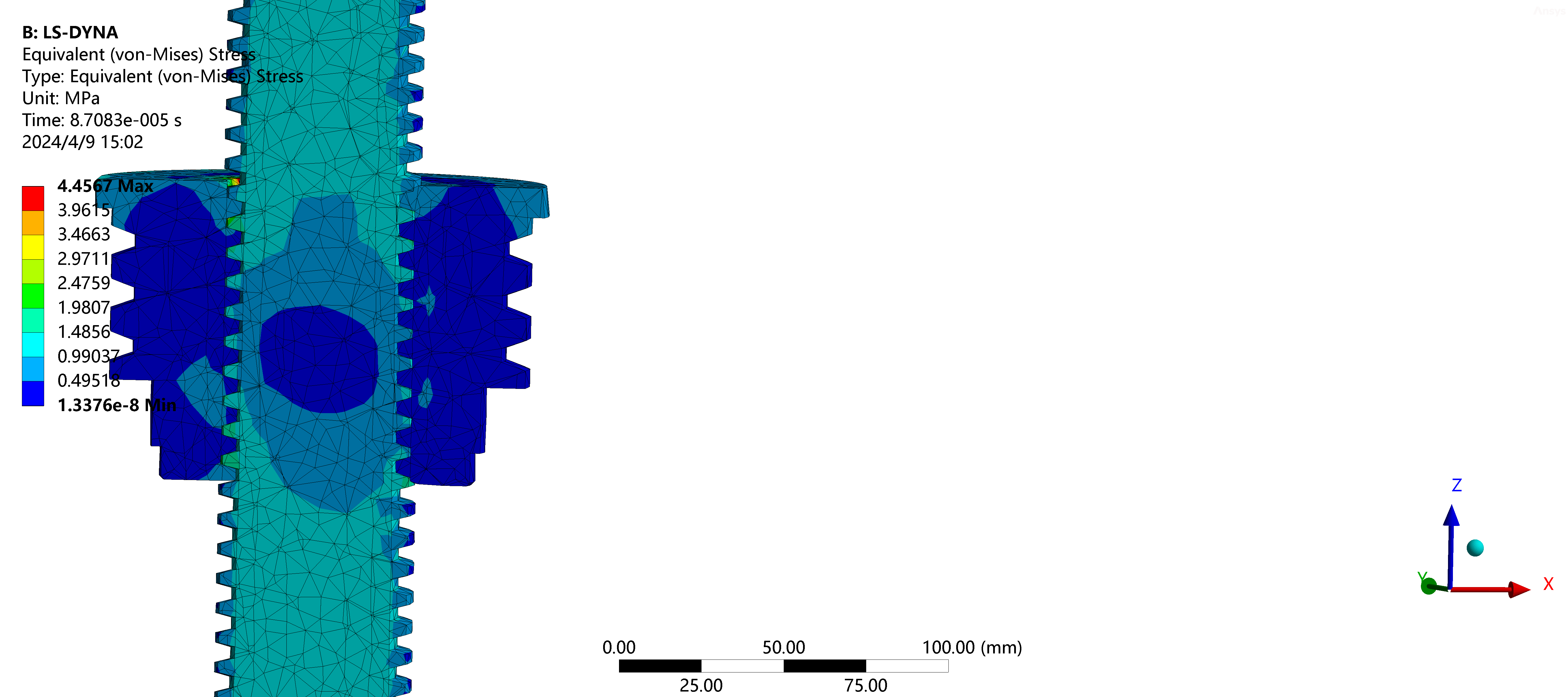

Supplement: Supplementary file 1 — Supplementary Information. [file 41598_2025_94144_MOESM1_ESM.zip › Simulation experiment result graph/Grid division result diagram/应力14.png]

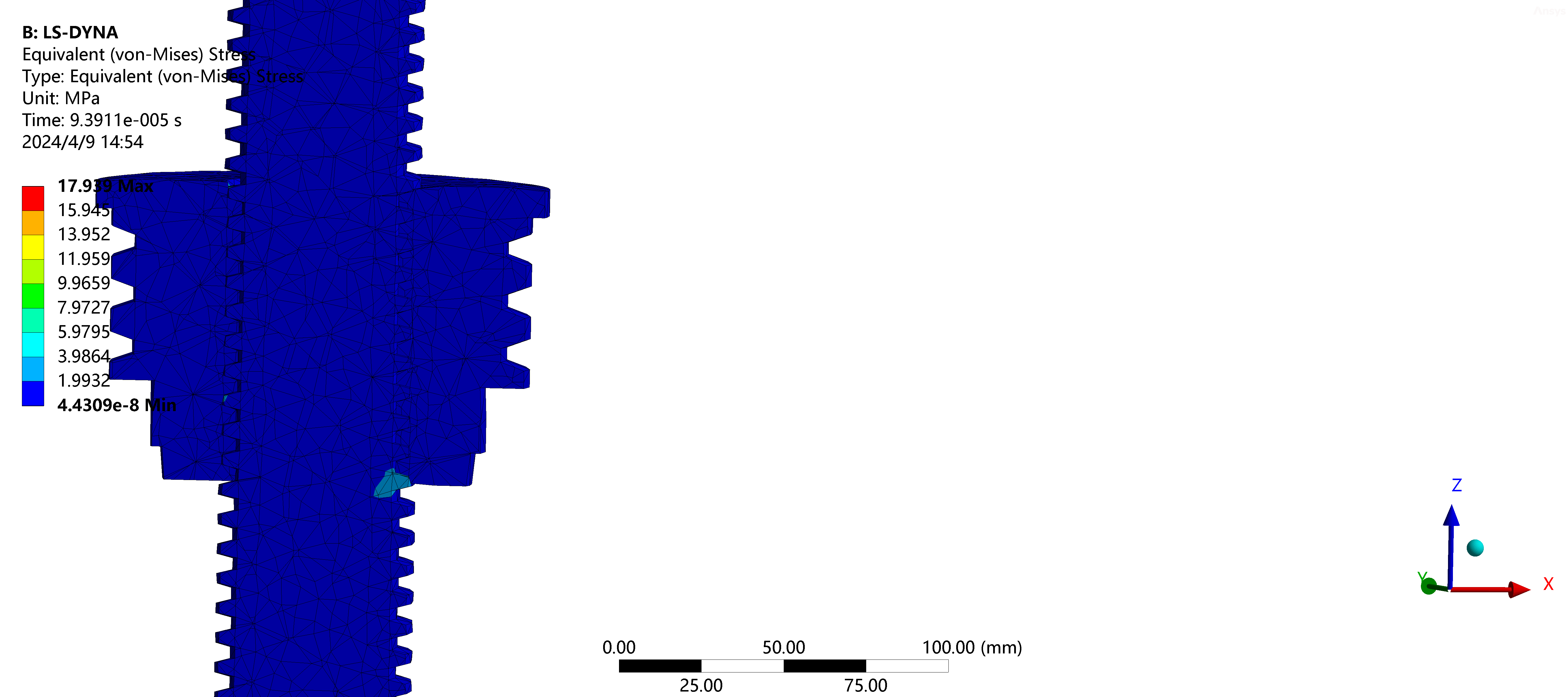

Supplement: Supplementary file 1 — Supplementary Information. [file 41598_2025_94144_MOESM1_ESM.zip › Simulation experiment result graph/Grid division result diagram/应力16.png]

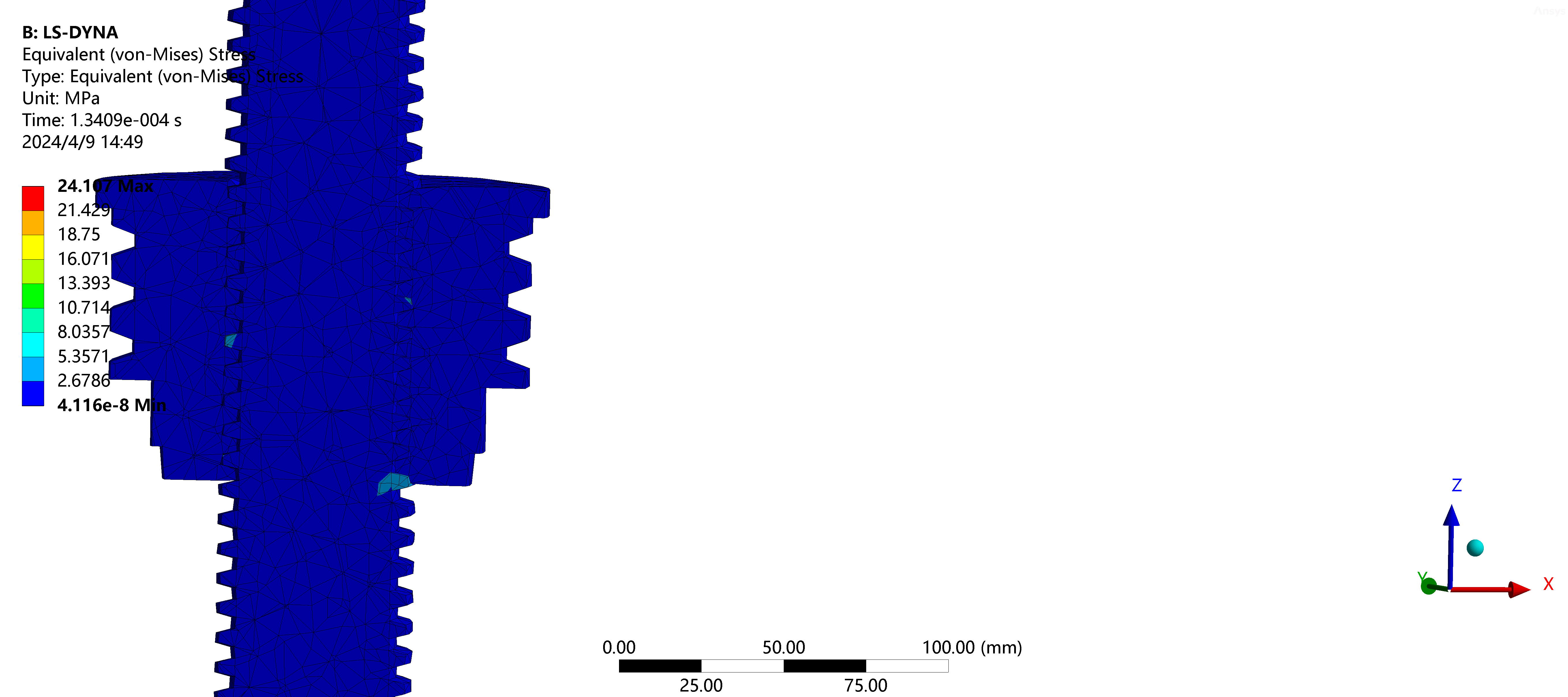

Supplement: Supplementary file 1 — Supplementary Information. [file 41598_2025_94144_MOESM1_ESM.zip › Simulation experiment result graph/Grid division result diagram/应力18.png]

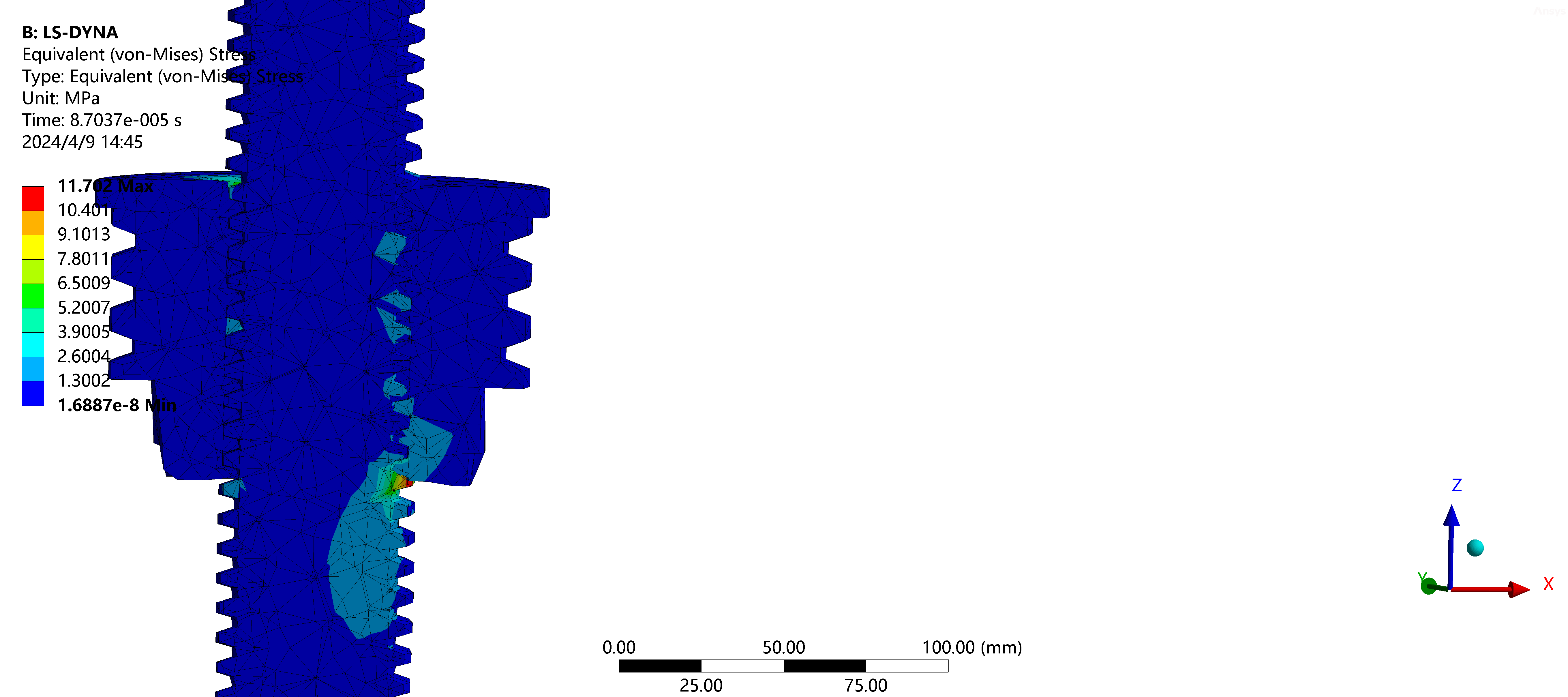

Supplement: Supplementary file 1 — Supplementary Information. [file 41598_2025_94144_MOESM1_ESM.zip › Simulation experiment result graph/Grid division result diagram/应力20.png]

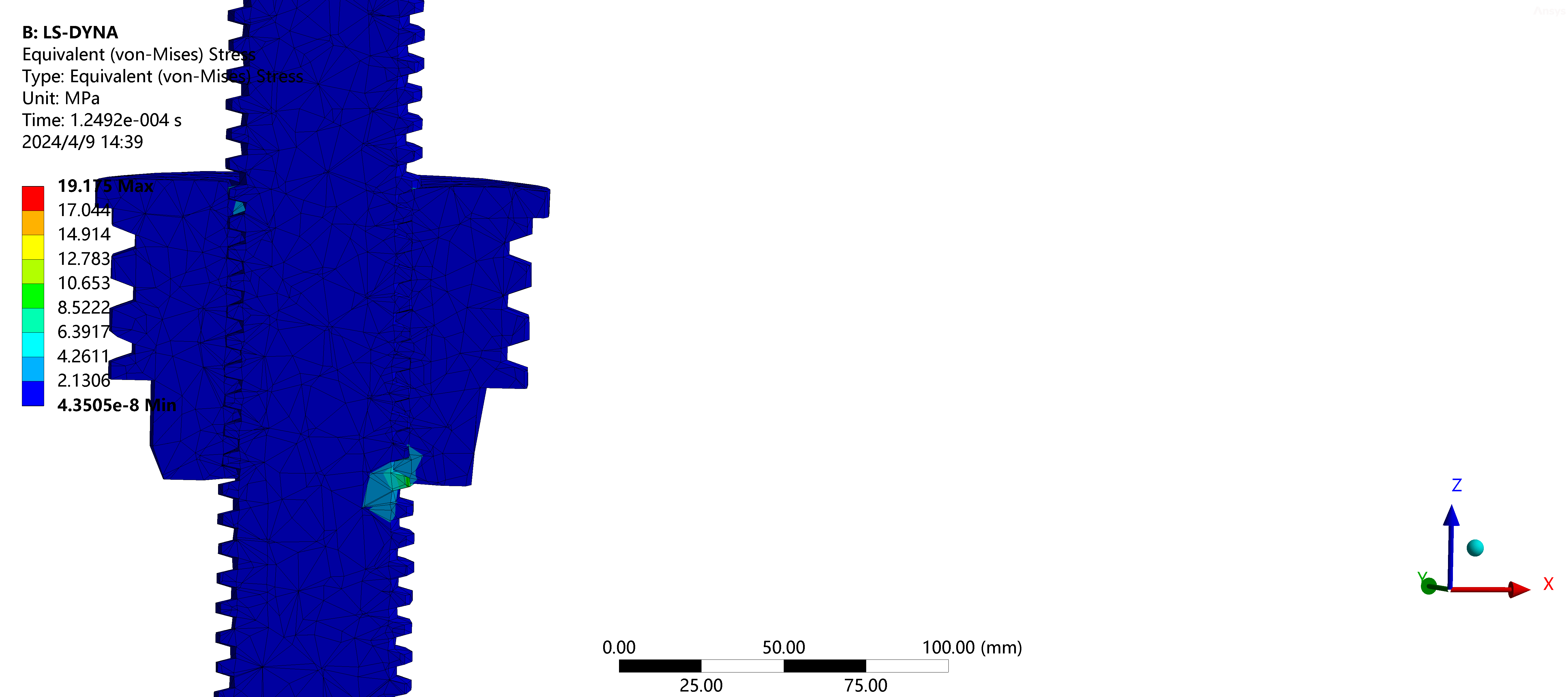

Supplement: Supplementary file 1 — Supplementary Information. [file 41598_2025_94144_MOESM1_ESM.zip › Simulation experiment result graph/Grid division result diagram/应力21.png]

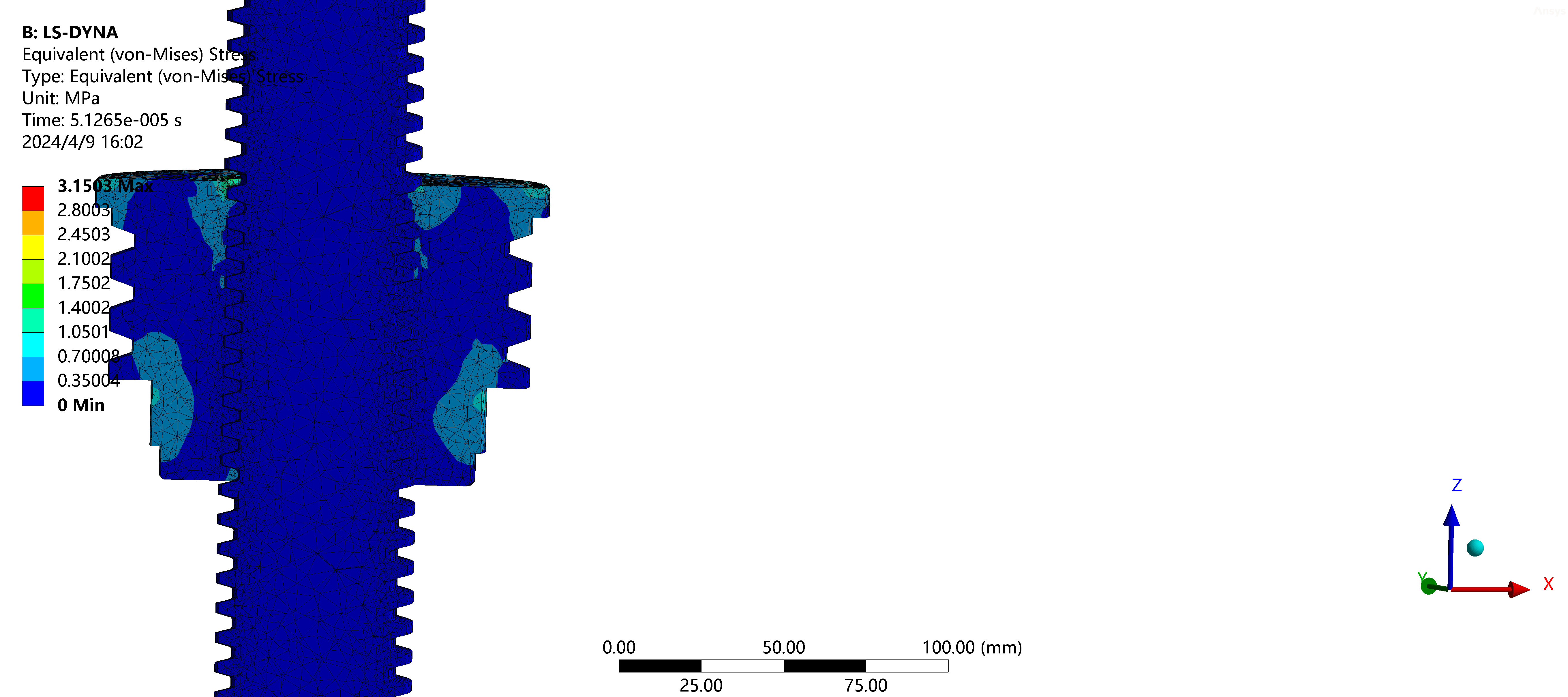

Supplement: Supplementary file 1 — Supplementary Information. [file 41598_2025_94144_MOESM1_ESM.zip › Simulation experiment result graph/Grid division result diagram/应力4.5.png]

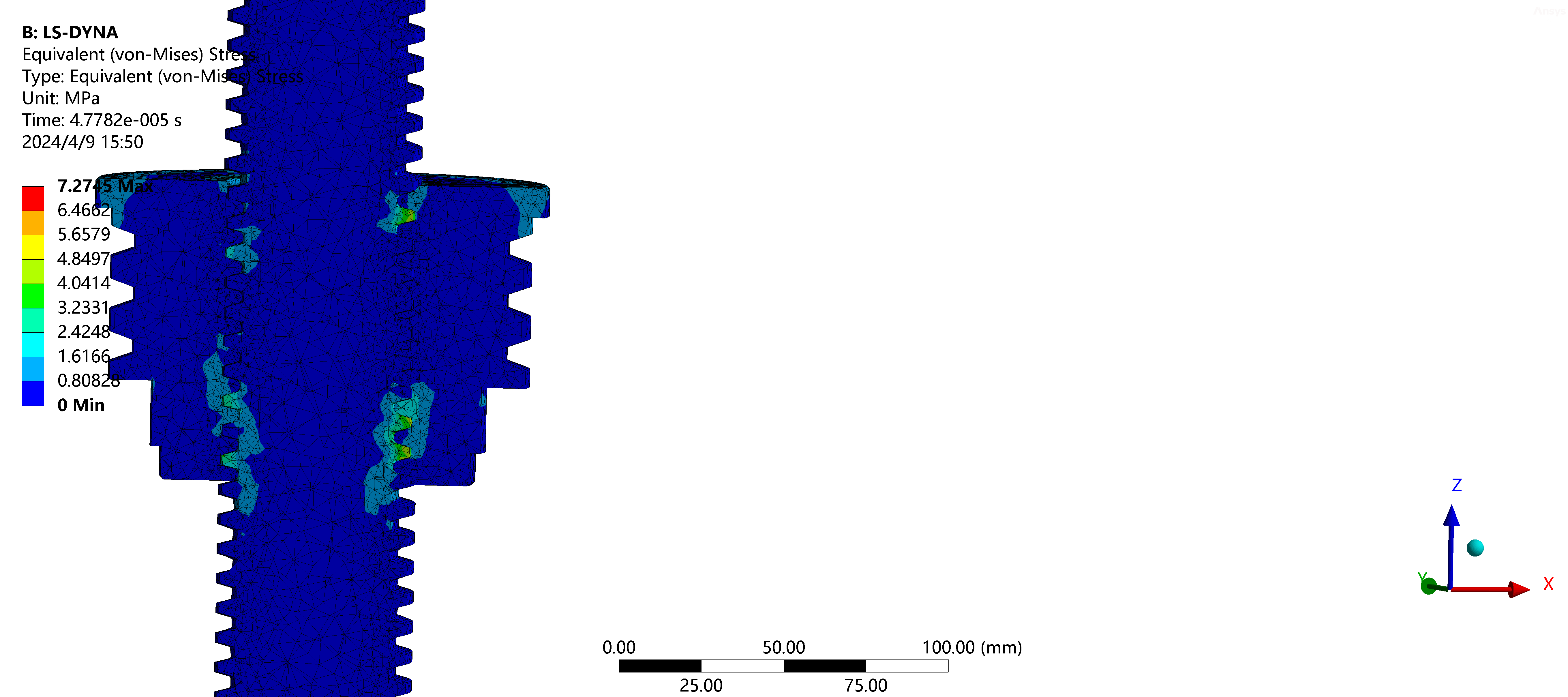

Supplement: Supplementary file 1 — Supplementary Information. [file 41598_2025_94144_MOESM1_ESM.zip › Simulation experiment result graph/Grid division result diagram/应力5.5.png]

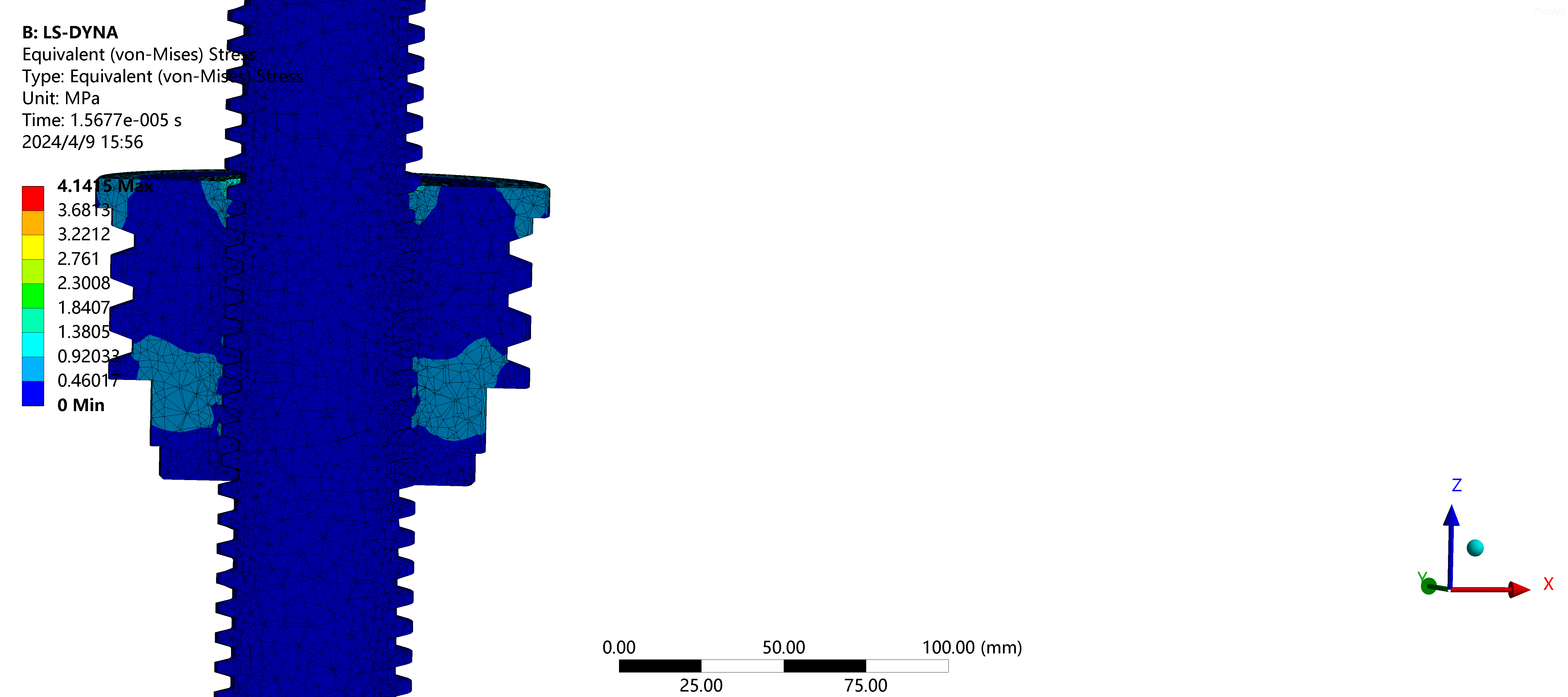

Supplement: Supplementary file 1 — Supplementary Information. [file 41598_2025_94144_MOESM1_ESM.zip › Simulation experiment result graph/Grid division result diagram/应力5.png]

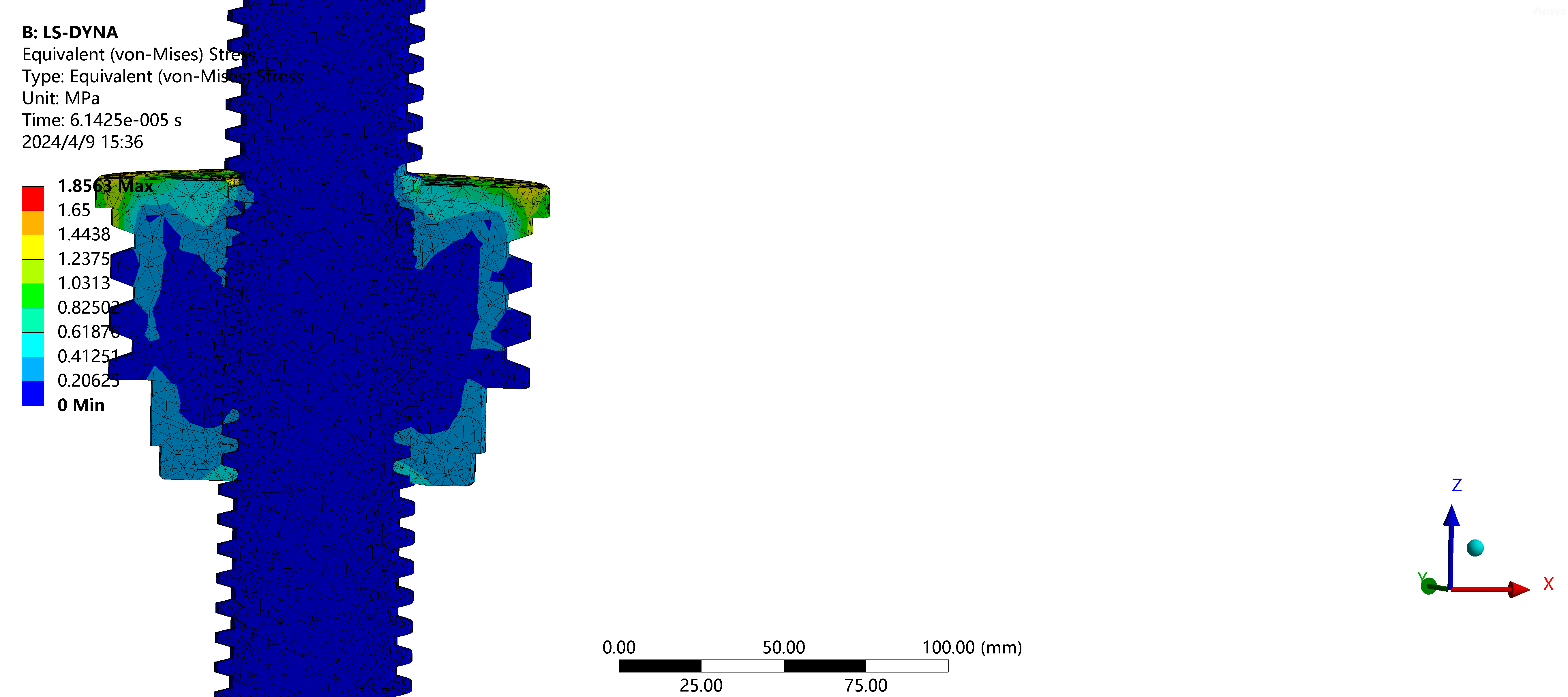

Supplement: Supplementary file 1 — Supplementary Information. [file 41598_2025_94144_MOESM1_ESM.zip › Simulation experiment result graph/Grid division result diagram/应力6.5.png]

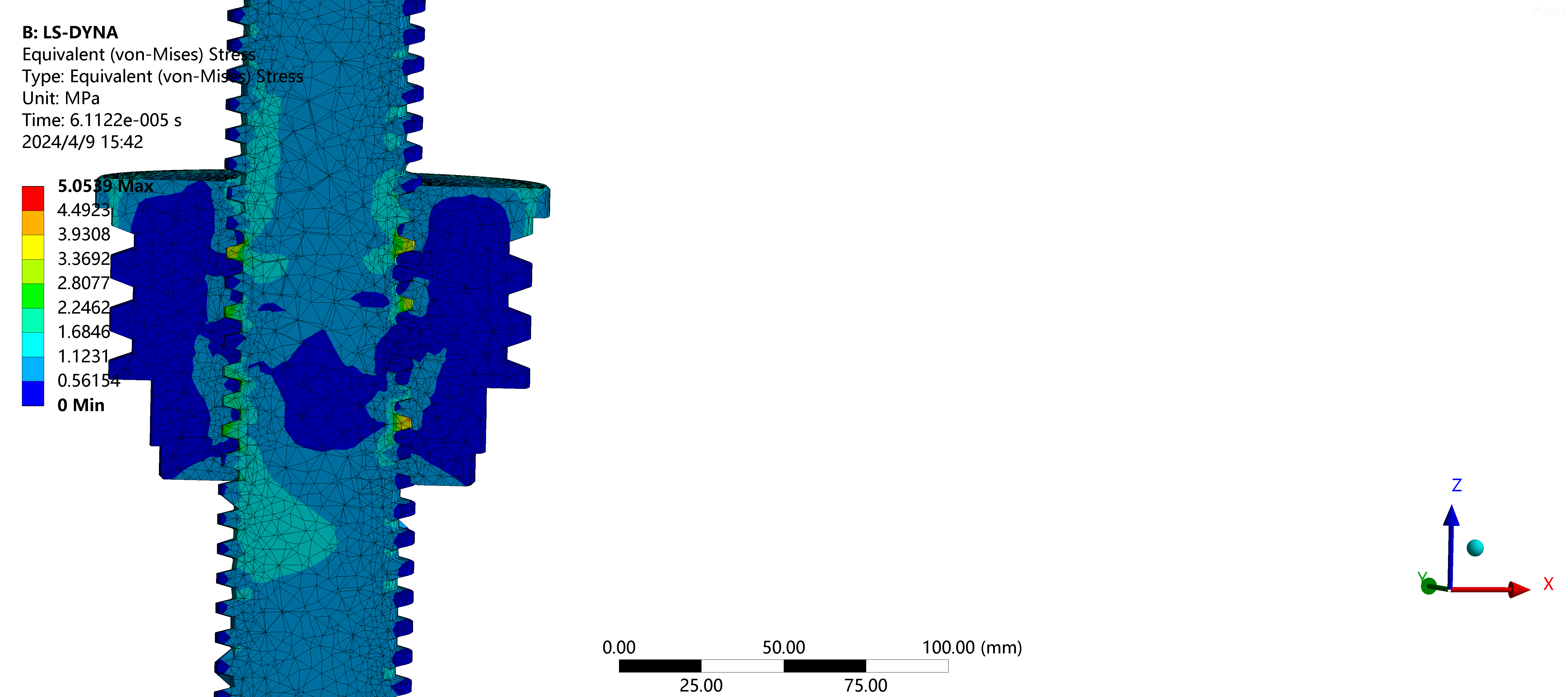

Supplement: Supplementary file 1 — Supplementary Information. [file 41598_2025_94144_MOESM1_ESM.zip › Simulation experiment result graph/Grid division result diagram/应力6.png]

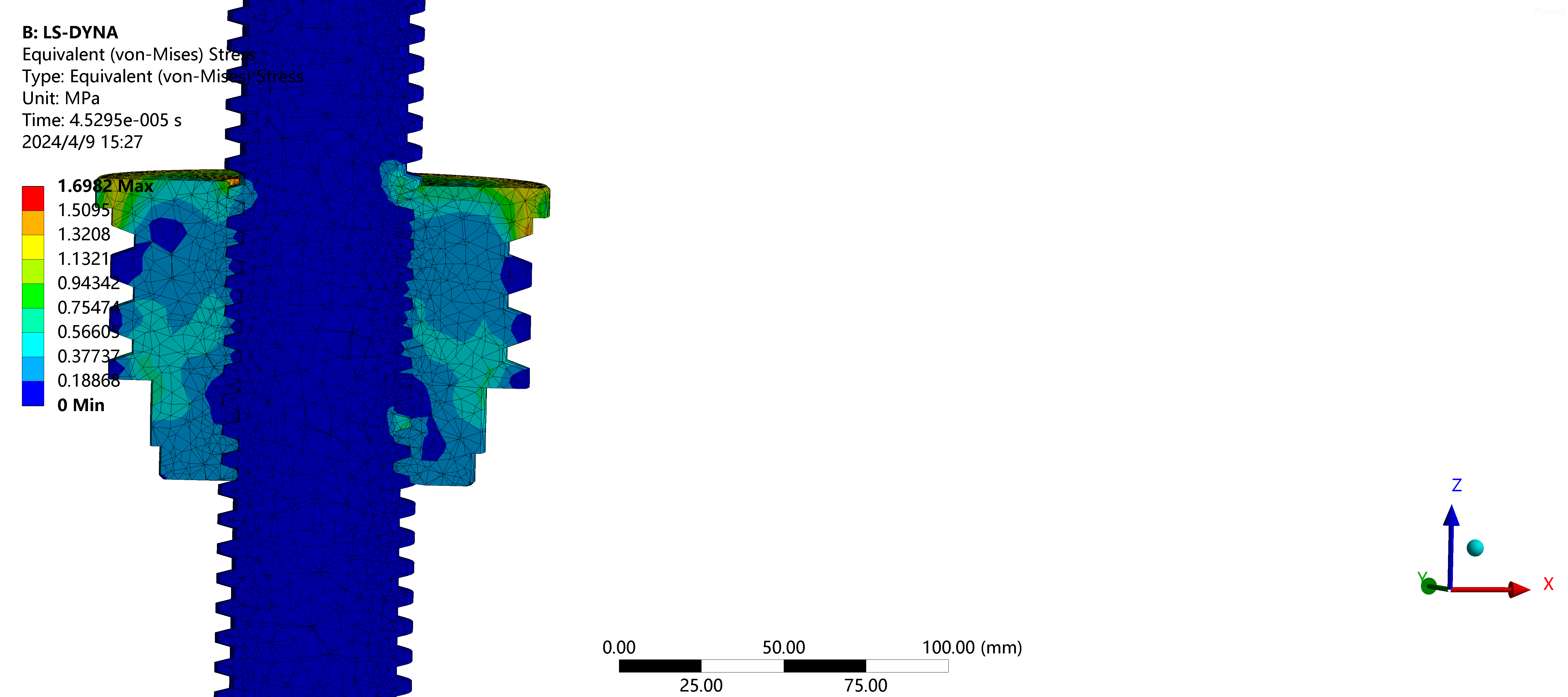

Supplement: Supplementary file 1 — Supplementary Information. [file 41598_2025_94144_MOESM1_ESM.zip › Simulation experiment result graph/Grid division result diagram/应力7.png]

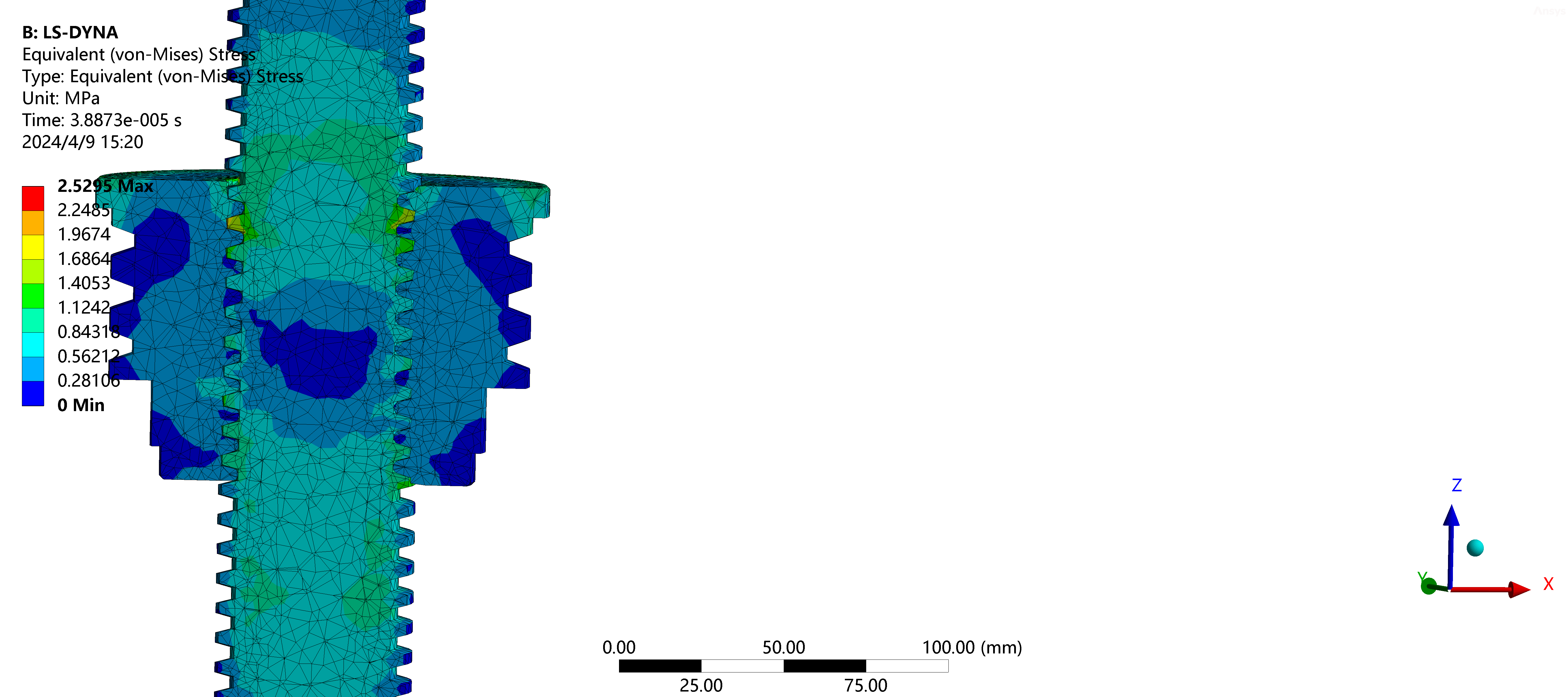

Supplement: Supplementary file 1 — Supplementary Information. [file 41598_2025_94144_MOESM1_ESM.zip › Simulation experiment result graph/Grid division result diagram/应力8.png]

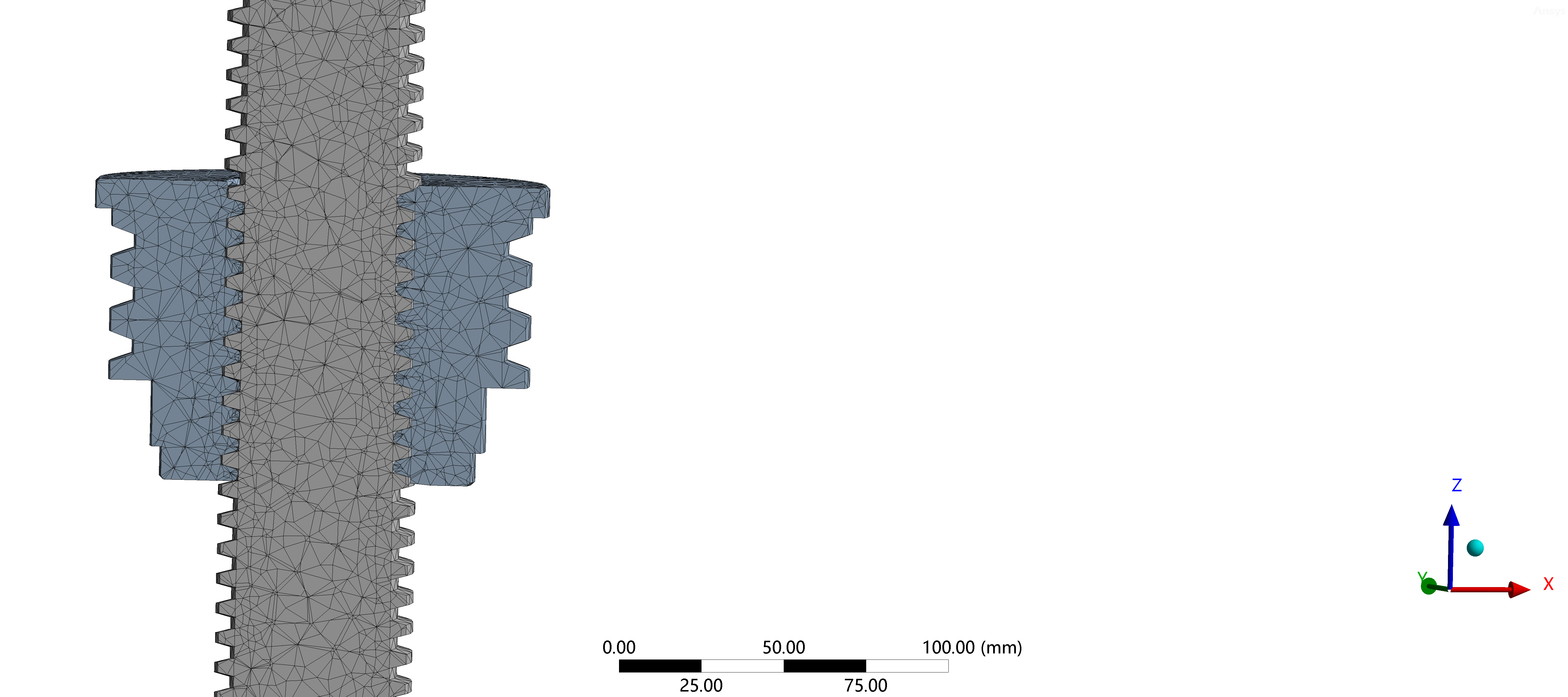

Supplement: Supplementary file 1 — Supplementary Information. [file 41598_2025_94144_MOESM1_ESM.zip › Simulation experiment result graph/Grid division result diagram/网格10.png]

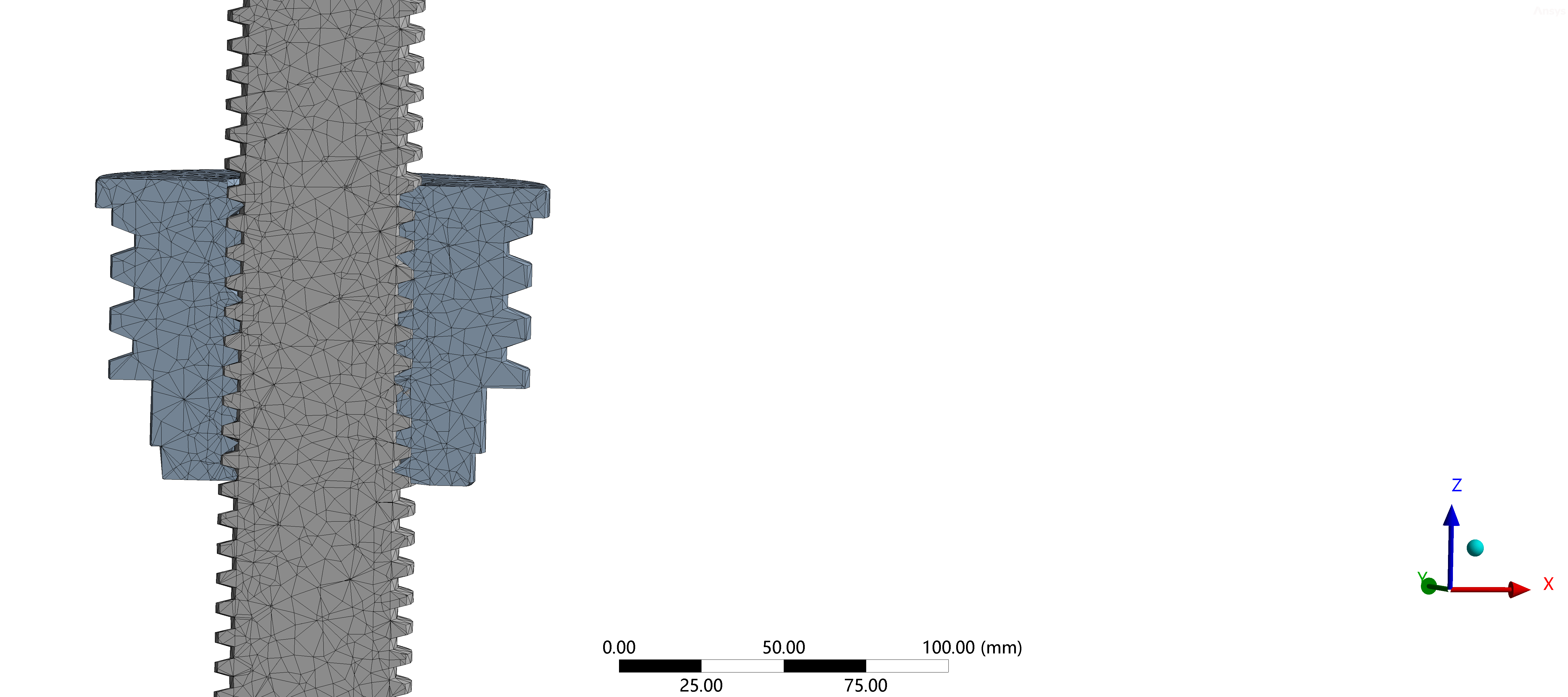

Supplement: Supplementary file 1 — Supplementary Information. [file 41598_2025_94144_MOESM1_ESM.zip › Simulation experiment result graph/Grid division result diagram/网格12.png]

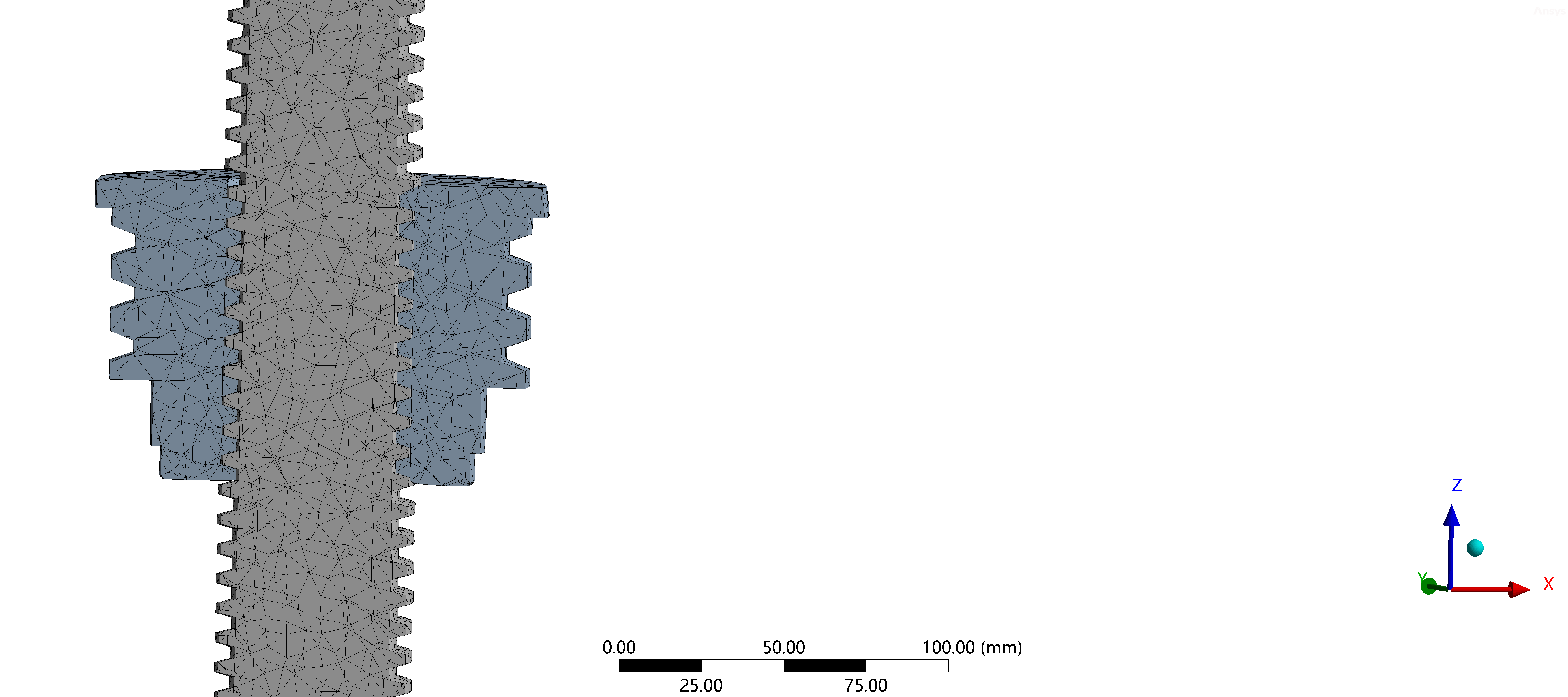

Supplement: Supplementary file 1 — Supplementary Information. [file 41598_2025_94144_MOESM1_ESM.zip › Simulation experiment result graph/Grid division result diagram/网格14.png]

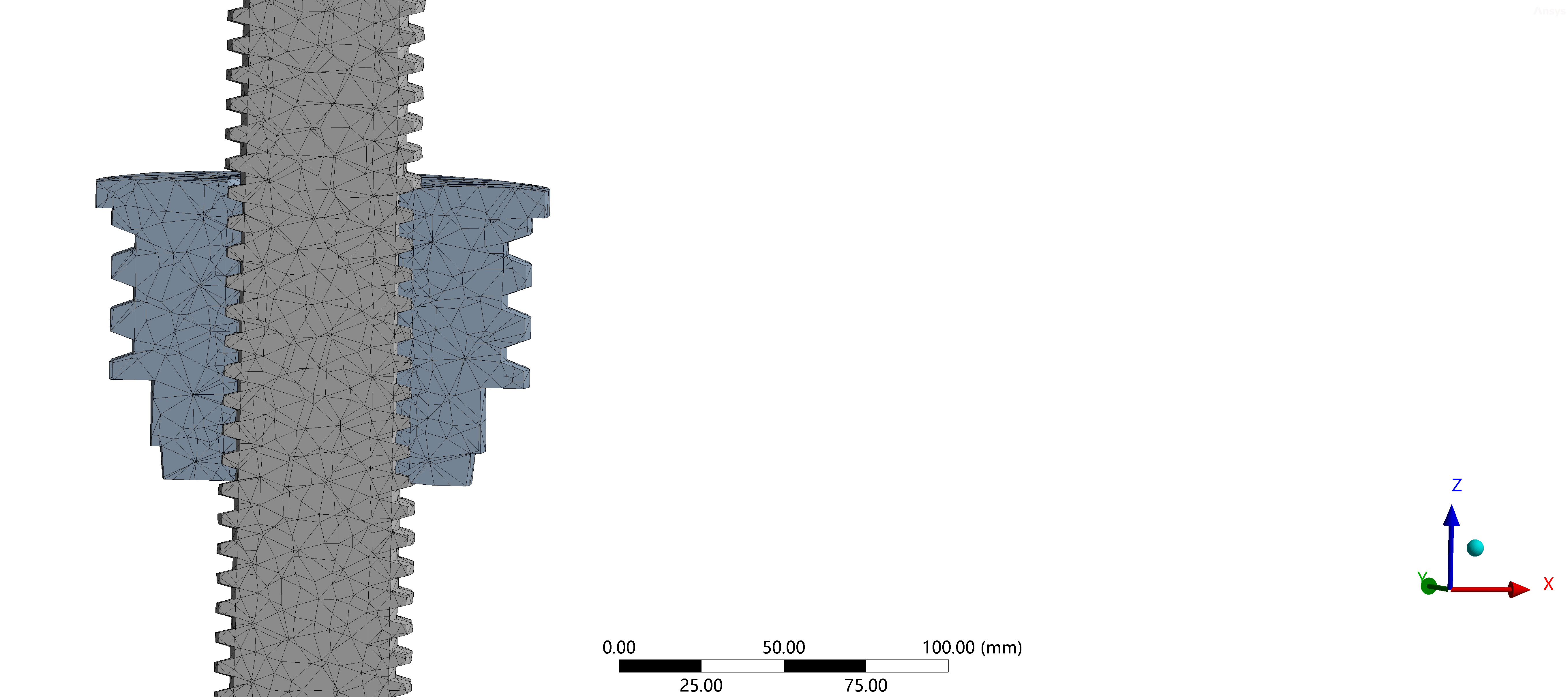

Supplement: Supplementary file 1 — Supplementary Information. [file 41598_2025_94144_MOESM1_ESM.zip › Simulation experiment result graph/Grid division result diagram/网格16.png]

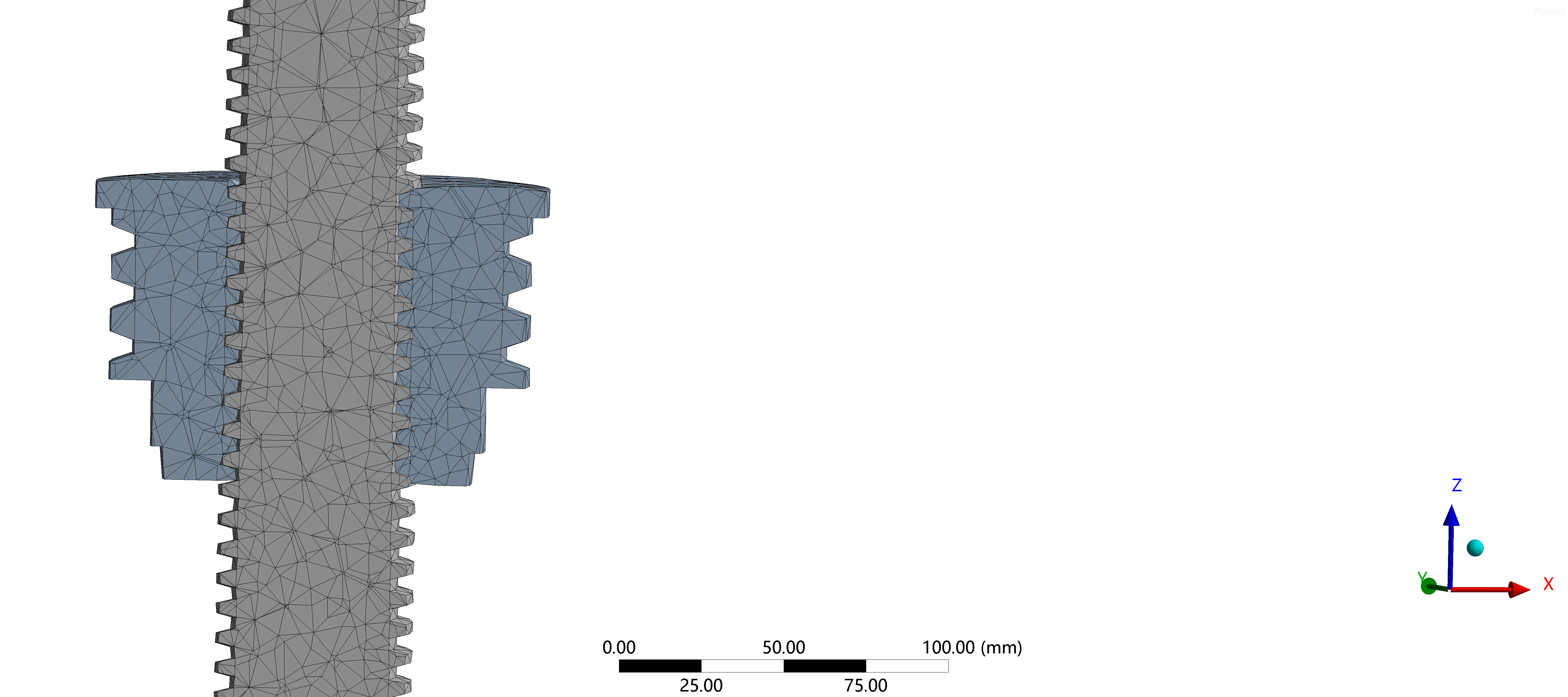

Supplement: Supplementary file 1 — Supplementary Information. [file 41598_2025_94144_MOESM1_ESM.zip › Simulation experiment result graph/Grid division result diagram/网格18.png]

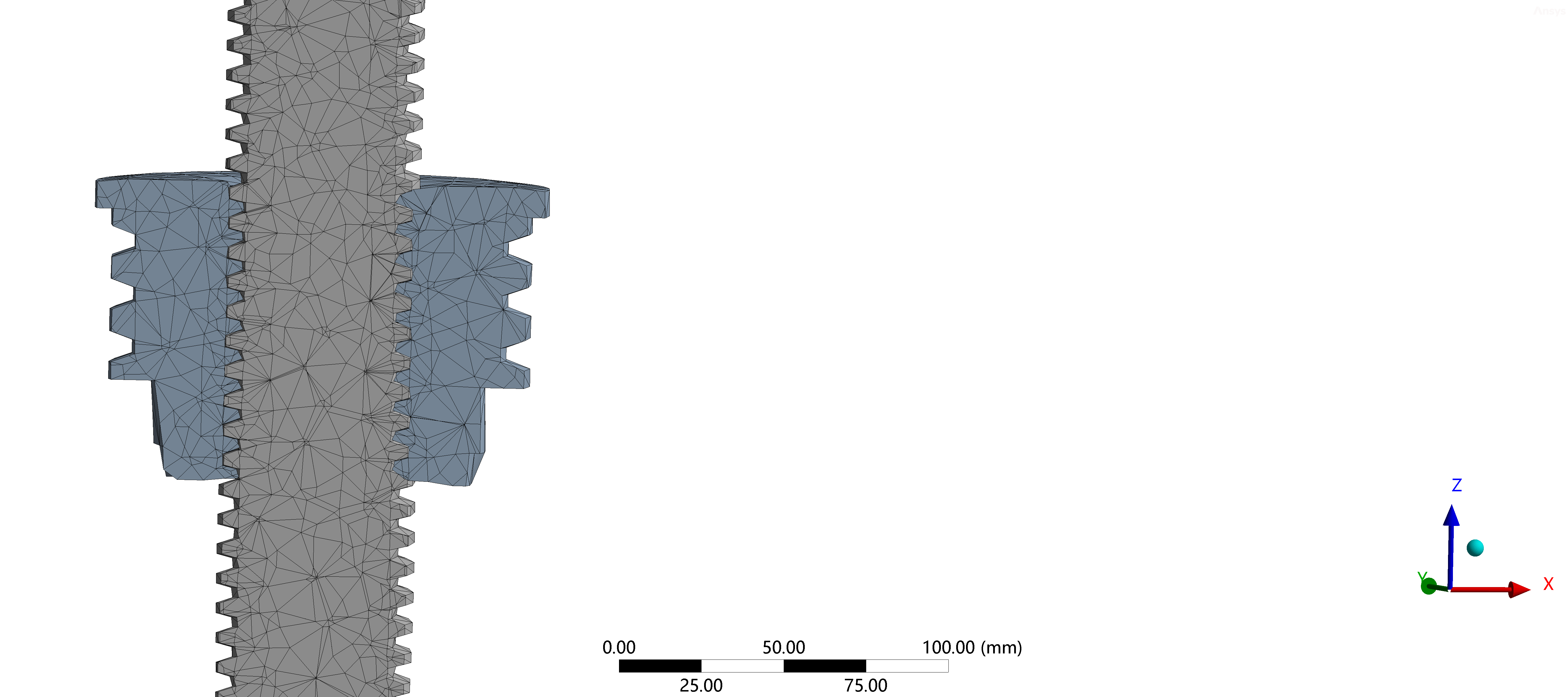

Supplement: Supplementary file 1 — Supplementary Information. [file 41598_2025_94144_MOESM1_ESM.zip › Simulation experiment result graph/Grid division result diagram/网格20.png]

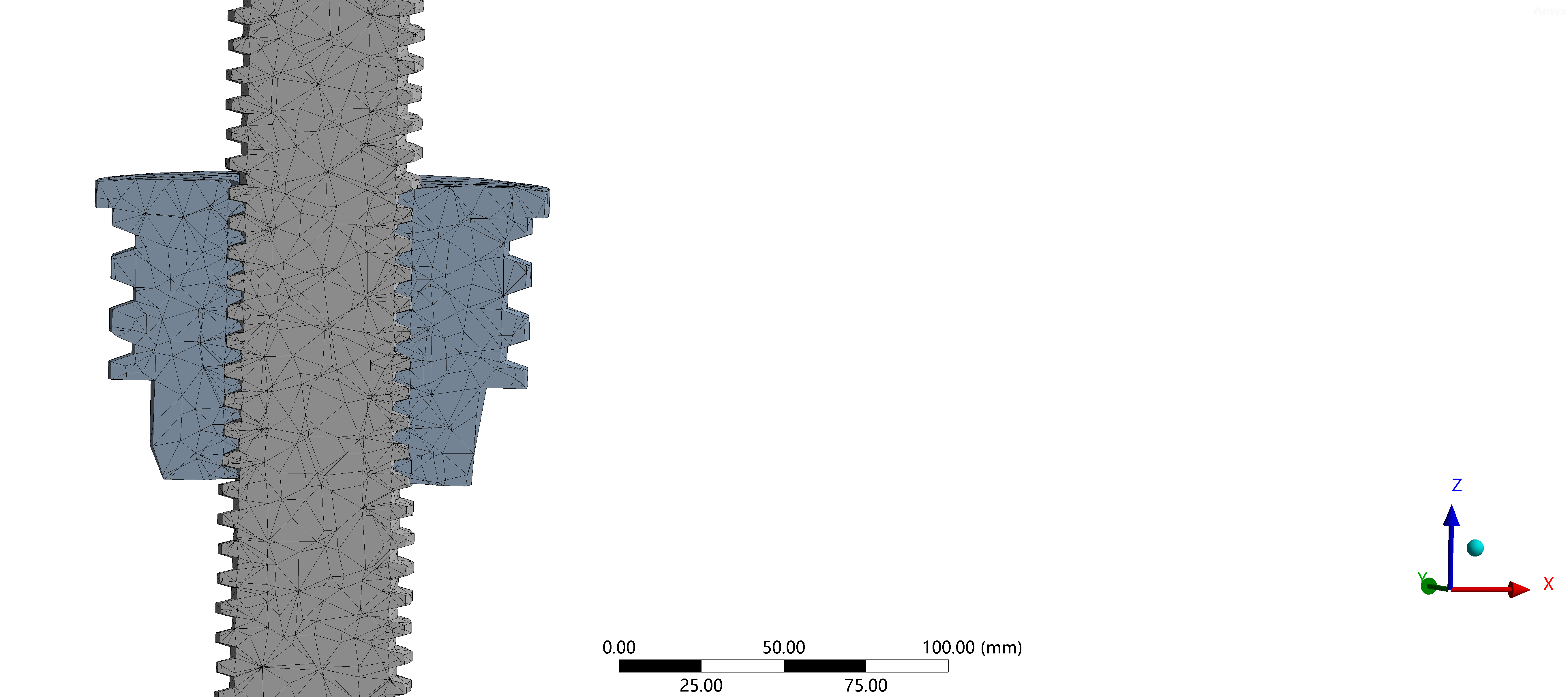

Supplement: Supplementary file 1 — Supplementary Information. [file 41598_2025_94144_MOESM1_ESM.zip › Simulation experiment result graph/Grid division result diagram/网格21.png]

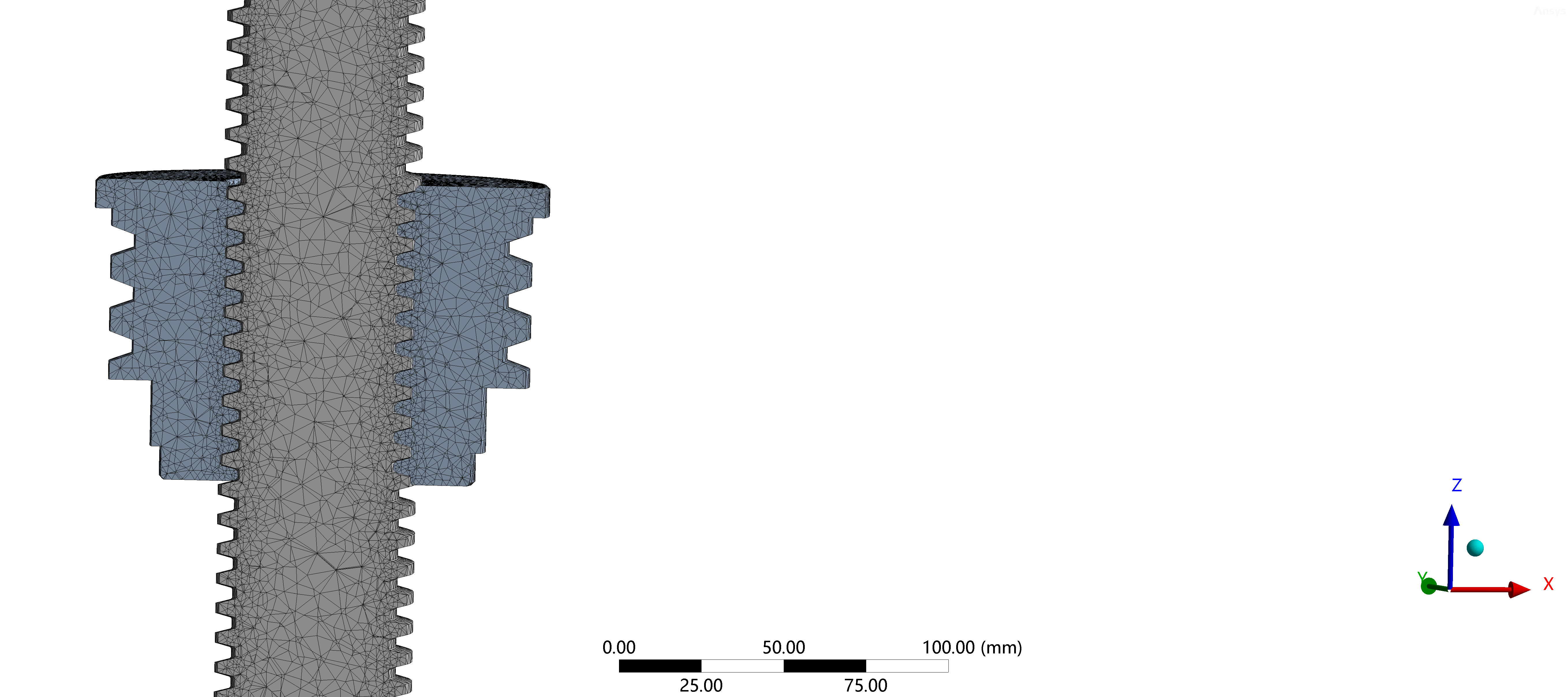

Supplement: Supplementary file 1 — Supplementary Information. [file 41598_2025_94144_MOESM1_ESM.zip › Simulation experiment result graph/Grid division result diagram/网格4.5.png]

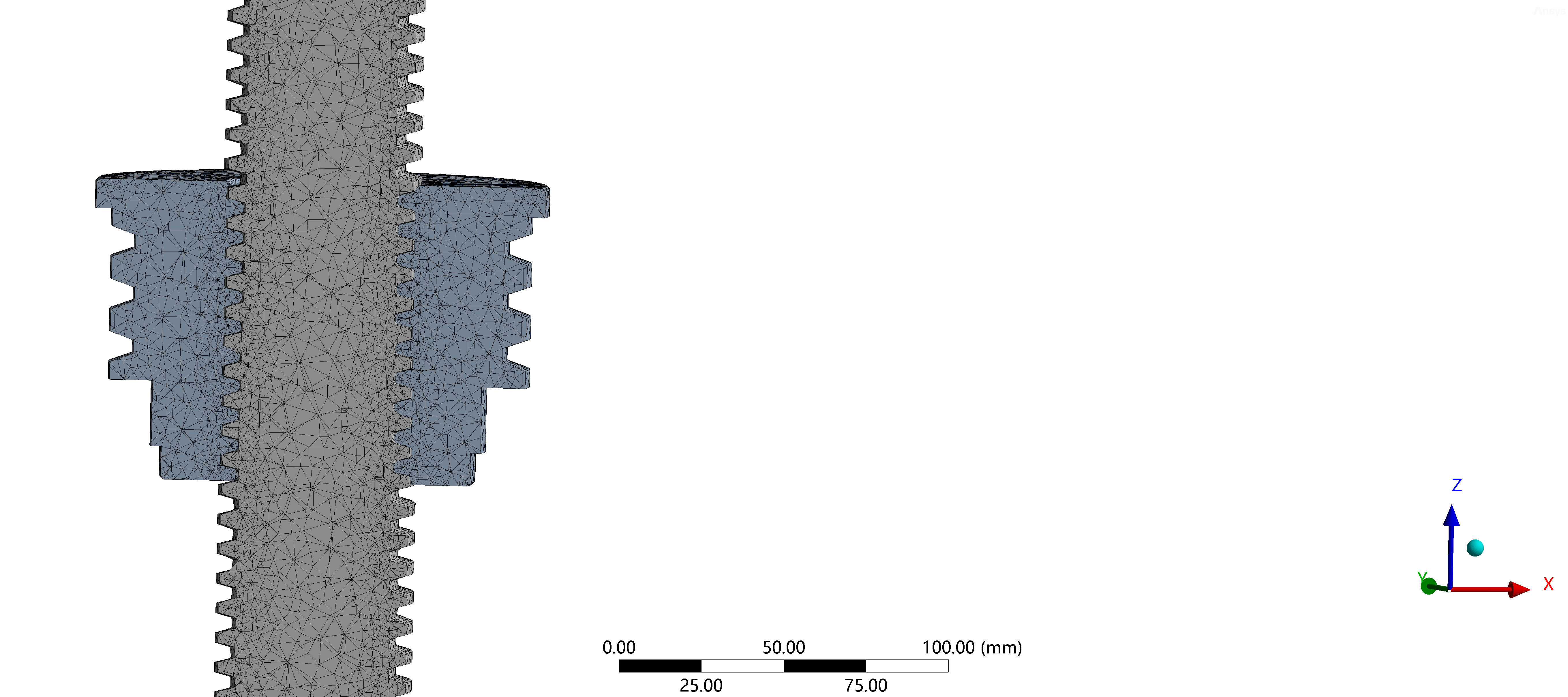

Supplement: Supplementary file 1 — Supplementary Information. [file 41598_2025_94144_MOESM1_ESM.zip › Simulation experiment result graph/Grid division result diagram/网格5.5.png]

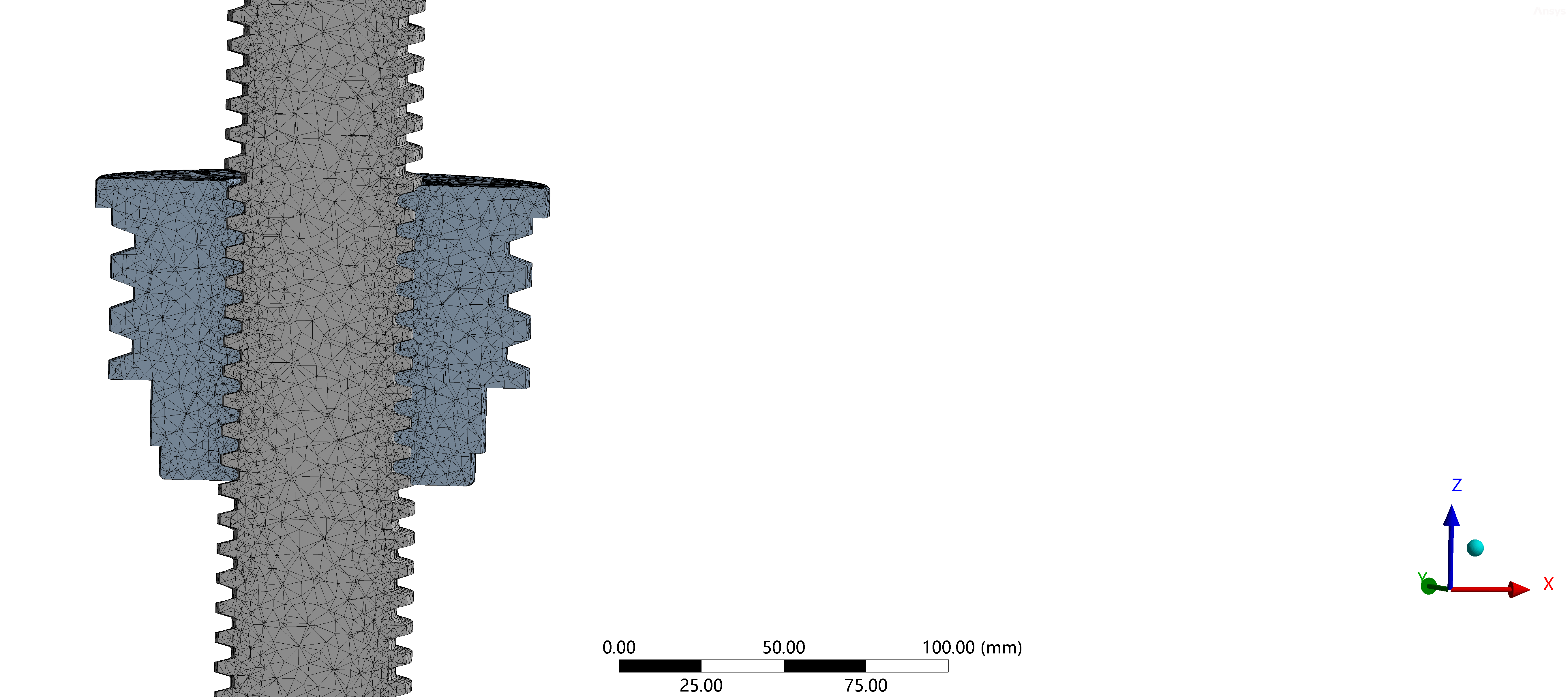

Supplement: Supplementary file 1 — Supplementary Information. [file 41598_2025_94144_MOESM1_ESM.zip › Simulation experiment result graph/Grid division result diagram/网格5.png]

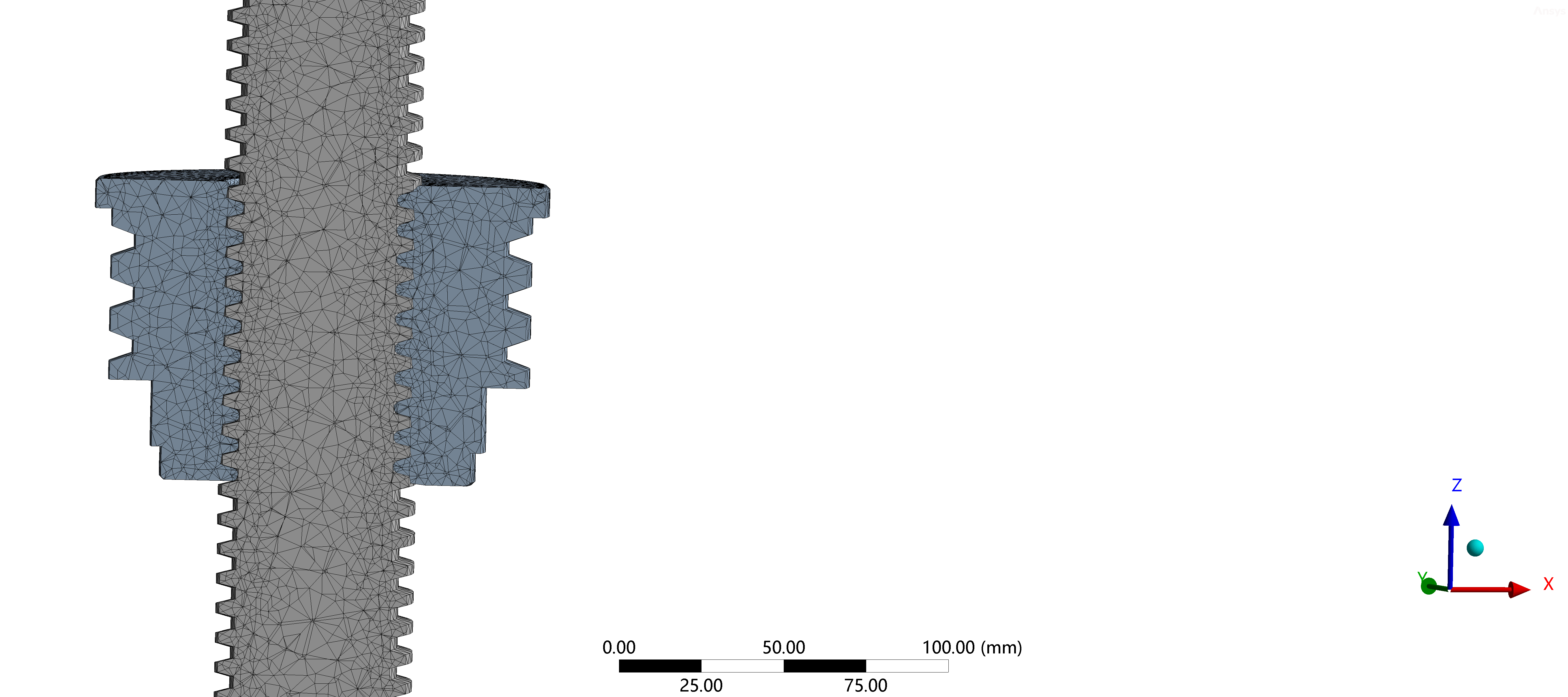

Supplement: Supplementary file 1 — Supplementary Information. [file 41598_2025_94144_MOESM1_ESM.zip › Simulation experiment result graph/Grid division result diagram/网格6.5.png]

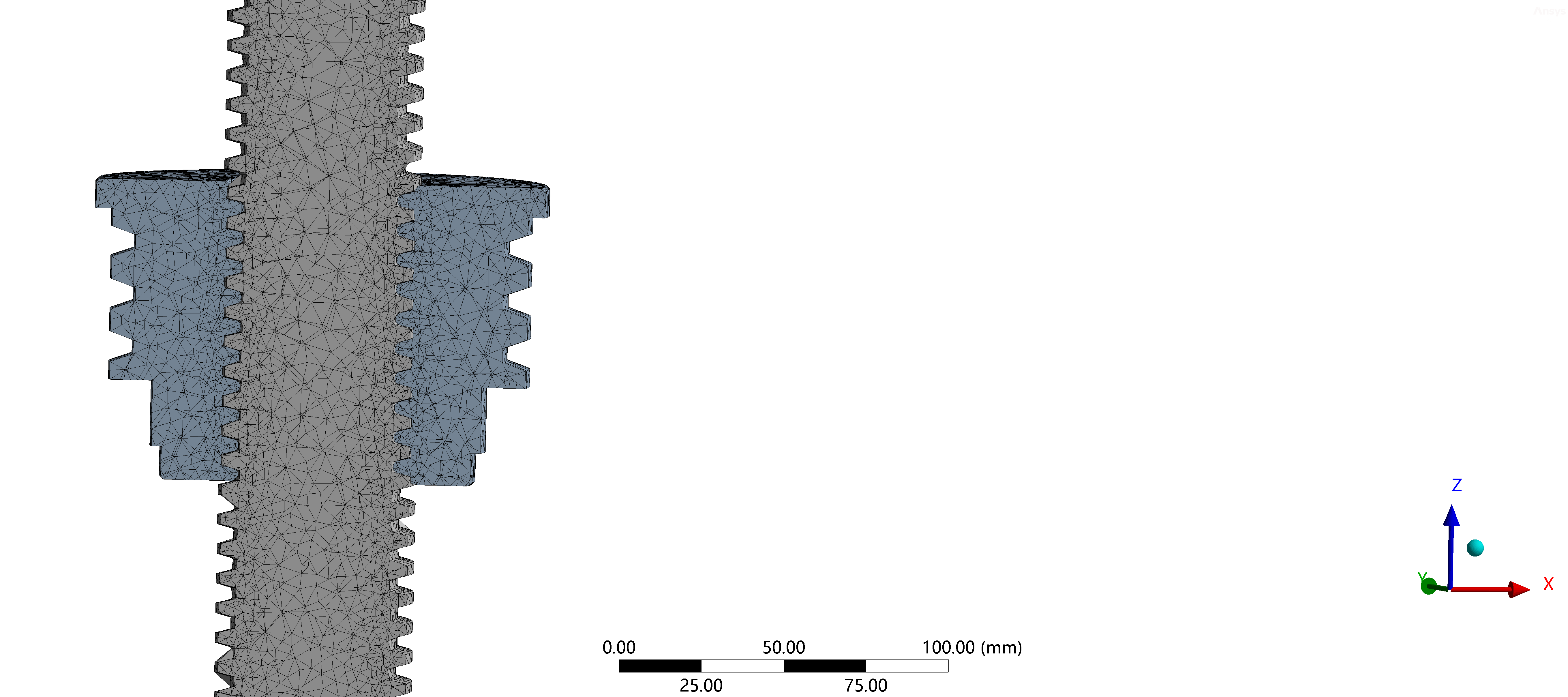

Supplement: Supplementary file 1 — Supplementary Information. [file 41598_2025_94144_MOESM1_ESM.zip › Simulation experiment result graph/Grid division result diagram/网格6.png]

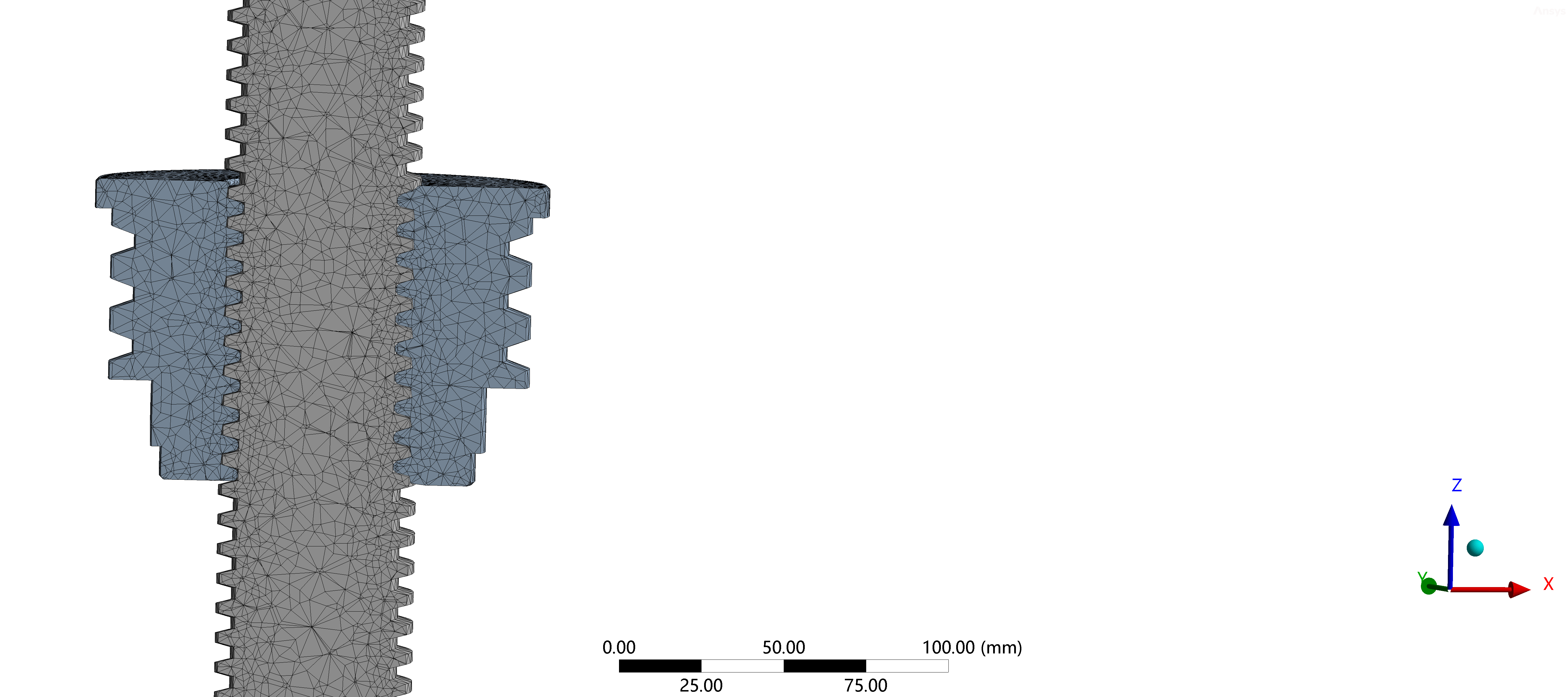

Supplement: Supplementary file 1 — Supplementary Information. [file 41598_2025_94144_MOESM1_ESM.zip › Simulation experiment result graph/Grid division result diagram/网格7.png]

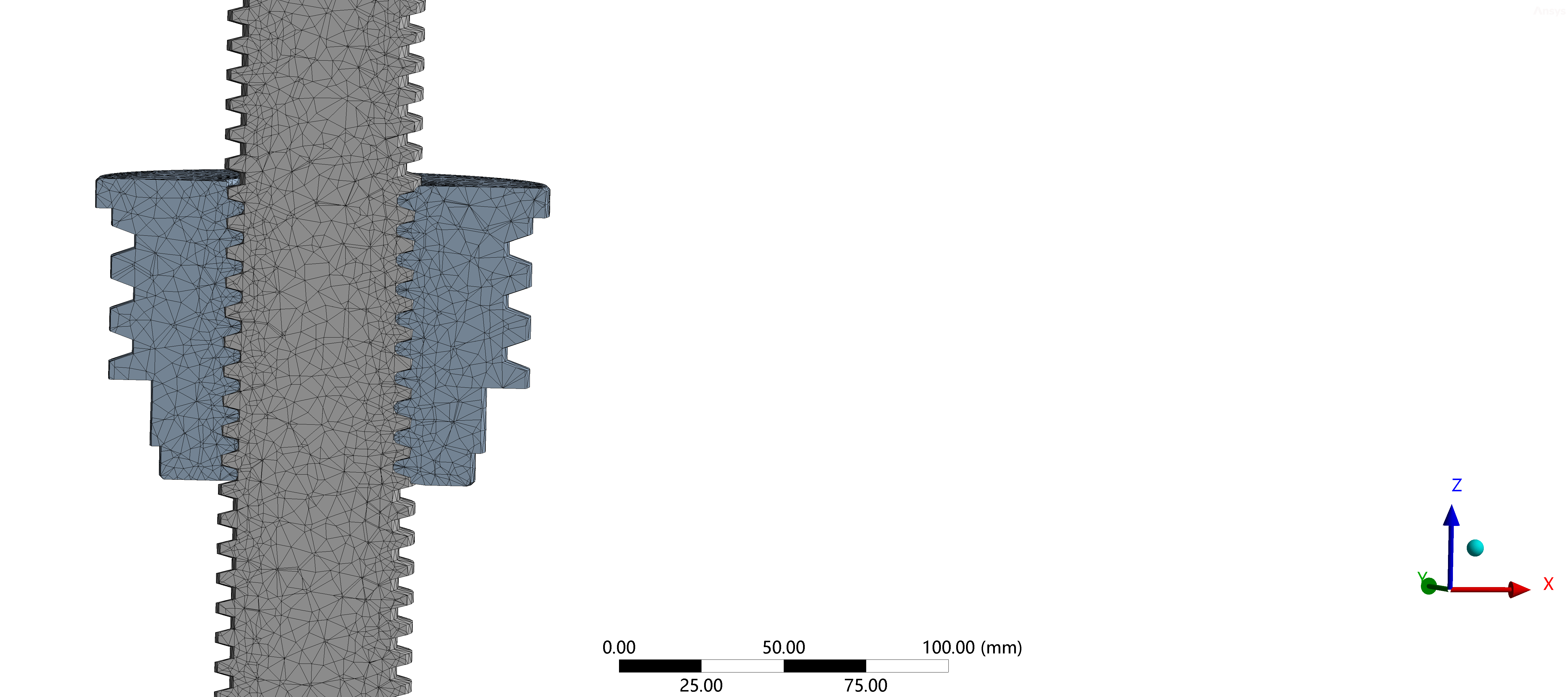

Supplement: Supplementary file 1 — Supplementary Information. [file 41598_2025_94144_MOESM1_ESM.zip › Simulation experiment result graph/Grid division result diagram/网格8.png]

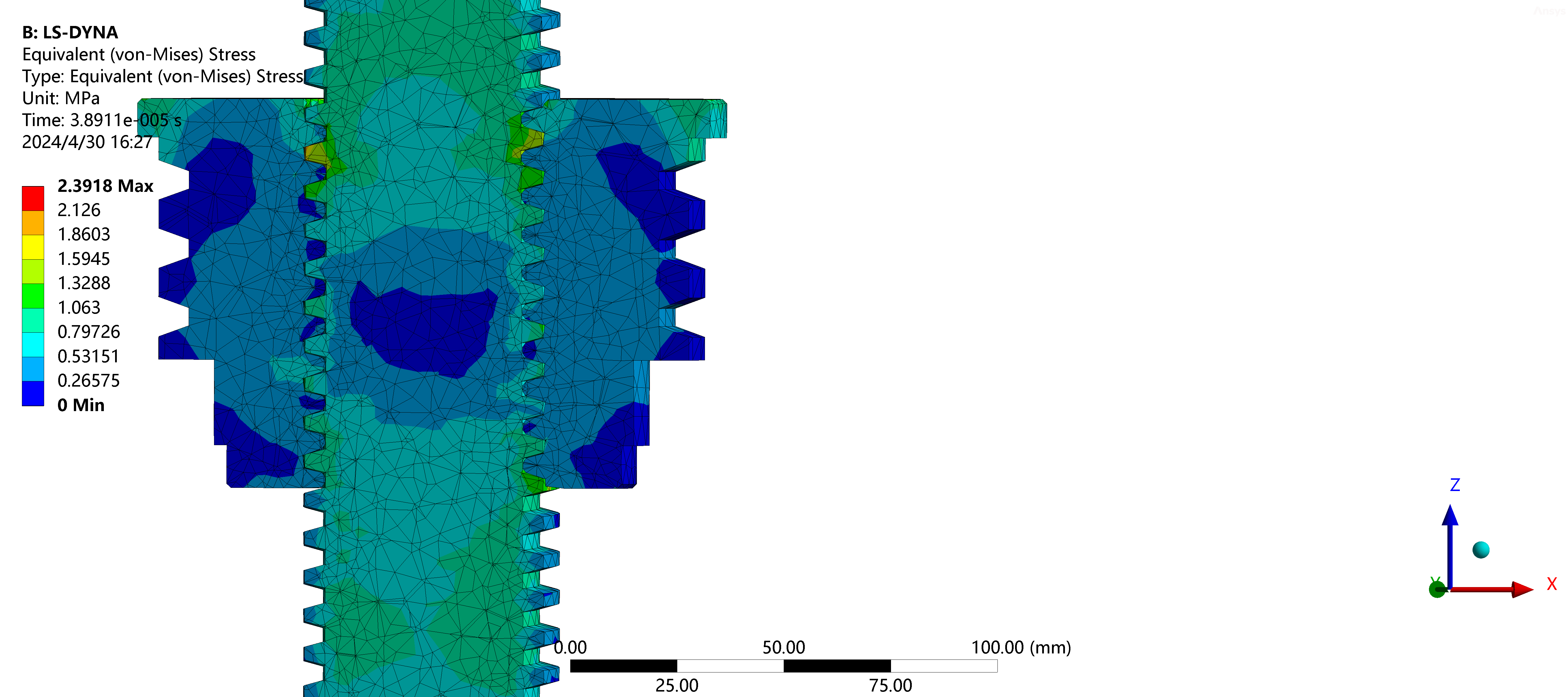

Supplement: Supplementary file 1 — Supplementary Information. [file 41598_2025_94144_MOESM1_ESM.zip › Simulation experiment result graph/Simulation plots before and after orthogonal optimization/应力Y.png]

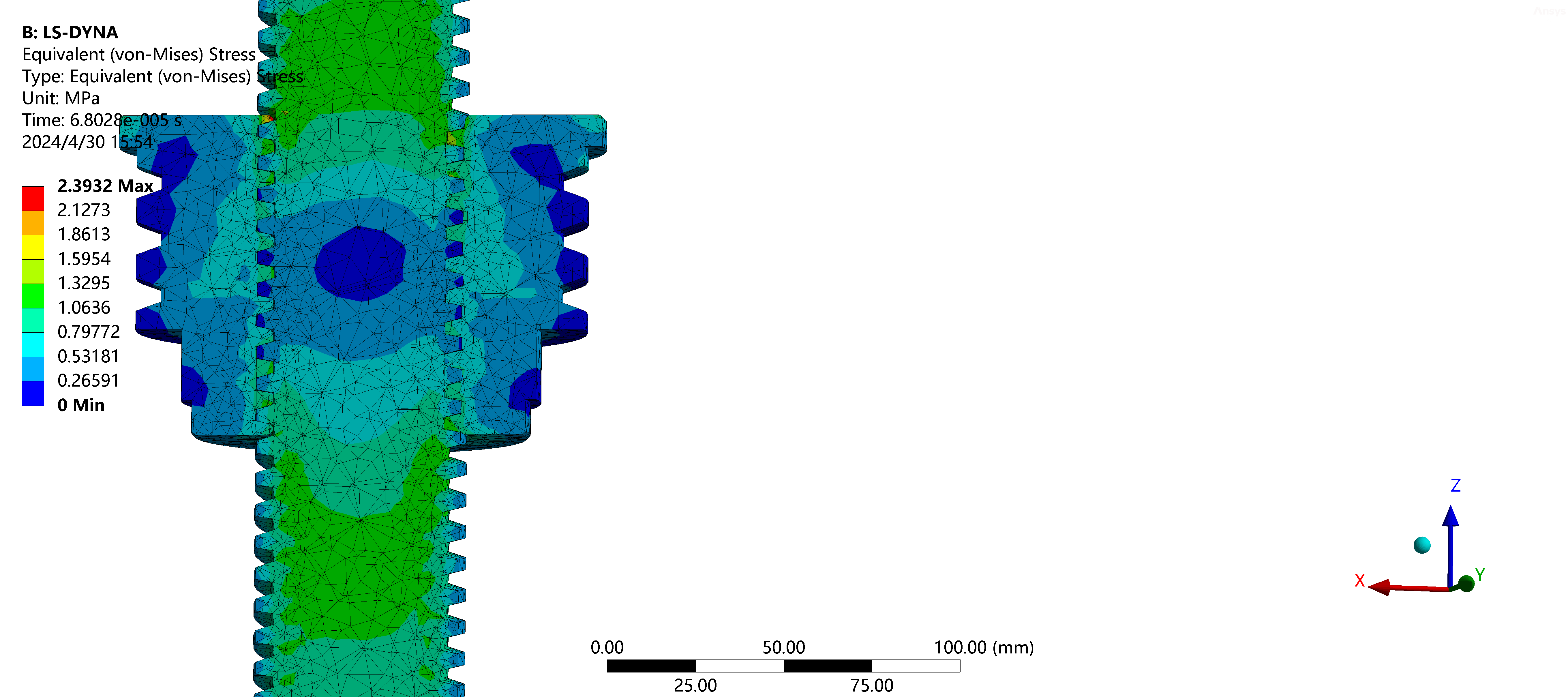

Supplement: Supplementary file 1 — Supplementary Information. [file 41598_2025_94144_MOESM1_ESM.zip › Simulation experiment result graph/Simulation plots before and after orthogonal optimization/应力Y1.png]

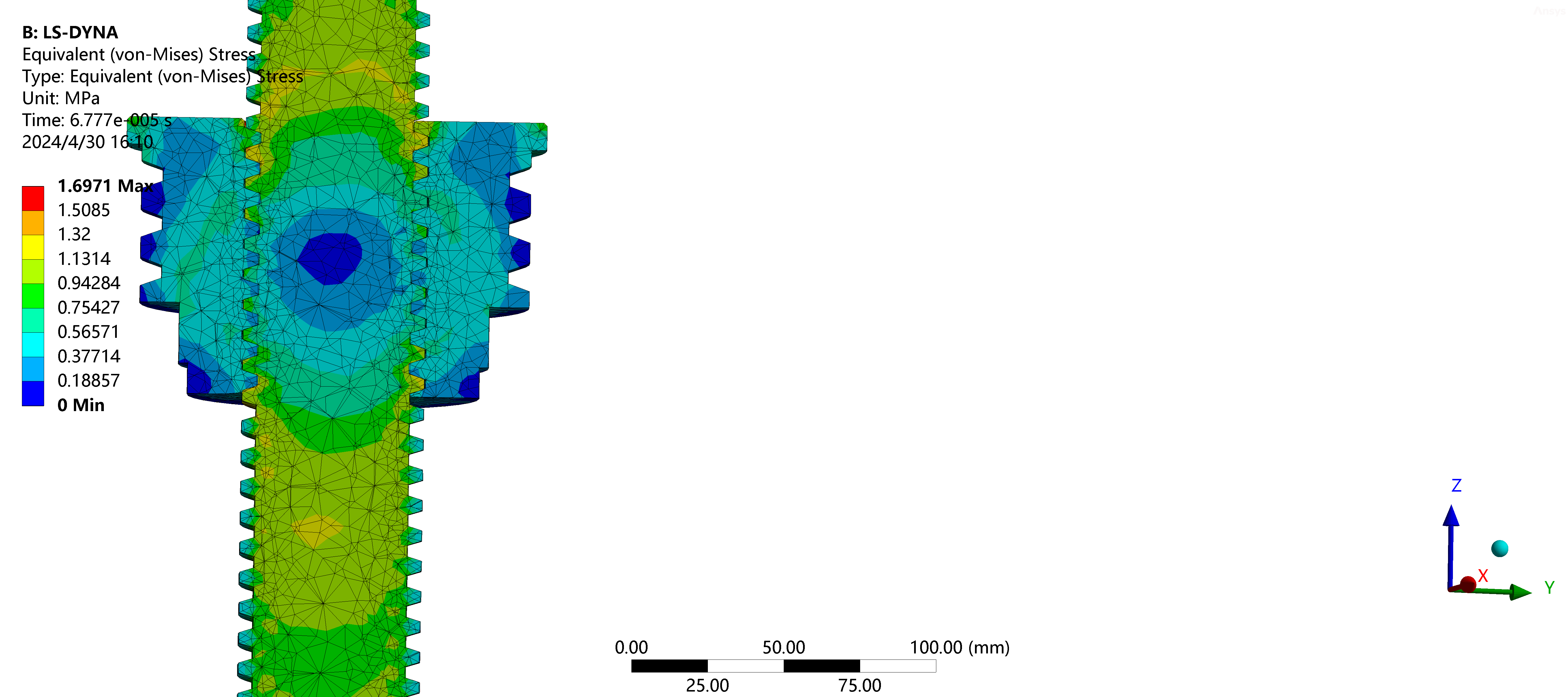

Supplement: Supplementary file 1 — Supplementary Information. [file 41598_2025_94144_MOESM1_ESM.zip › Simulation experiment result graph/Simulation plots before and after orthogonal optimization/应力Y2.png]

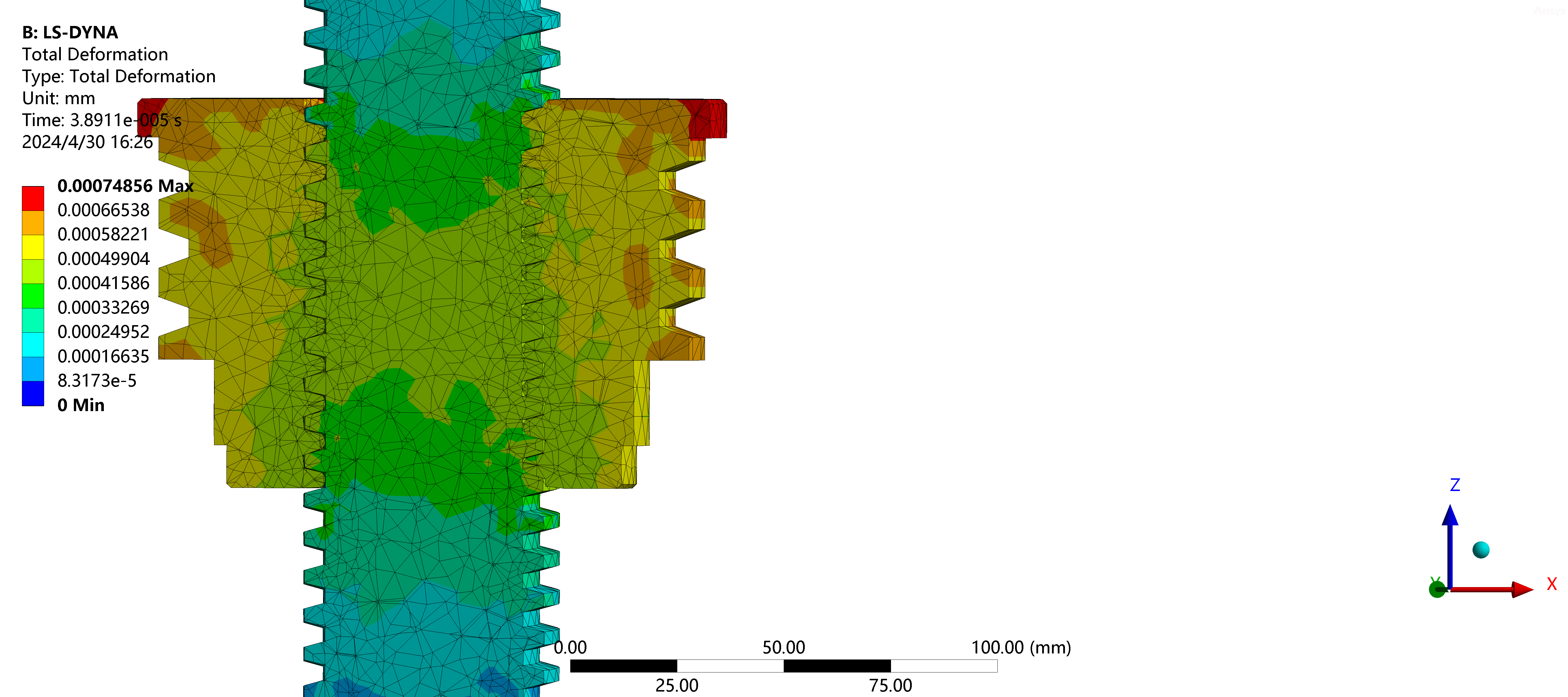

Supplement: Supplementary file 1 — Supplementary Information. [file 41598_2025_94144_MOESM1_ESM.zip › Simulation experiment result graph/Simulation plots before and after orthogonal optimization/总变形Y.png]

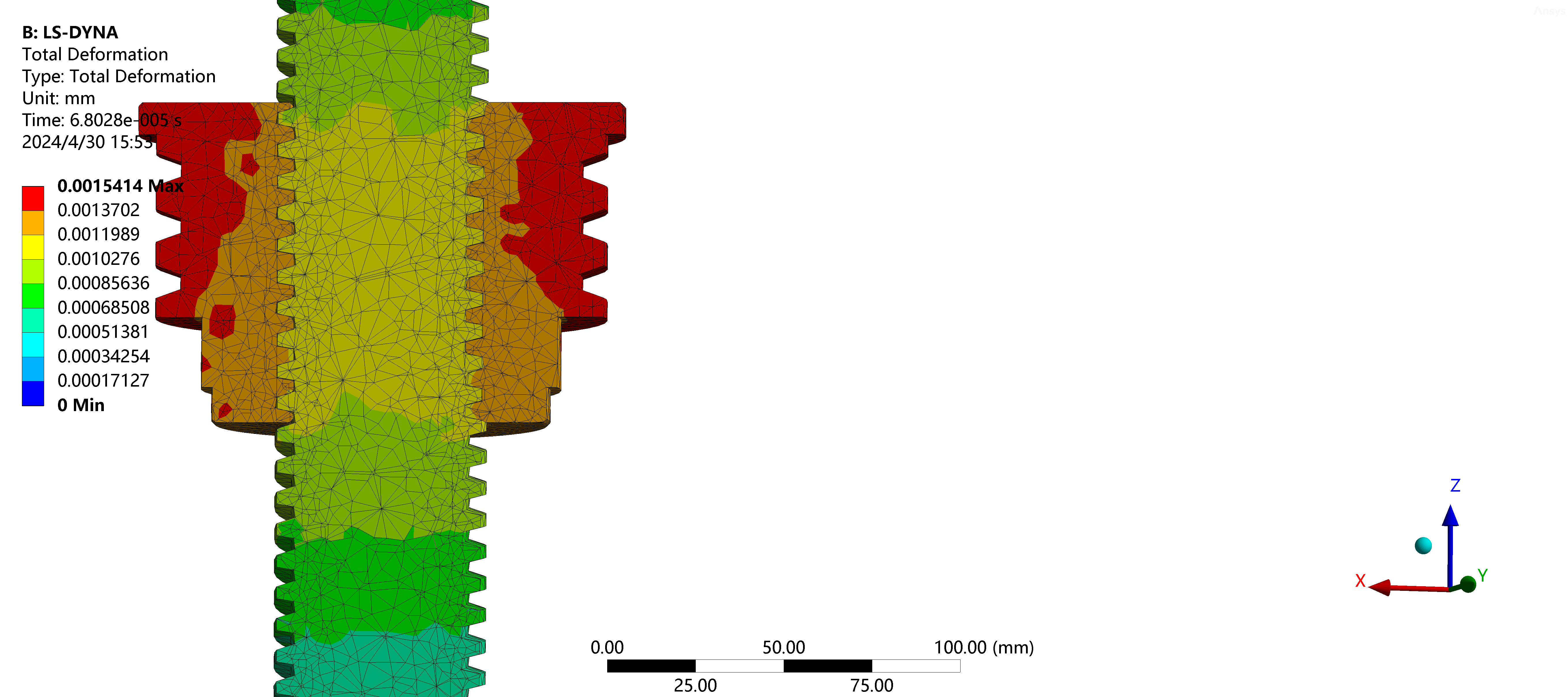

Supplement: Supplementary file 1 — Supplementary Information. [file 41598_2025_94144_MOESM1_ESM.zip › Simulation experiment result graph/Simulation plots before and after orthogonal optimization/总变形Y1.png]

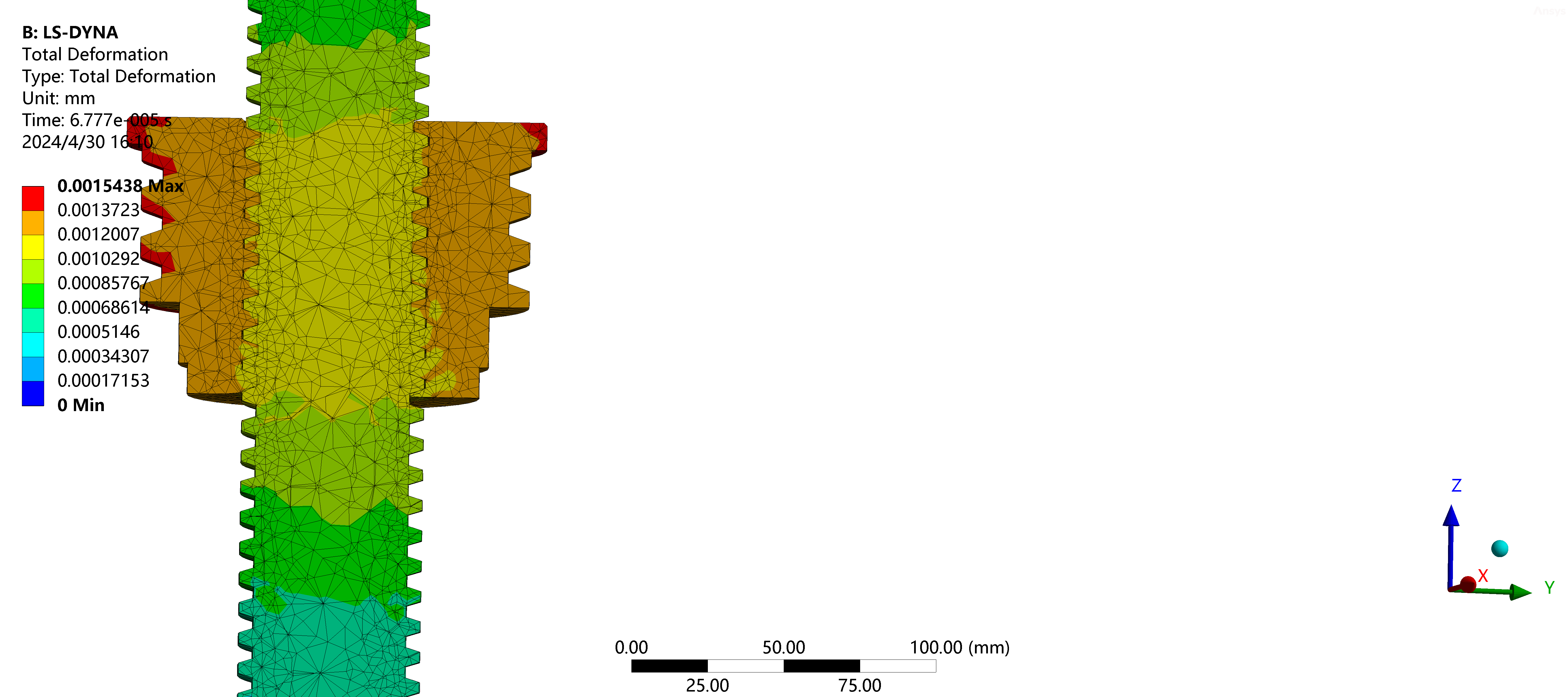

Supplement: Supplementary file 1 — Supplementary Information. [file 41598_2025_94144_MOESM1_ESM.zip › Simulation experiment result graph/Simulation plots before and after orthogonal optimization/总变形Y2.png]

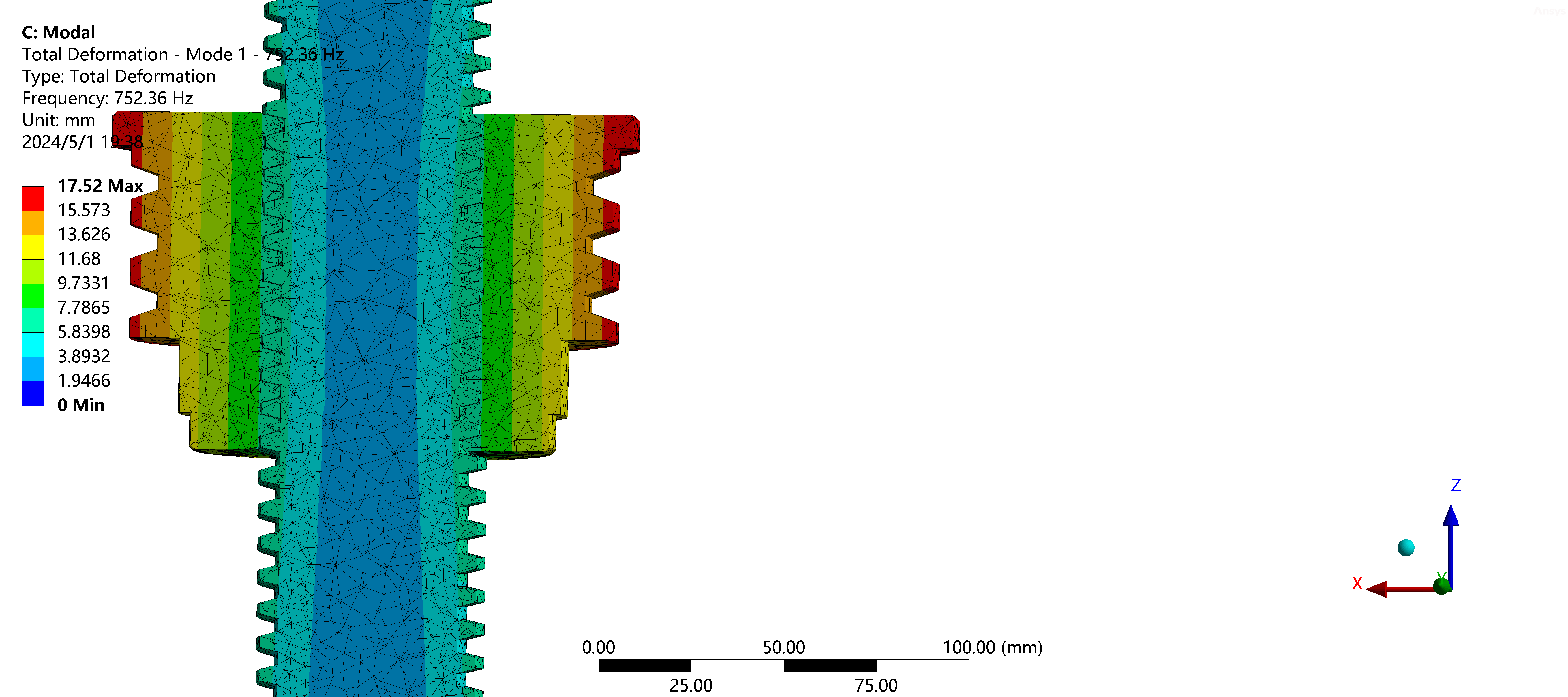

Supplement: Supplementary file 1 — Supplementary Information. [file 41598_2025_94144_MOESM1_ESM.zip › Simulation experiment result graph/Simulation plots before and after orthogonal optimization/频率Y.png]

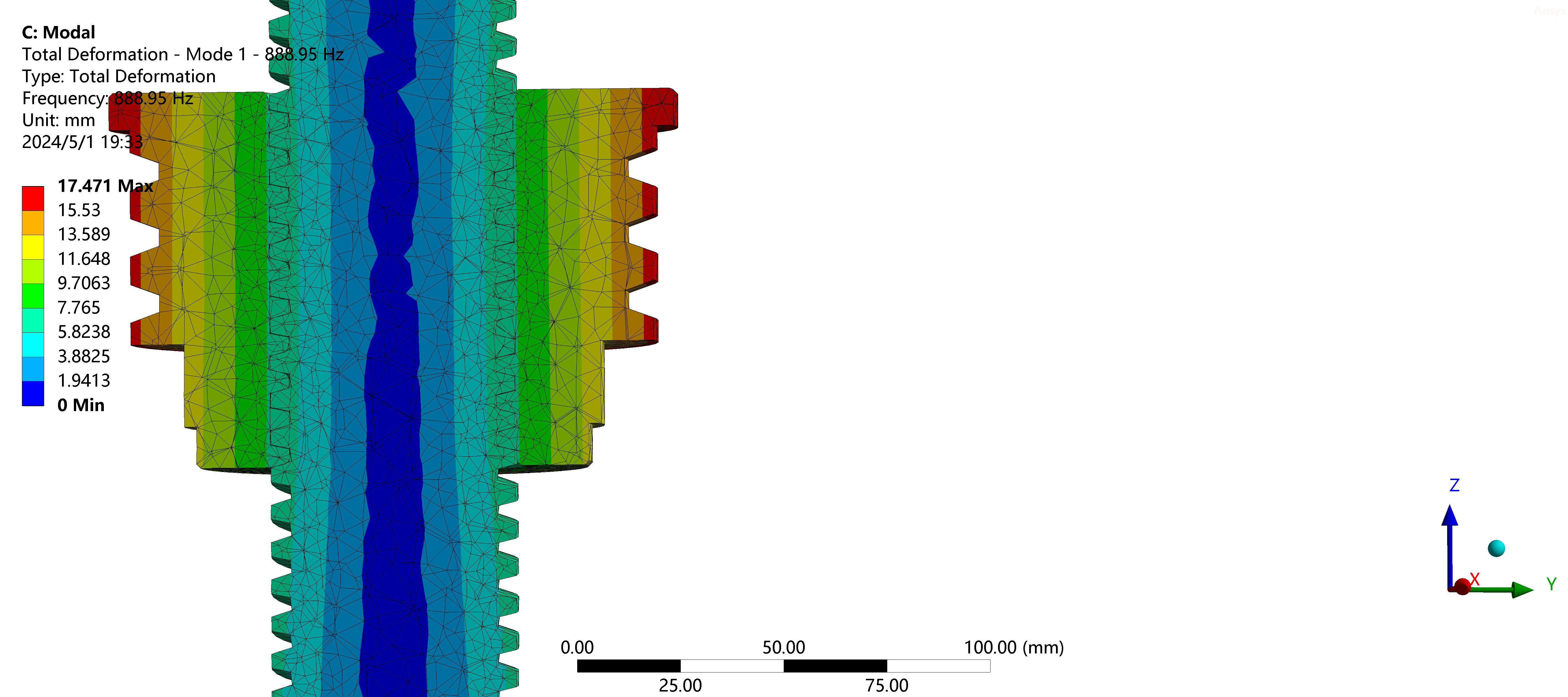

Supplement: Supplementary file 1 — Supplementary Information. [file 41598_2025_94144_MOESM1_ESM.zip › Simulation experiment result graph/Simulation plots before and after orthogonal optimization/频率Y1-1.png]

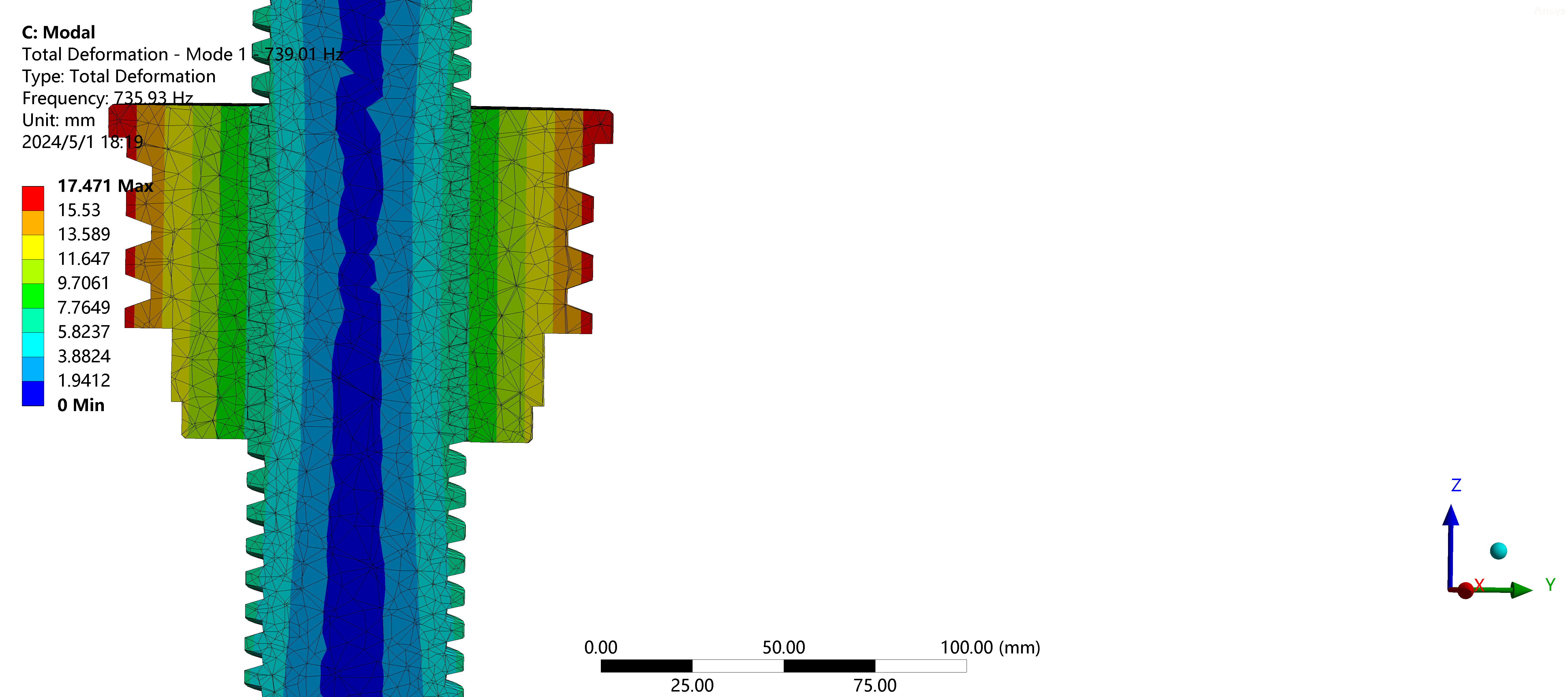

Supplement: Supplementary file 1 — Supplementary Information. [file 41598_2025_94144_MOESM1_ESM.zip › Simulation experiment result graph/Simulation plots before and after orthogonal optimization/频率Y1.png]

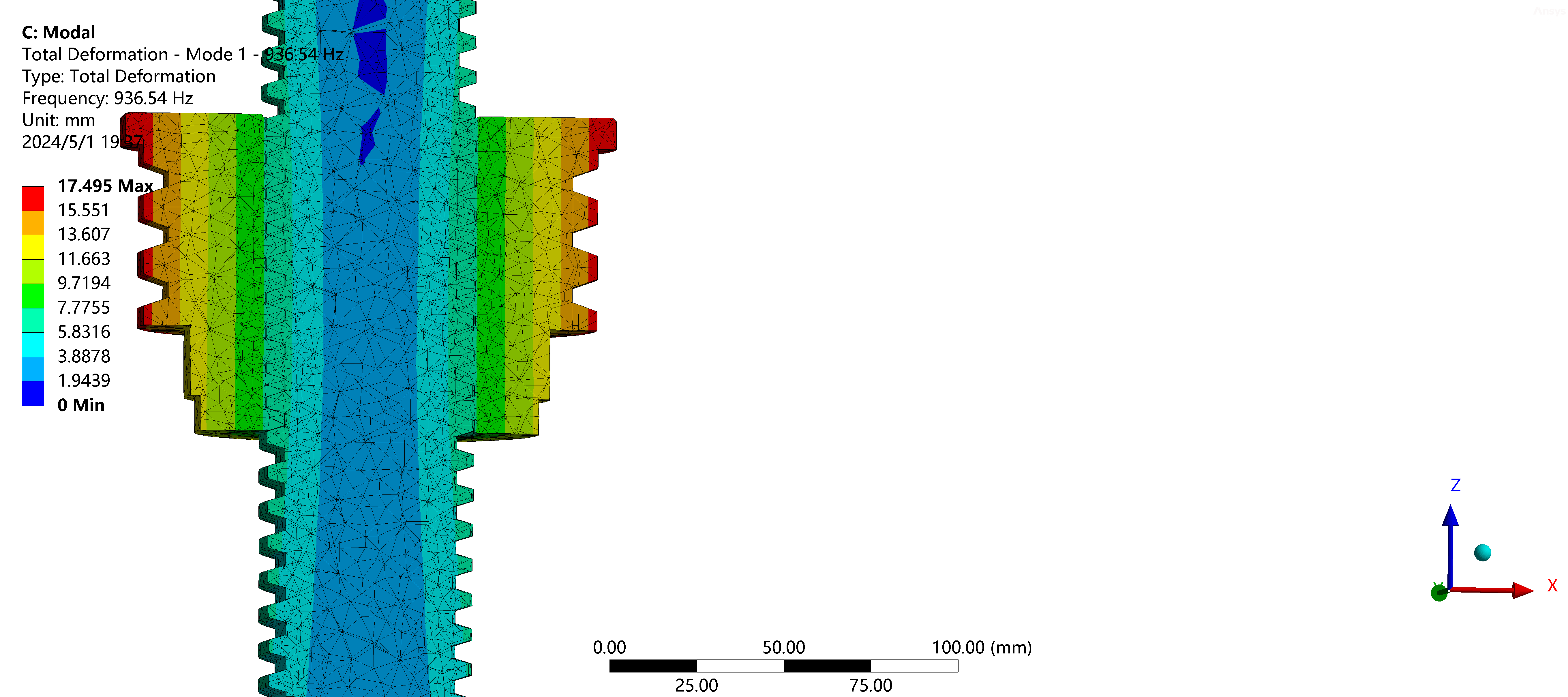

Supplement: Supplementary file 1 — Supplementary Information. [file 41598_2025_94144_MOESM1_ESM.zip › Simulation experiment result graph/Simulation plots before and after orthogonal optimization/频率Y2-1.png]

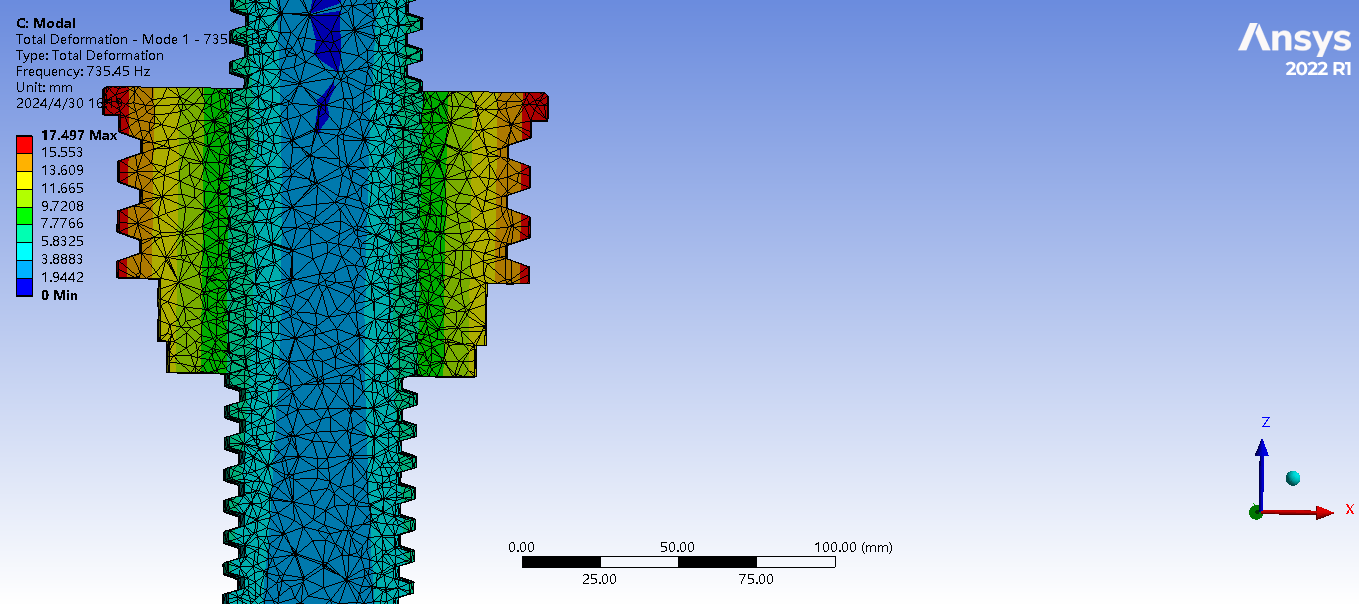

Supplement: Supplementary file 1 — Supplementary Information. [file 41598_2025_94144_MOESM1_ESM.zip › Simulation experiment result graph/Simulation plots before and after orthogonal optimization/频率Y2.png]
